# Global Prevalence of Adolescent Use of Nonprescription Weight-Loss Products: A Systematic Review and Meta-Analysis

**DOI:** 10.1001/jamanetworkopen.2023.50940

**Published:** 2024-01-10

**Authors:** Natasha Yvonne Hall, Dhanushi Madhushani Hetti Pathirannahalage, Cathy Mihalopoulos, S. Bryn Austin, Long Le

**Affiliations:** 1 School of Health and Social Development, Deakin University, Melbourne, Australia; 2 Public Health and Preventative Medicine, Monash University, Melbourne, Australia; 3 School of Public Health, Department of Social and Behavioral Sciences, Harvard University, Boston, Massachusetts

## Abstract

**Importance:**

Use of nonprescribed weight-loss products in adolescents is a public health concern that is associated with negative physical and psychological consequences. However, the prevalence of nonprescribed weight-loss product use in adolescents is unknown.

**Objective:**

To determine the global prevalence of nonprescription weight-loss product use in children and adolescents.

**Data Sources:**

Four databases, including MEDLINE, PsycINFO, CINAHL (Cumulative Index of Nursing and Allied Health), and EMBASE, were searched for quantitative studies that reported prevalence data on use of nonprescription weight-loss products with no restrictions on publication date. The search was performed December 1, 2020, and updated March 6, 2023.

**Study Selection:**

Studies were included in the meta-analysis if they reported the prevalence of weight-loss product use, were published in English, and included individuals 18 years or younger.

**Data Extraction and Synthesis:**

Data extraction was completed by 2 independent reviewers. Data analysis determined the overall pooled proportion of weight-loss product use in total and during the past week, past 30 days, past year, or lifetime. Inverse variance heterogeneity effect models were used.

**Main Outcomes and Measures:**

The main outcome measure was the prevalence of nonprescription weight-loss product use in adolescents for all included studies and over the past week, past month, past year, or lifetime. Subgroup analysis included separation of groups by sex, specific weight-loss product types, geographical location, and study publication time.

**Results:**

A total of 90 articles (604 552 unique participants) were included in the meta-analysis. Of these, 50 studies (56%) were from North America. The reported prevalence of weight-loss product use in adolescents was 5.5% (96% CI, 5.5%-5.6%) overall. When identifying use of weight-loss products in the general population, prevalence was 2.0% (95% CI, 1.9%-2.1%) in the past week, 4.4% (95% CI, 4.3%-4.5%) in the past month, 6.2% (95% CI, 6.1%-6.3%) in the past year, and 8.9% (95% CI, 8.6%-9.2%) in their lifetime. Use of weight-loss products was higher among girls than boys.

**Conclusions and Relevance:**

This meta-analysis found that use of weight-loss products occurs at high levels in adolescents, especially girls. These findings suggest that, given the ineffectiveness of these products for weight loss coupled with their harmful long-term health consequences, interventions are required to reduce use of weight-loss products in this group.

## Key Points

**Question:**

What is the prevalence of use of nonprescribed and medically unapproved weight-loss products in adolescents?

**Findings:**

This meta-analysis that included 90 studies with 604 552 participants found the prevalence of weight-loss product use in adolescents was 6% overall. In subgroup analysis of the general adolescent population, prevalence of weight-loss product use was 2% in the past week, 4% in the past month, 6% in the past year, and 9% in their lifetime.

**Meaning:**

Almost 1 in 10 adolescents have used ineffective and potentially harmful nonprescribed weight-loss products in their lifetime, suggesting that interventions are required to reduce use of weight-loss products in this population.

## Teaser

This systematic review and meta-analysis assesses the global prevalence of unprescribed use of weight-loss products among children and adolescents.

## Introduction

The high prevalence of eating and weight-related problems during childhood and adolescence is of great concern, particularly since these ages include periods of rapid growth and development.^1,2^ Disordered eating and unhealthy weight control behaviors during childhood are associated with negative psychosocial problems.^3,4,5^ Furthermore, unhealthy weight control behaviors can contribute to increased body weight, which may contribute to negative physical health issues.^6^

A concerning unhealthy weight control behavior, and the focus of this meta-analysis, is the adolescent use of weight-loss products (including drugs and dietary supplements) for weight control or reduction without a physician’s prescription. The use of nonprescribed weight-loss products in adolescents is concerning because it increases the risk of unhealthful weight gain in adulthood,^7^ as well as being prospectively associated with elevated risk of being diagnosed with an eating disorder within several years of onset of use.^7,8,9^ Furthermore, longitudinal studies found that the nonprescribed use of weight-loss products was associated with low self-esteem,^10^ depression,^10^ and poor nutritional intake.^11^ Cross-sectional studies^12,13^ found an association between nonprescribed use of weight-loss products and substance use. The prevalence of weight-loss product use without a physician’s prescription in adolescents ranges from past week use of 1.6% in Australia^14^; past 30-day use of 6.1% in the US,^15^ 6.7% in Brazil,^16^ and 2.4% in South Korea^17^; and past year use of 3.7% in New Zealand^18^ and 3.5% in Israel.^19^ While many studies have reviewed weight-loss product use without a prescription in adolescents, no systematic review or meta-analysis, to our knowledge, has determined the overall proportion of adolescents using weight-loss products without a physician’s prescription. The aim of this meta-analysis, therefore, was to determine the prevalence of weight-loss product use among children and adolescents. This includes identifying subgroup prevalence with respect to sex, weight-loss product type, and country differences. Better understanding of the prevalence of weight-loss product use in adolescents and adolescent subgroups may allow for policy developments, further research, and targeted education strategies among particular at-risk groups.

## Methods

This meta-analysis protocol was developed per the Preferred Reporting Items for Systematic Reviews and Meta-Analyses (PRISMA) reporting guideline. The study was registered in the PROSPERO International Prospective Register of Systematic Reviews (CRD42021234113). All procedures contributing to this work complied with the ethical standards of the relevant national and institutional committees on human experimentation and with the Helsinki Declaration of 1975, as revised in 2008.^20^ Ethics approval was granted by Deakin University Human Research Ethics Committee.

### Data Source

A comprehensive literature search was completed in EMBASE and through the EBSCOhost platform for MEDLINE, PsycINFO, and CINAHL (Cumulative Index of Nursing and Allied Health) on December 1, 2020, and updated March 6, 2023. There were no restrictions placed on publication year (study year range of included publications was 1985-2023) or geographical region. Search terms were related to prevalence, children and adolescents, and weight-loss products and can be found in eAppendix 1 in Supplement 1. There were no significant differences in the search strategies for the different databases. Grey literature was searched via Google Scholar using the search terms and the reference list of included articles.

### Study Selection

Studies included in the meta-analysis were observational studies reporting the prevalence of weight-loss product use over a period of time (past week, past month, past year, or lifetime). For this meta-analysis, weight-loss products were defined as drugs and dietary supplements that were used for weight control without a prescription or a physician’s order. The weight-loss products included in this study were diuretics, laxatives, and diet pills. The full list of dietary supplements that were used for weight control are provided in eAppendix 1 in Supplement 1. Dietary supplements also had to be used for weight loss purposes and to be used without a physician’s prescription to be included in the meta-analysis. For studies that did not report whether the weight-loss product was taken on a physician’s orders, we assumed the survey was asking about over-the-counter drugs and dietary supplements. Studies were included if they were published in English and included children and adolescents 18 years or younger. Studies that included participants both younger than 18 years and those 18 years and older were included in the meta-analysis; however, only proportions of weight-loss products used by those 18 years or younger were included. If the proportion of weight-loss product use by those 18 years or younger was not identifiable, it was calculated by multiplying the weight-loss product use proportion by the percentage of those 18 years or younger in the total population. Studies were excluded from the meta-analysis if they focused on weight controlling practices other than nonprescription use of weight-loss products, unhealthy weight control practices in general, solely qualitative aspects of use of weight-loss products, or a study population older than 18 years. In addition, case series, conference abstracts, theses, and reviews were excluded.

### Data Extraction

After removing duplicates, titles and abstracts were screened, followed by full-text screening. Screening was completed by 2 independent reviewers (D.M.H.P. and L.L.), with discrepancies resolved via discussion among the review authors. A data extraction template was developed, and the following information was extracted for each study: authors, year of publication, country, sample description, study design, sample size, study setting, type of weight-loss product that was used without a prescription, prevalence of weight-loss product use, and duration over which prevalence was calculated. Included studies were assessed for internal validity and bias risk using the critical appraisal tool, Joanna Briggs Institute (JBI) Appraisal Checklist for reporting prevalence data.^21^ The JBI tool is found in eAppendix 2 in Supplement 1. The research team decided that good-quality studies were required to score 70% or greater (score of ≥7 of 9), moderate-quality studies needed to score 50% to less than 70% (score of 5 or 6 of 9), and poor-quality studies scored less than 50% (score of ≤4 of 9). These quality assessment threshold scores have been used in past reviews.^22^ Quality assessment was completed on all included studies by 2 independent reviewers (D.M. and L.L.). Any disputes relating to quality assessment between the reviewers were resolved by discussion.

### Statistical Analysis

Data analysis involved determining an overall pooled proportion estimate of weight-loss product use in adolescents from the 90 included studies.^8,10,14,15,16,17,18,19,23,24,25,26,27,28,29,30,31,32,33,34,35,36,37,38,39,40,41,42,43,44,45,46,47,48,49,50,51,52,53,54,55,56,57,58,59,60,61,62,63,64,65,66,67,68,69,70,71,72,73,74,75,76,77,78,79,80,81,82,83,84,85,86,87,88,89,90,91,92,93,94,95,96,97,98,99,100,101,102,103,104,105^ The studies included general population adolescent prevalence studies and populations among whom use of weight-loss products is known to be higher, including those with substance use,^12,13^ the LGBTQI (lesbian, gay, bisexual, transgender, queer, and intersex) community,^106^ adolescents with type 1 diabetes,^23^ those with known eating disorders,^107^ and elite athletes.^24^ Therefore, a second analysis to determine the prevalence of weight-loss product use in the general adolescent population was completed with the exclusion of these higher-risk populations. The results of these analyses present the prevalence for different time points (past week, past month, past year, and lifetime use). Another subgroup analysis was completed at the different time points for the general adolescent population to determine the prevalence of nonprescription weight-loss product use for different types of weight-loss products. The 3 different product groups included diet pills, laxatives, and diuretics. A second subgroup analysis in the general adolescent population reviewed nonprescription weight-loss product use by sex, by continents (Africa, Asia, Europe, North America, Oceania, and South America), and by study publication years (before 2000, after 2000, and after 2010). A meta-analysis could be completed only if there were 2 or more studies included in the subgroup. Significance testing between the subgroups was completed via the 95% CIs. The inverse variance heterogeneity (IVH) model was chosen for meta-analysis results because it is an estimator model that uses a fixed effects model assumption that has a quasi–likelihood-based variance structure, which is considered more robust than other models.^108^ In contrast, the random effects model underestimates statistical error and therefore produces overconfident results, while the IVH model does not have the same statistical error issues.^108^ Furthermore, the IVH model can better handle heterogeneity than other meta-analysis models.^108^ Data analysis was conducted with Office Excel, version 2019 (Microsoft Corportion), using the MetaXL add-on software package.^109^ Statistical heterogeneity between the studies was evaluated using the *I*^2^ statistic and Cochran Q test. Heterogeneity was considered an issue if the *I*^2^ statistic was greater than 40% and/or the Q statistic was significant at 2-sided *P* = .01.^110^ The Doi plot asymmetry index (LFK index) was used to assess publication bias, with an LFK index of ±1 meaning no asymmetry, greater than ±1 to ±2 meaning minor asymmetry, and greater than ±2 implying major asymmetry.^109^

## Results

**Figure 1.**
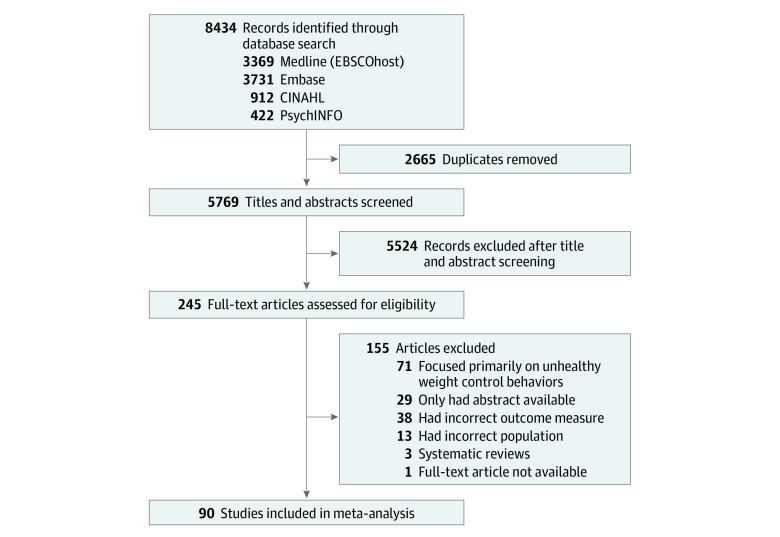
Study Diagram

A total of 90 studies^8,10,14,15,16,17,18,19,23,24,25,26,27,28,29,30,31,32,33,34,35,36,37,38,39,40,41,42,43,44,45,46,47,48,49,50,51,52,53,54,55,56,57,58,59,60,61,62,63,64,65,66,67,68,69,70,71,72,73,74,75,76,77,78,79,80,81,82,83,84,85,86,87,88,89,90,91,92,93,94,95,96,97,98,99,100,101,102,103,104,105^ met the inclusion criteria and were therefore included in the meta-analysis. Figure 1 shows the PRISMA flow diagram.

Sixty-nine studies (77%) reported weight-loss product use in both boys and girls,^8,10,14,15,16,17,18,19,23,24,26,28,29,30,31,32,33,34,35,36,37,38,39,40,41,42,43,44,45,46,47,48,49,50,51,52,53,54,55,56,57,58,59,60,62,64,65,66,67,68,69,76,78,82,84,86,87,90,91,93,95,96,97,98,100,102,104,105,111,112^ while 21 studies (23%) reported use of nonprescription weight-loss products in girls only. The sample size of the included studies ranged from 80 to 65 529, with the meta-analysis base case (all use of nonprescription weight-loss products at all time periods) containing data from 604 552 participants. The study year of publication ranged from 1985 to 2023, with 73 (81%) being published after year 2000. The mean (SD) age of participants across the studies ranged from 12.8 to 18.0 years. The 90 studies were from 25 different countries and 6 different continents. Two-thirds of the studies included reported prevalence in the general population, while the remaining studies focused on higher-risk groups (eg, with diabetes, substance use, diagnosed eating disorder). Fifty of the included studies (56%) were from North America (including the US, Canada, Jamaica, and Caribbean islands), 11 (12%) were from Asia (including Korea, China, Japan, and India), 11 (12%) were from Europe (including the United Kingdom, Romania, Italy, Norway, Spain, Belgium, and Denmark), 6 (7%) were from South America (including Brazil and Colombia), 5 (6%) were from the Middle East (Saudi Arabia, Iran, and Israel), 4 (4%) were from Africa (including South Africa, Ethiopia, and Mauritius), and 3 (3%) were from Oceania (Australia and New Zealand).^8,10,15,23,24,29,30,31,32,33,34,35,36,37,38,39,40,41,42,43,44,51,53,59,60,61,62,64,68,69,73,74,75,76,78,79,82,84,85,87,90,92,95,97,98,99,102,104,105,111^ The eTable in Supplement 1 gives study specific country detail. More than three-quarters of the included studies reported the prevalence of diet pill use (69 [77%]), followed by laxatives (41 [46%]) and diuretics (18 [20%]); studies could report use of multiple types of weight-loss products.

### Quality Assessment of the Included Studies

The JBI quality checklist^21^ determined that most of the studies were of moderate quality (46 [51%])^14,16,17,23,28,30,31,32,35,36,39,40,41,44,47,49,52,53,54,55,56,57,59,60,61,62,63,65,67,71,72,76,78,80,81,84,88,91,92,93,94,95,97,101,103,105^ or good quality (41 [46%]).^8,10,15,18,19,24,29,33,34,37,42,43,45,46,48,50,51,58,64,66,68,69,70,73,74,75,79,82,83,85,86,87,89,90,96,98,99,100,102,104,111^ Three studies (3%) were of poor quality.^25,26,27^ No studies were excluded from the main meta-analysis based on the JBI score. The quality assessment score for each study is in eTable in Supplement 1.

### Meta-Analysis Base Case Results

The pooled prevalence for weight-loss product use in adolescents was 5.5% (95% CI, 5.5%-5.6%; *I*^2^ = 100%) with detailed results reported in eFigure 1 in Supplement 1. Publication bias reported no asymmetry (LFK = −0.05). The Doi plot results are provided in eFigure 2 in Supplement 1.

### Meta-Analysis Results by Period of Reporting

**Figure 2.**
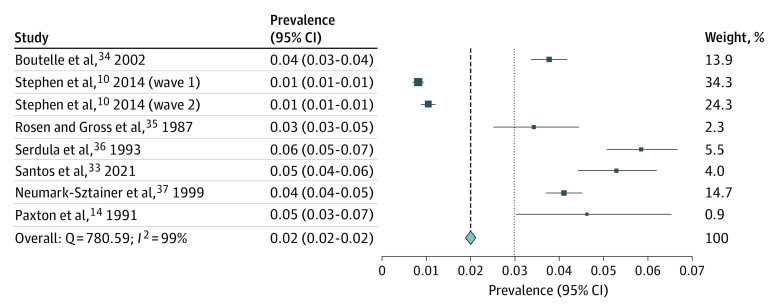
Prevalence of Nonprescription Weight-Loss Product Use in Past Week Squares represent the prevalence of weight-loss product use among adolescents for each study; the diamond represents overall prevalence. The size of each square corresponds to the size of the respective subsample.

**Figure 3.**
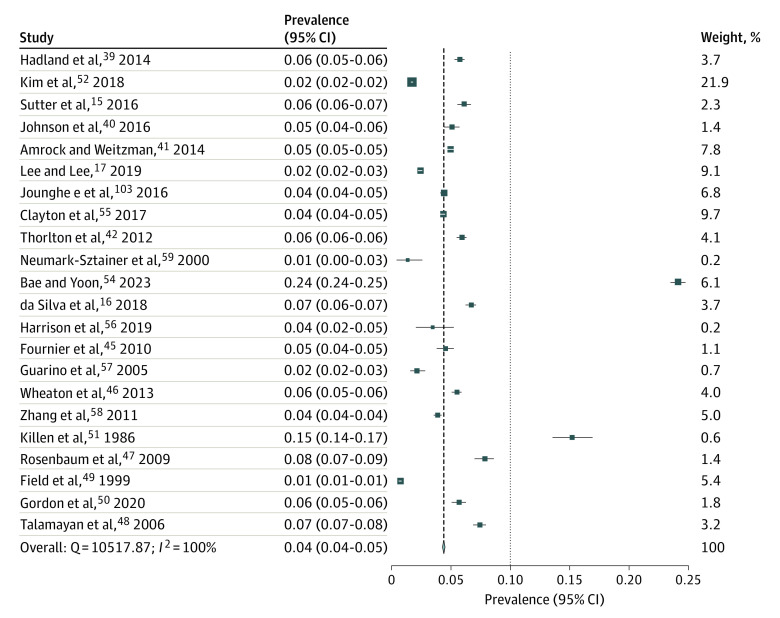
Prevalence of Nonprescription Weight-Loss Product Use in Past Month Squares represent the prevalence of weight-loss product use among adolescents for each study; the diamond represents overall prevalence. The size of each square corresponds to the size of the respective subsample.

The pooled prevalence of nonprescription weight-loss product use in adolescents over various time periods (past week, past month, past year, and lifetime) was conducted for the general population of adolescents only. Figure 2 shows that 2.0% (95% CI, 1.9%-2.1%; *I*^2^ = 99%) of adolescents used any nonprescription weight-loss products (ie, diet pills, laxatives, or diuretics) in the last week. Seven studies^10,14,28,29,30,31,32^ were included in the meta-analysis; 5 studies^10,29,30,31,32^ were from the US while 1 study each was from Brazil^28^ and Australia.^14^ Six of the studies were cross-sectional^14,28,29,30,31,32^ and 1 was longitudinal^10^ with both the wave 1 (1994) and wave 2 (1996) time points included in the meta-analysis. One of the studies included girls only.^29^ Two of the studies had a good JBI score,^10,29^ and the remaining 5 studies had a moderate JBI score.^14,28,30,31,32^ Publication bias is reported in eFigure 3 in Supplement 1 and reported major asymmetry (LFK = 5.44). Second, Figure 3 shows that 4.4% (95% CI, 4.3%-4.5%; *I*^2^ = 100%) of adolescents used nonprescription weight-loss products in the past 30 days. All 22 included studies^15,16,17,33,34,35,36,39,40,41,42,43,44,45,46,47,48,49,50,86,111,112^ were cross-sectional studies and focused on both boys and girls. Fourteen studies (64%)^15,33,34,35,36,37,38,39,40,41,42,43,44,111^ were from the US. Four studies (18%)^17,45,46,113^ were from Korea, with 1 study each from Colombia,^47^ Brazil,^16^ Jamaica,^48^ and Italy^49^ and a combined study from both the US and China.^50^ Most studies had good JBI scores, with 12 studies achieving good scores^15,33,34,42,43,45,46,48,50,111,112,113^ and 9 studies achieving moderate JBI scores.^16,17,35,36,39,40,41,47,49^ The Doi plot for this meta-analysis result shows minor asymmetry (LKF index = 1.15) (eFigure 4 in Supplement 1).

**Figure 4.**
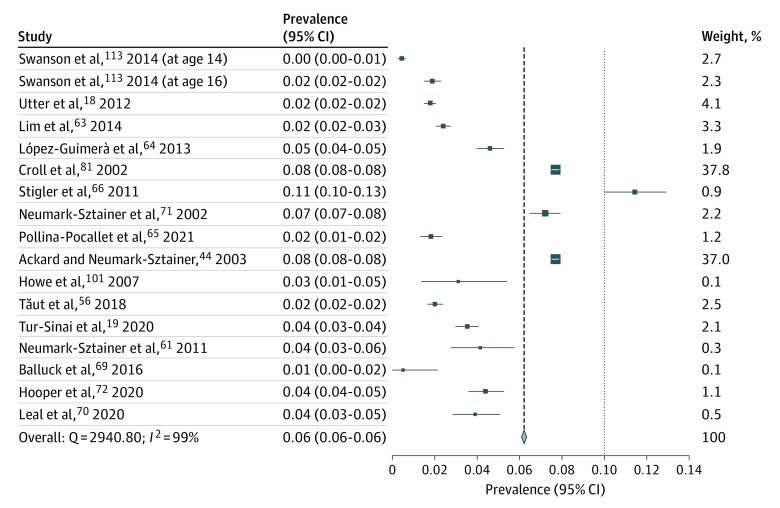
Prevalence of Nonprescription Weight-Loss Product Use in Past Year Squares represent the prevalence of weight-loss product use among adolescents for each study; the diamond represents overall prevalence. The size of each square corresponds to the size of the respective subsample.

**Figure 5.**
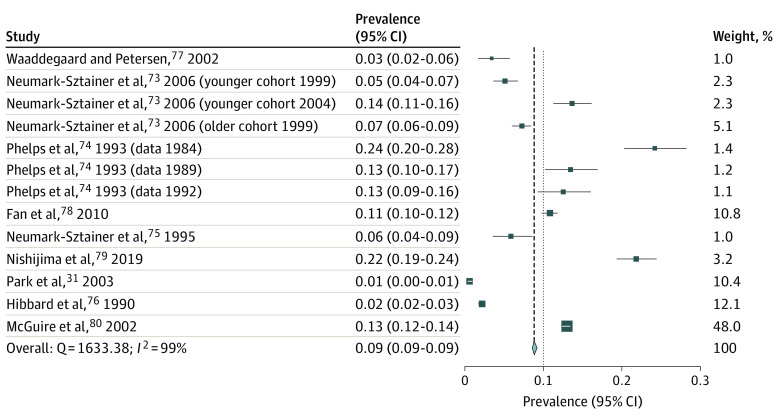
Prevalence of Nonprescription Weight-Loss Product Use in Lifetime Squares represent the prevalence of weight-loss product use among adolescents for each study; the diamond represents overall prevalence. The size of each square corresponds to the size of the respective subsample.

Figure 4 reports past-year prevalence of weight-loss product use in 16 studies^18,19,38,51,52,53,54,55,57,58,59,60,69,84,96^ and shows that 6.2% of participants (95% CI, 6.1%-6.3%; *I*^2 =^  99%; LFK index = −5.93) (eFigure 5 in Supplement 1) used nonprescription weight-loss products in the past year. Fourteen studies reported weight-loss product use in both boys and girls in the past year.^18,23,51,52,53,54,55,57,58,60,69,70,84,96^ All studies, except 1 longitudinal study,^51^ were cross-sectional studies. Seven of the studies were from the US,^23,51,53,60,69,70,84^ with the remaining 9 studies being from New Zealand,^18^ Israel,^19^ Korea,^52^ Spain,^53,54^ India,^55^ Romania,^56^ Mauritius,^57^ and Brazil.^58^ Seven studies^52,53,55,56,57,59,60^ had a moderate JBI score with the remainder having a good JBI score. Figure 5 shows that 8.9% of adolescents (95% CI, 8.6%-9.2%) had used nonprescription weight-loss products in their lifetime (*I*^2^ = 99%; LFK index = −0.91) (eFigure 6 in Supplement 1). Nine studies^26,61,62,63,64,65,66,67,68^ were included in the lifetime prevalence meta-analysis, with 2 studies having 3 separate weight loss prevalence points.^61,62^ All studies were cross-sectional, except for 1 cohort study.^61^ One of the included studies reviewed weight-loss products use in girls only.^63^ Three studies were from the US^61,62,64^; the other 6 studies were from Denmark,^65^ China,^66^ Israel,^63^ Japan,^67^ Korea,^26^ and Caribbean countries.^68^ Three studies had good JBI scores,^64,66,68^ and 1 study had a poor JBI score^26^; the remainder had moderate JBI scores.^61,62,63,65,67^

Subgroup analyses were completed in which sex, weight-loss product type used, publication year, and continent differences were reported separately. The meta-analysis graphs and a corresponding table of results for the subgroup analyses are reported in eFigures 7 through 10 in Supplement 1. Interestingly, studies published prior to 2000 had higher past-week prevalence yet lower lifetime prevalence than studies published after 2000 or studies published after 2010 (eFigure 8 in Supplement 1 provides further details). Lifetime prevalence for use of diet pills was 6.0% (95% CI, 6.0%-7.0%); for use of laxatives, 4.0% (95% CI, 4.0%-4.0%); and for use of diuretics, 2.0% (95% CI, 2.0%-2.0%).

eFigure 11 in Supplement 1 provides a sensitivity analysis in which the 3 poor-quality studies were removed from the main analysis. Results were identical when the poor-quality studies were removed.

## Discussion

This meta-analysis provides important insights into prevalence of weight-loss supplement use in adolescents with respect to weekly, monthly, yearly, and lifetime prevalence. However, it also provides prevalence points for differences in weight-loss product type used, sex, country, and publication year. This meta-analysis found that prevalence of use of diet pills (compared with laxatives and diuretics), female sex (compared with male), and North America (compared with Asia and Europe) were significantly higher, indicating that these populations are at higher risk. Overall use of nonprescription weight-loss products by adolescents was 5.3%; however, when high-risk individuals were excluded, this prevalence ranged from 2.0% in the past week to 8.9% in their lifetime. Nonprescribed weight-loss products in children are not medically recommended for healthy weight maintenance as they do not work, are dangerous, are associated with unhealthful weight gain in adulthood,^7,59^ and increase the risk of being diagnosed with an eating disorder within several years of onset of use.^7,8^ Furthermore, childhood use of nonprescribed weight-loss products has been associated with low self-esteem,^10^ depression,^10^ poor nutritional intake,^11^ and substance use.^12,13^

The prevalence of nonprescription use of weight-loss products was significantly higher in girls than boys at all time points (past week, past month, past year, and lifetime). Almost 1 in 10 adolescent girls had used a weight-loss product during their lifetime and in the past year. This is a public health concern because correlates between use of weight-loss products have been found with girls who have a low self-esteem,^69^ parental influence to lose weight or parental dissatisfaction with weight,^114^ self-body dissatisfaction,^115^ peer groups who value thinness,^116^ and media or social media influences promoting unrealistic beauty standards.^115^

The lifetime prevalence of diet pill use was the highest (6.0%), followed by use of laxatives (4.0%) and use of diuretics (2.0%). The lifetime prevalence of nonprescription diet pill use for weight control had a statistically significant (using 95% CIs) higher prevalence compared with both nonprescription use of laxatives and diuretics for weight loss. This is concerning because of the mental and physical health risks associated with the use of these medical products that are not indicated for weight loss but are often used as weight-loss products.^7,8,10,11,12,13^ Equally alarming is the ease of access of these products without a prescription, without a physician’s orders, and without restrictions or regulations for those 18 years or younger.^117,118^ This emphasizes the need for increased regulation and restriction to be placed on nonprescription weight-loss products, especially for individuals 18 years or younger.

Prevalence of nonprescribed weight-loss product use in the past year was statistically significantly higher in North America compared with Asia and Europe. Prevalence of weight-loss product use in the past year in Asia was statistically significantly higher compared with Europe. Similarly, lifetime prevalence of use of weight-loss products was significantly higher in North America compared with Asia. One study^53^ replicated this result and reported that unhealthy weight control behaviors were more common in North American adolescents compared with European adolescents.

### Limitations

This meta-analysis has several limitations. First, most of the studies were from North America, which may lead to bias and lack of certainty when generalizing to other regions. Second, while we determined the prevalence of nonprescription weight-loss product use, many of the included studies only implied and did not explicitly state that the weight-loss product use was nonprescribed. This may lead to bias and underreporting or overreporting of the prevalence results. Third, the low number of studies in some subgroups and the range of sample sizes of included studies alongside higher rates of disordered eating in girls may lead to bias in prevalence estimates and accuracy of the results.

## Conclusions

The findings of this meta-analysis suggest that overall use of nonprescription weight-loss products was 5.3%. In the general adolescent population, weight-loss product use ranged from 2.0% (past week) to 8.9% (lifetime use), and weight-loss product use was more common among girls. Diet pills were the weight-loss product used most frequently by adolescents, followed by laxatives and diuretics. Prevalence of weight-loss product use was higher in North America compared with Asia and Europe and was higher in Asia compared with Europe. Further research is required in African, South American, and Middle Eastern countries. Given the individual and public health issues associated with adolescent use of nonprescription weight-loss products, interventions are urgently required to prevent and regulate use of weight-loss products in this population.

## Notes

Supplement 1. **eAppendix 1.** MEDLINE, PsycINFO, CINAHL, and Embase Search Strategy for the Meta-Analysis **eAppendix 2.** The Joanna Briggs Institute Appraisal Checklist for Prevalence Studies **eTable.** Data Extraction Table for All 90 Included Meta-Analysis Studies **eFigure 1.** Weight Loss Product Use Among Adolescents **eFigure 2.** Doi Plot and LFK Index for Publication Bias for All 90 Studies **eFigure 3.** Doi Plot and LFK Index for Publication Bias for Weight Loss Product Use in the Past Week **eFigure 4.** Doi Plot and LFK Index for Publication Bias for Weight Loss Product Use in the Past Month **eFigure 5.** Doi Plot and LFK Index for Publication Bias for Weight Loss Product Use in the Past Year **eFigure 6.** Doi Plot and LFK Index for Publication Bias for Weight Loss Product Use in the Lifetime **eFigure 7.** Meta-Analysis Results for Gender Differences in Weight Loss Product Use **eFigure 8.** Meta-Analysis Results for Differences in Type of Weight Loss Product Use **eFigure 9.** Meta-Analysis Results for Continent Differences in Weight Loss Product Use **eFigure 10.** Meta-Analysis Results for Weight Loss Product Use With Respect to Different Study Publication Years **eFigure 11.** Meta-Analysis Results With the Removal of the 3 Poor Quality Studies (32, 55, and 79)
eReferences

Supplement 2.
Data Sharing Statement
