# Supplementary material for: Global Prevalence of Adolescent Use of Nonprescription Weight-Loss Products: A Systematic Review and Meta-Analysis
Source: JAMA Netw Open. 2024 Jan 10;7(1):e2350940. doi: 10.1001/jamanetworkopen.2023.50940 (PMC10782242; doi:10.1001/jamanetworkopen.2023.50940)
Supplement: Supplement 1. — eAppendix 1. MEDLINE, PsycINFO, CINAHL, and Embase Search Strategy for the Meta-Analysis eAppendix 2. The Joanna Briggs Institute Appraisal Checklist for Prevalence Studies eTable. Data Extraction Table for All 90 Included Meta-Analysis Studies eFigure 1. Weight Loss Product Use Among Adolescents eFigure 2. Doi Plot and LFK Index for Publication Bias for All 90 Studies eFigure 3. Doi Plot and LFK Index for Publication Bias for Weight Loss Product Use in the Past Week eFigure 4. Doi Plot and LFK Index for Publication Bias for Weight Loss Product Use in the Past Month eFigure 5. Doi Plot and LFK Index for Publication Bias for Weight Loss Product Use in the Past Year eFigure 6. Doi Plot and LFK Index for Publication Bias for Weight Loss Product Use in the Lifetime eFigure 7. Meta-Analysis Results for Gender Differences in Weight Loss Product Use eFigure 8. Meta-Analysis Results for Differences in Type of Weight Loss Product Use eFigure 9. Meta-Analysis Results for Continent Differences in Weight Loss Product Use eFigure 10. Meta-Analysis Results for Weight Loss Product Use With Respect to Different Study Publication Years eFigure 11. Meta-Analysis Results With the Removal of the 3 Poor Quality Studies (32, 55, and 79) eReferences [file jamanetwopen-e2350940-s001.pdf]

Supplementary Online Content

Hall NY, Madhushani D, Mihalopoulos C, Austin SB, Le L. Global prevalence of adolescent use of nonprescription weight-loss products. *JAMA Netw Open*. 2024;7(1):e2350940. doi:10.1001/jamanetworkopen.2023.50940

eAppendix 1. MEDLINE, PsycINFO, CINAHL, and Embase Search Strategy for the Meta-Analysis

eAppendix 2. The Joanna Briggs Institute Appraisal Checklist for Prevalence Studies

eTable. Data Extraction Table for All 90 Included Meta-Analysis Studies

eFigure 1. Weight Loss Product Use Among Adolescents

eFigure 2. Doi Plot and LFK Index for Publication Bias for All 90 Studies

eFigure 3. Doi Plot and LFK Index for Publication Bias for Weight Loss Product Use in the Past Week

eFigure 4. Doi Plot and LFK Index for Publication Bias for Weight Loss Product Use in the Past Month

eFigure 5. Doi Plot and LFK Index for Publication Bias for Weight Loss Product Use in the Past Year

eFigure 6. Doi Plot and LFK Index for Publication Bias for Weight Loss Product Use in the Lifetime

eFigure 7. Meta-Analysis Results for Gender Differences in Weight Loss Product Use

eFigure 8. Meta-Analysis Results for Differences in Type of Weight Loss Product Use

eFigure 9. Meta-Analysis Results for Continent Differences in Weight Loss Product Use

eFigure 10. Meta-Analysis Results for Weight Loss Product Use With Respect to Different Study Publication Years

eFigure 11. Meta-Analysis Results With the Removal of the 3 Poor Quality Studies (32, 55, and 79)

eReferences

This supplementary material has been provided by the authors to give readers additional information about their work.

eAppendix 1. MEDLINE, PsycINFO, CINAHL, and Embase Search Strategy for the Meta-Analysis

TI OR AB	Prevalenc* OR Proportion OR epidemiolog* OR Incidenc*	Search with AND
TI OR AB	Child* OR Adolescen* OR teenager* OR youth* OR “young* person”OR juvenil* OR kid* OR pediatric*	
TI OR AB	“weight loss” OR “diet* pill*” OR “weight control” OR CLA OR “conjugated linoleic acid” OR “green tea” OR glucomannan OR konjac OR lcarnitine OR “coleus forskohlii” OR synephrine OR “bitter orange” OR “gacinia cambogia” chromium OR psyllium OR capsaicin OR laxative* OR diuretic OR “unhealthy behavioural control” OR “Appetite suppressant*” OR chitosan OR fucoxanthin “guar gum” OR “Irvingia gabonensis” OR “Hoodia gordonii” OR “Phaseolus vulgaris” OR pyruvate OR “Raspberry ketone”	

eAppendix 2. The Joanna Briggs Institute Appraisal Checklist for Prevalence Studies

JBI Critical Appraisal Checklist for Studies Reporting Prevalence Data

	Yes	No	Unclear	Not applicable
1. Was the sample frame appropriate to address the target population?	☐	☐	☐	☐
2. Were study participants sampled in an appropriate way?	☐	☐	☐	☐
3. Was the sample size adequate?	☐	☐	☐	☐
4. Were the study subjects and the setting described in detail?	☐	☐	☐	☐
5. Was the data analysis conducted with sufficient coverage of the identified sample?	☐	☐	☐	☐
6. Were valid methods used for the identification of the condition?	☐	☐	☐	☐
7. Was the condition measured in a standard, reliable way for all participants?	☐	☐	☐	☐
8. Was there appropriate statistical analysis?	☐	☐	☐	☐
9. Was the response rate adequate, and if not, was the low response rate managed appropriately?	☐	☐	☐	☐

© 2024 Hall NY et al. *JAMA Network Open*.

Overall appraisal: Include ☐ Exclude ☐ Seek further info ☐

Comments (Including reason for exclusion)

JBI Critical Appraisal Checklist for Studies Reporting Prevalence Data

How to cite: Munn Z, Moola S, Lisy K, Riitano D, Tufanaru C. Methodological guidance for systematic reviews of observational epidemiological studies reporting prevalence and incidence data. Int J Evid Based Healthc. 2015;13(3):147–153.

Answers: Yes, No, Unclear or Not/Applicable

1. **Was the sample frame appropriate to address the target population?**

   This question relies upon knowledge of the broader characteristics of the population of interest and the geographical area. If the study is of women with breast cancer, knowledge of at least the characteristics, demographics and medical history is needed. The term "target population" should not be taken to infer every individual from everywhere or with similar disease or exposure characteristics. Instead, give consideration to specific population characteristics in the study, including age range, gender, morbidities, medications, and other potentially influential factors. For example, a sample frame may not be appropriate to address the target population if a certain group has been used (such as those working for one organisation, or one profession) and the results then inferred to the target population (i.e. working adults). A sample frame may be appropriate when it includes almost all the members of the target population (i.e. a census, or a complete list of participants or complete registry data).

2. **Were study participants recruited in an appropriate way?**

   Studies may report random sampling from a population, and the methods section should report how sampling was performed. Random probabilistic sampling from a defined subset of the population (sample frame) should be employed in most cases, however, random probabilistic sampling is not needed when everyone in the sampling frame will be included/ analysed. For example, reporting on all the data from a good census is appropriate as a good census will identify everybody. When using cluster sampling, such as a random sample of villages within a region, the methods need to be clearly stated as the precision of the final prevalence estimate incorporates the clustering effect. Convenience samples, such as a street survey or interviewing lots of people at a public gatherings are not considered to provide a representative sample of the base population.

3. **Was the sample size adequate?**

The larger the sample, the narrower will be the confidence interval around the prevalence estimate, making the results more precise. An adequate sample size is important to ensure good precision of the final estimate. Ideally we are looking for evidence that the authors conducted a sample size calculation to determine an adequate sample size. This will estimate how many subjects are needed to produce a reliable estimate of the measure(s) of interest. For conditions with a low prevalence, a larger sample size is needed. Also consider sample sizes for subgroup (or characteristics) analyses, and whether these are appropriate. Sometimes, the study will be large enough (as in large national surveys) whereby a sample size calculation is not required. In these cases, sample size can be considered adequate.

When there is no sample size calculation and it is not a large national survey, the reviewers may consider conducting their own sample size analysis using the following formula: (Naing et al. 2006, Daniel

1999) n=

Z2P(1-P) d2

Where:

n= sample size

Z = Z statistic for a level of confidence

P = Expected prevalence or proportion (in proportion of one; if 20%, P = 0.2) d

= precision (in proportion of one; if 5%, d=0.05)

Ref:

Naing L, Winn T, Rusli BN. Practical issues in calculating the sample size for prevalence studies Archives of Orofacial Sciences. 2006;1:9-14.

Daniel WW. Biostatistics: A Foundation for Analysis in the Health Sciences.

Edition. 7th ed. New York: John Wiley & Sons. 1999.

4. **Were the study subjects and setting described in detail?**

Certain diseases or conditions vary in prevalence across different geographic regions and populations (e.g. Women vs. Men, sociodemographic variables between countries). The study sample should be described in sufficient detail so that other researchers can determine if it is comparable to the population of interest to them.

5. **Was data analysis conducted with sufficient coverage of the identified sample?**

Coverage bias can occur when not all subgroups of the identified sample respond at the same rate. For instance, you may have a very high response rate overall for your study, but the response rate for a certain subgroup (i.e. older adults) may be quite low.

© 2024 Hall NY et al. *JAMA Network Open*.

6. Were valid methods used for the identification of the condition?

Here we are looking for measurement or classification bias. Many health problems are not easily diagnosed or defined and some measures may not be capable of including or excluding appropriate levels or stages of the health problem. If the outcomes were assessed based on existing definitions or diagnostic criteria, then the answer to this question is likely to be yes. If the outcomes were assessed using observer reported, or self-reported scales, the risk of over- or under-reporting is increased, and objectivity is compromised. Importantly, determine if the measurement tools used were validated instruments as this has a significant impact on outcome assessment validity.

7. Was the condition measured in a standard, reliable way for all participants?

Considerable judgment is required to determine the presence of some health outcomes. Having established the validity of the outcome measurement instrument (see item 6 of this scale), it is important to establish how the measurement was conducted. Were those involved in collecting data trained or educated in the use of the instrument/s? If there was more than one data collector, were they similar in terms of level of education, clinical or research experience, or level of responsibility in the piece of research being appraised? When there was more than one observer or collector, was there comparison of results from across the observers? Was the condition measured in the same way for all participants?

8. Was there appropriate statistical analysis?

Importantly, the numerator and denominator should be clearly reported, and percentages should be given with confidence intervals. The methods section should be detailed enough for reviewers to identify the analytical technique used and how specific variables were measured. Additionally, it is also important to assess the appropriateness of the analytical strategy in terms of the assumptions associated with the approach as differing methods of analysis are based on differing assumptions about the data and how it will respond.

9. Was the response rate adequate, and if not, was the low response rate managed appropriately?

A large number of dropouts, refusals or “not founds” amongst selected subjects may diminish a study’s validity, as can a low response rates for survey studies. The authors should clearly discuss the response rate and any reasons for non-response and compare persons in the study to those not in the study, particularly with regards to their socio-demographic characteristics. If reasons for non-response appear to be unrelated to the outcome measured and the characteristics of non-responders are comparable to those who do respond in the study (addressed in question 5, coverage bias), the researchers may be able to justify a more modest response rate.

© 2024 Hall NY et al. *JAMA Network Open*.

eTable 1. Data Extraction Table for All 90 Included Meta-Analysis Studies

Ref. no & Author	Country	Year of publication	Type of study	Origin of population/ sampling method	Population size (% women)	Age range Mean age (y)	Prevalence/ incidence type	Results	Quality score
1) Ackard et al (1)	United States	2003	Secondary analysis of cross-sectional study	1998 Minnesota survey	78685 (50.8% Female)	Grade 9-12	Point (last 12 months)	Diet pill<br>M: 2.3%, F:9.4%<br>Laxatives<br>M: 1.7%<br>F: 1.9%	7
2) Almuhlafi et al (2)	Saudi Arabia	2018	Cross-sectional	girls in 8 schools in northern Saudi Arabia in 2017-2018<br>Self-administered questionnaire<br>Random sampling	399 (100% female)	Mean=16.8	Point (ever used)	Laxatives- 5.3%	6
3) Amrock et al (3)	United States	2014	Secondary analysis of cross-sectional study	Data from YRB survey 2009-2011 in high school students<br>stratified, multistage sample design	≤18= 86.4% of 23286 (47.8% female)	Not reported	Point (past 30 days)	Diet pill<br>M = 10.5%<br>F = 8%	6

© 2024 Hall NY et al. *JAMA Network Open*.

4)Bae et al (4)	Korea	2023	Cross sectional survey	Data from the 15th Korean Youth Risk Behaviour web-based survey.<br>Multistage cluster sampling to obtain nationally representative sample of Korean students	N=18,159<br>F=60.4%	12-18 years	Point (past 30 days)	Diet pills<br>Total=16.5%<br>M=12.4%<br>F=19.2%<br>Laxatives/diuretics<br>Total=7.6%<br>M=8.2%<br>F=7.1%	9
5)Balluck et al (5)	Mauritius	2016	Cross-sectional	Convenient public places 2014-2015<br>Nutritional survey	200(52% female)	14-17	Point (last 12 months)	Laxatives<br>M & F: 0.5%	6

© 2024 Hall NY et al. *JAMA Network Open*.

6) Boutelle et al (6)	United States	2002	Secondary analysis of Cross-sectional	Data from The Voice of Connecticut Youth Survey (CYS) 1995-1996<br>Stratified random sampling<br>Grade 7, 9 & 11.	Non overweight= 6533(54.8% female)<br>Obese=594(36.7% female)<br>Overweight = 1203(37.6% female)	Not mentioned	Point (past week)	Diet pills<br>non-overweight: M:0.7%, F:3.4%<br>overweight: M: 1.2%, F: 4.1%<br>obese: M:1.8%, F:9.5%<br>laxatives<br>non-overweight: F= 1.3%, M= 1.3%<br>overweight: F=0.7%, M= 1.8%<br>obese: F= 3.2%, M= 2.0%	8

© 2024 Hall NY et al. *JAMA Network Open*.

7) Carter et al (7)	United Kingdom	2001	Cross-sectional	All girls in grade 9 in 6 girl's schools<br>Modified Self-report version of Eating Disorder Examination (EDE-Q)	808(100% female)	12-14 (mean=13.4)	Point (past 2 weeks)	Laxatives-9(1%)<br>Diuretics-4(0.4%)	5
8) Clayton et al (8)	Colombia	2017	Secondary analysis of cross-sectional study	Data combination from 2011 & 2013 cycles of Youth Risk Behavior survey (YRBS)<br>National representative of grade 9-12<br>Cluster sampling	28935(49.5% female)	Not mentioned	Point (past month)	Diet pills<br>M – 1.23%<br>F – 1.31%<br>Uses adjusted prevalence ratios	5
9) Colton et al (9)	Canada	2004	Case-control	Girls with type1 diabetes at Hospital Sick Children (HSC) & schoolgirls<br>Random sampling	Diabetic-101(100% female)<br>nondiabetic – 439(100% female), 303 used to give 3:1 matched sample with diabetics	9-14	Point (past 30 days)	Diet pill, laxatives, diuretics<br>Diabetic: 0%<br>Non-diabetic: 0%	9

© 2024 Hall NY et al. *JAMA Network Open*.

10) Cook et al (10)	Canada	2007	Cross-sectional survey	Females from four high schools in Nova Scotia participated in a self-report survey.<br>Convenience sample	1133 (female only)	15-19<br>M=16.75	Point (past 30 days)	Perceived as underweight(N=61)<br>Diet pills, powders, or liquids= 6.6%<br>Perceived as right weight(N=410)<br>Diet pills, powders, or liquids= 4.6%<br>Perceived as overweight(N=359)<br>Diet pills, powders, or liquids= 5.6%<br>Total= 3.8%	8
11) Croll et al (11)	United States	2002	Secondary analysis of cross-sectional study	Grade 9 & 12 who completed 1998 Minnesota Student Survey	80354(50.4% girls)	Not mentioned	Point (last 12 months)	Diet pills - M=2.3%<br>F=9.4%<br>Laxatives – M=1.7%<br>F=1.9%	7

© 2024 Hall NY et al. *JAMA Network Open*.

12) Crowther et al (12)	Two counties in north-eastern Ohio	1985	Cross-sectional	Self-administered questionnaire<br>9-12 grades of 4 schools in middle/upper middle-income cities, rural communities<br>Cluster sampling	363(100% girls)	Mean = 16	Point (frequency in a month)	Laxatives = 4.7%	8

© 2024 Hall NY et al. *JAMA Network Open*.

13) Demissie et al (13)	United States	2015	cross-sectional study	Youth Risk Behavior Survey 1999-2009<br>Grade 9-12<br>Cluster sample	Not mentioned<br>Ranging in size from 13,601 in 2001 to 16,410 in 2009	9th to 12th grade	Point (past 30 days)	Diet pills<br>1999 – M=6.9%<br>F= 13.7%<br>2001 - M=8.6%<br>F=15.6%<br>2003 – M=8.2%<br>F=13%<br>2005 – M=5.9%<br>F=9.6%<br>2007- M=6.0%<br>F=8.7%<br>2009 – M=5.1%<br>F=7.8%	6

© 2024 Hall NY et al. *JAMA Network Open*.

14) Fan et al (14)	China	2010	Cross-sectional	Self-administered questionnaire<br>Grade 7,8,10,11 in 56 schools of 7 cities in China<br>Random cluster sampling	3544 (57%, girls)	14-18<br>Mean = 15.6	Point (ever tried)	Diet pills: M -4.1%<br>F – 15.9%<br>Diet food or tea: M – 2.4%<br>F – 6.2%	7
15) Field et al (15)	United states	1999	Part of a cohort study	Growing Up Today Study<br>Children of women (gave children birth between 1982-1986) who responded recent Nurses' Health Study questionnaire<br>Several validated self-administered questionnaires to measure different aspects	16114(53% female)	9-14	Point (measured at several frequencies of a month)	Diet pills<br>M:0.1%<br>F: 0.7%<br>Laxatives<br>M: 0.3%<br>F: 0.3%	7

© 2024 Hall NY et al. *JAMA Network Open*.

16) Fournier et al (16)	United States	2009	Secondary analysis of cross-sectional study	Grade 9-12 students<br>Data from 2005 Massachusetts Youth Risk Behavior Survey (MYRBS)<br>Random sampling	Not homeless:3112 (49.5% female)<br>Homeless:152 (44.1% female)	Not Mentioned	Point (past 30 days)	Diet pill-<br>Not homeless<br>M & F=4.1%<br>Homeless<br>M & F=12.6%	6

© 2024 Hall NY et al. *JAMA Network Open*.

17) French et al (17)	United States	1998	Cross-sectional	Students in both survey & interview conducted in inner-city schools Minneapolis	43(76% female)	13-17<br>Mean-15	Point (past month and year)	Survey: M & F<br>Diet pill- 2.3% (past month), 11.6%)<br>Laxatives- 0% (past month), 2.3% (past year)<br>Diuretics- 0% (past month), 0% (past year)<br>Interview: M & F<br>Diet pill- 0% (past month), 7% (past year)<br>Laxatives- 0% (past month), 2.3% (past year)<br>Diuretics- 0% (past month), 0% (past year)	6

© 2024 Hall NY et al. *JAMA Network Open*.

18) French et al (18)	United States	1995	Cross-sectional	data as a part of longitudinal study, Female students of grade 9-11 in a suburban school district<br>Investigator administered questionnaire	1015(100% female)	grades 9 through 12	Point (past month)	Diet pills-5.4%<br>Laxatives-1.6%<br>Diuretics-1.0%	7
19) Ganesan et al (19)	India	2018	Cross-sectional	College girls in Coimbatore	1200(100% female)	18-19	Point (last year)	Laxatives-5.3%	6
20) Gordon et al (20)	United States	2020	Secondary analysis of cross-sectional study	Data from 2013 Massachusetts Youth Health Survey	5488 (53.4% female)	Mean=16	Point (last 30 days)	Diet pill<br>M: 4.5%<br>F: 6.8%<br>Total=5.7%	7
21) Grigg et al (21)	Australia	1996	Cross-sectional	32 government schools in on region of NSW, School girls<br>Random, two-stage cluster sampling	869(100% female)	14-18	Point (lifetime use)	Diet pill=5%<br>Diuretics=2%<br>Laxatives=5%	6

© 2024 Hall NY et al. *JAMA Network Open*.

22) Guarino et al (22)	Italy	2005	Cross-sectional	High school children in two mountain areas of Valsesia<br>Italian version of YRBSS	Total 2121<br>Less than 18<br>1037(48.8% female)	14-20	Pont (last 30 days)	Diet pill<br>M:1.5%, F:2.8%<br>Combined: 2.1%	5
23) Guss et al (23)	United States	2016	Secondary analysis of cross-sectional study	Data from 2013 Massachusetts Youth Health Survey<br>Random sampling	transgender<br>67(38.8% female)<br>Cisgender<br>2406 (53.6% female)	Less than 18	Point (last 30 days)	Diet pill<br>Transgender: 4.8%,<br>Cisgender: M: 1.0%, F: 3.1%<br>Laxatives<br>Transgender: 4.8%<br>Cisgender: M: 1.0%, F: 3.1%	7

© 2024 Hall NY et al. *JAMA Network Open*.

24) Hadland et al (24)	United States	2014	Secondary analysis of cross-sectional study	Data from four recent consecutive cycles of the MYRBS (2003-2009)	Total:12984<br>50.6% female	Median age:15	Point (during past 30 days)	Dieting pills<br>M: Exclusively heterosexual:4.2%<br>Heterosexual, but with current same sex partners:16.4%<br>gay:18.8%<br>bisexual:8.2%<br>unsure of sex orientation: 9.6%<br>F: Exclusively heterosexual:5.8%<br>Heterosexual, but with current same sex partners:8.4%<br>Lesbian:14.9%	9

© 2024 Hall NY et al. *JAMA Network Open*.

								Bisexual:15.8%<br>unsure of sex orientation: 6.7%	
25) Harrison et al (25)	Jamaica	2019	Cross-sectional	4 divisions 16 schools<br>EAT-26<br>Convenience sampling, stratified random sampling	521 (male = 44.6%)	mean age = 14.8	Point (last 30 days)	Taken laxatives, diuretics and diet pills<br>M: 2.0%, F:4.6%,<br>total =3.5%	7
26) Hazzard et al (26)	United States	2017	Secondary analysis of cross-sectional study	Data from Youth risk behavior survey, 1999-2013	31601(44.9% female)	Grade 9-12	Point (lifetime use)	Diet pills<br>M: 7.3%<br>F: 13.2%	7

© 2024 Hall NY et al. *JAMA Network Open*.

27) Hibbard et al (27)	United states	1990	Cross-sectional	Grade 7-12 students, 2 middle & 2 high schools in a single city (suburban-rural setting)<br>Self-administered health behaviour survey	3998 (51% girls)	Not mentioned	Point (lifetime use)	Laxatives<br>M & F (no abuse)=13%<br>M & F (sexual abuse) =19%<br>M & F (physical abuse) =18%<br>M&F (sexual and physical abuse) =35%	8
28) Hooper et al (28)	United States	2020	Cross-sectional	Adolescents from twenty public middle and high schools in the Minneapolis–St. Paul metropolitan area of Minnesota.	2285 (54.2% female)	Mean = 14.4 years	Point (Past year)	Diet pill- 2.2%<br>Laxatives- 1.1%<br>Diuretic- 1.0%	6

© 2024 Hall NY et al. *JAMA Network Open*.

29) Hoseini-Esfidarjani et al (29)	Iran	2020	Cross-sectional	Adolescent girls in last grade in high school<br>Multi-stage sampling, self-administered	491 (100% female)	Mean = 17.23	Point (lifetime use)	Diet pill-5.1%<br>Laxatives-5.5%<br>Diuretics=1.6%	7
30) Howe et al (30)	United states	2007	Cross-sectional study	Diabetes Eating Problem Survey	295(46.4 females)	11-20<br>M=14.9	Point (past year)	Diet pills<br>Male-0.7%<br>Female-2.2%<br>Total – 1.4%<br>Laxatives<br>Male-0%<br>Female-2.9%<br>Total – 1.4%<br>Diuretics<br>Male-0%<br>Female-0.7%<br>Total – 0.3%	6
31) Johnson et al (31)	United States	2016	Cross-sectional	Grade 9-12 students from 5 public high schools in 5 states using YRBS questionnaire<br>Convenience sampling	4148(51.7% Female)	Not mentioned	Point (past 30 days)	Diet pills-<br>M & F=5.3%	7

© 2024 Hall NY et al. *JAMA Network Open*.

32) Johnson et al (32)	Colombia	1992	Secondary analysis of cross-sectional study	Survey of selected psychiatric disorders 1985	2544(100% female)	13-18	Point (lifetime use)	Diuretics=0.9%<br>Diet pills (frequent use) = 5.1%<br>Laxative use (frequent) = 1.6%	4
33) Jones et al (33)	Canada	2001	Cross-sectional	School girls in 3 cities<br>3 questionnaires	1739(100% female)	12-18 (14.6)	Point (past month)	Diet pills-41(2.4%)<br>Laxatives-19(1.1%)<br>Diuretics-11(0.6%)	7
34) Jounghee et ala (34)	Korea	2016	Secondary analysis of cross-sectional study	National representative sample of middle & high school students<br>10th Youth Risk Behavior Web-based Survey<br>Multistage sampling, clustering, and stratification	20264(52.3% female)	12-18(16.43)	Point (past month)	Non-prescription pills- M & F= 2%<br>Laxatives/diuretics- M & F= 2.4%	7

© 2024 Hall NY et al. *JAMA Network Open*.

35) Killen et al (35)	United States	1986	Cross-sectional	Grade 10 in 4 northern California high schools<br>Self-administered questionnaire	1728(47.6% female)	15 years	Point (at least monthly)	Diet pill-M=3.8%<br>F=8.3%<br>Laxatives-M=5.8%<br>F=6.8%<br>Diuretics-M=2.4%<br>F=3.6%	5
36) Kim et al (36)	Korea	2018	Secondary analysis of cross-sectional study	Data from 11th Korea Youth Risk Behavior Web-based Survey in 2015<br>Stratified 3 stage cluster sampling	65529<br>(F- 48.4%)	12-18	Point (past 30 days)	Laxatives/diuretics-M = 0.7%<br>F = 0.9%<br>Diet pills<br>M= 0.6%<br>F = 1.2%	7

© 2024 Hall NY et al. *JAMA Network Open*.

37) Lawrence et al (37)	United States	2008	cohort	SEARCH for diabetes in Youth in 2001	Female=1165<br>≥19=12.6% (147)<br>Male=1521<br>≥19=11.9% (181)<br>Meeting age criteria<br>Female-1018<br>Male-1340	10-21<br>Mean=15	Point (lifetime use)	Diet pills<br>Within the age range<br>M:1.7%<br>F:8.4%<br>For the whole sample:<br>M=4.3%<br>F=9.5%	9
38) Leal et al (38)	Brazil	2020	Cross-sectional study	Cluster sampling	1156(48.88%)	Mean 16<br>12-19	Point-(past year (diet pills), last 3 months (diuretics and laxatives)	Diuretics (past 3 months)<br>Male-0.5%<br>Female-2.3%<br>Total-1.4%<br>Laxatives (past 3 months)<br>Male-0%<br>Female-0.7%<br>Total-0.3%<br>Diet pills (past year)<br>Male-0.9%<br>Female-3.6%<br>Total-2.2%	9

© 2024 Hall NY et al. *JAMA Network Open*.

39) Lee et al (39)	Korea	2019	Secondary analysis of cross-sectional study	Data from Korea Youth Risk Behavior Web-based Survey (KYRBWS) 2017	27284(60.4 % female)	13-18	Point (past 30 days)	Diet pill<br>M=1.5%<br>F=2.9%<br>Combined = 2.4%	5
40) Lim et al (40)	Korea	2014	Cross-sectional	Using national wide online panel survey<br>EAT-26	6943(49.9% Female)	12-18<br>Mean=15.4	Point (last 12 months)	Diet pills-M=0.7%<br>F=1.7%<br>Laxative or diuretic use<br>M=0.5%<br>F= 1.9%	5

© 2024 Hall NY et al. *JAMA Network Open*.

41) López-Guimerà et al (41)	United States & Spain	2013	Cross-sectional	School children 2009-2010<br>Investigator administered survey, drawn from Project EAT	Spain – 1501<br>52% females<br>US – 2793<br>53% female	Mean 14.3	Point (last year)	Spain<br>Laxatives-M=0.3%<br>F=0.7%<br>Diuretics-M=0.3%<br>F=1.8%<br>Diet pills-M=0.1%<br>F=1.1%<br>US<br>Laxatives-M=1.3%<br>F=1.2%<br>Diuretics-M=1.3%<br>F=1.3%<br>Diet pills-M=1.2%<br>F=2.6%	6

© 2024 Hall NY et al. *JAMA Network Open*.

42) Martinsen et al (42)	Norway	2010	Cross-sectional	Athletes & non-athletes of 16 Elite Sport High Schools & 2 high schools	Athletes-606(35.8% female)<br>Control-355(44.5% female)	15-16	Point (lifetime use)	Diet pill<br>Athletes=M: 0%, F:2.8%<br>Control=M:3.0%, F:7.0%<br>Diuretics<br>Athletes=M: 1.0%, F:1.4%<br>Control=M:3.6%, F:5.1%<br>Laxatives<br>Athletes=M: 0.8%, F:1.4%<br>Control=M:3.0%, F:5.7%	8
43) McGuire et al (43)	Nine English speaking Caribbean countries	2002	Cross-sectional	Public or private school were randomly selected from a written list<br>87-item questionnaire	15695 (61% female)	10-18(13.7+-2.1)	Point (lifetime use)	Laxatives-<br>M -10.9%<br>F-9.5%<br>Diuretics<br>M-3.3%<br>F-2.5%	7

© 2024 Hall NY et al. *JAMA Network Open*.

44) Neumark-Sztainer et al (44)	Israel	1995	Cross-sectional	Grade 10 girls from 3 public schools of Jerusalem, Israel	341(100% female)	Mean=15.3	Point (ever used)	Diet pills/dieuretics-5.9%<br>Laxatives/diuretics -5%	5
45) Neumark-Sztainer et al (45)	United States	1999	cross-sectional study	National representative sample of grade 7,9 & 11	9118(51.1% Female)	Not mentioned-	Point (past week)	Diet pill<br>M=1.2%<br>F=3.8%<br>Laxative or diuretics<br>M=1.6%<br>F=1.4%	5
46) Neumark-Sztainer et al (46)	United States	2000	Secondary analysis of cross-sectional study	Data from Olestra Post-Marketing Surveillance Study (OPMSS)<br>Random-digital-dial telephone survey	459(209 females - 45.5% girls)	M=14.6	Point (past month)	Diet pill or laxatives-<br>M= 0.8%<br>F= 1.9%	7

© 2024 Hall NY et al. *JAMA Network Open*.

47) Neumark-Sztainer et al (47)	United States	2002	Cross-sectional	Project EAT I (Eating among teens)<br>Urban and suburban schools I several districts<br>(response rate 81.5%)<br>Cluster sampling	4746 (49.7% female)	M = 14.9	Point (during last year/last week)	Diet pill: M=1.7%<br>F=6.6%<br>Laxatives: M=1.0%<br>F=1.8%<br>Diuretics: M=1.6%<br>F=1.7%<br>These behaviours relate to the last year	6

48) Neumark-Sztainer et al (48)	United States	2006	Cohort study – 5 years	Project EAT-II<br>Cluster sampling	N=407<br>(early & middle adolescents)<br><br>N =928<br>Middle & older adolescents	Younger group<br>T1 = 1999<br>Mean=12.8<br>T2 = 2004<br>Mean=17.2<br><br>Older group<br>T1=1999<br>M=15.8<br>T2=2004 mean=20.4	Point (past year in 1999 and 2004)	Diet pill: M & F<br>T1=3.5%, T2=14.2%<br>Laxatives<br>T1=1.1%, T2=2.6%<br>Diuretics<br>T1=1.3%, T2=1.3%<br><br>Diet pill<br>T1=7.5%<br>Laxatives<br>T1=1.8%<br>Diuretics<br>T1=2.1%	6
49) Neumark-Sztainer et al (49)	United States	2002	cross-sectional study	Adolescents with type1 Diabetes attended Diabetes Clinic at Children's Hospital in St. Paul, MN and completed AHEAD survey	143(49% female)	Mean=15.3 (2.3)	Point (past year)	Diet pill-<br>M=1.4%<br>F=2.9%<br>Laxatives-<br>M=0%<br>F=2.9%<br>Diuretics- M & F=0%	6

© 2024 Hall NY et al. *JAMA Network Open*.

50) Neumark-Sztainer et al (50)	United States	2011	Longitudinal study	Project EAT-II,1999-2010	2287 (55% girls)	Yourger group<br>Mean=12.8 (baseline)<br>23.2 (follow up)<br>Older group<br>Mean=15.9 (baseline)	Point (last year)	Diet pills, laxatives, diuretics<br>F: Early adolescence - 3.3%,1.4%,1.5%<br>Middle adolescence -6.5%, 1.3%, 1.6%<br>M: Early adolescence - 0.6%,0.4%,0.6%<br>Middle adolescence -1.1%,0.4%, 0.3%	7
51) Nishijima et al (51)	Japan	2019	Secondary analysis of cross-sectional study	Internet survey of high school students from regions all over Japan	1031(73.2% female)	15-18	Point (lifetime use)	Dietary products<br>M=1.2%<br>F=29.4%	5

© 2024 Hall NY et al. *JAMA Network Open*.

52) Nobakht et al (52)	Iran	2000	Secondary analysis of cross-sectional study	Second grade high school girls in Tehran during 1998<br>Translated version of EAT-26<br>Random sampling	3100(100% female)	15-18<br>Mean=16.11	Point (last 6 months)	Diet pills-2.13%<br>Laxatives/diuretics-2.51%	6
53) Nunes et al (53)	Brazil	2003	Cross-sectional	Totally visited 1524 houses<br>60 houses from each 26 census tracts<br>Trained assistants administered questionnaires.<br>Validated questionnaires used<br>Random sampling	513<br>118 within the age range	12-29<br>(12-15=23%<br>16-19=27.1%<br>20-29=49.9%)	Point<br>(last 3 months)	Laxatives=8.5%<br>Diuretics=2.8%<br>Diet pills – 5.1%	9

© 2024 Hall NY et al. *JAMA Network Open*.

54) Papas et al (54)	United States	2016	Secondary analysis of cross-sectional study	2011 US YRBS<br>Analysis between disability (20%) and no disability	N= 9775<br>Disability-1986<br>No disability-7789	≥18 = 15%	Point (last 30 days)	Diet pill<br>Adjusted for desired age<br>Disability-9.35%<br>No disability-3.4%<br>Without age adjustment<br>Diet pill<br>Disability = 11%<br>No disability=4%<br>Vomit/laxatives<br>Disability = 12%<br>No disability = 3%	8
55) Park et al (55)	Korea	2003	Cross-sectional	Healthy school children (grade 5,8 & 11)<br>self-administered	3382(49% female)	11-18	Point (lifetime use)	Diet pills-<br>M & F=0.62%	3

© 2024 Hall NY et al. *JAMA Network Open*.

56) Paxton et al (56)	Australia	1991	Cross-sectional	Seven schools in greater Melbourne area. Schools were selected to represent a range of geographic and socioeconomic status area	341 (61% female)	11 to 18 years (mean =14)	Point (past week)	Diet pills<br>M=1%<br>F=2%<br>T=1.6%<br>Laxative<br>M=1%<br>F=2%<br>T=1.6%<br>Diuretic<br>M=2%<br>F=1%<br>T=1.4%	6
57) Peducci et al (57)	Italy	2019	Cross-sectional	Regional Diabetes Centre of the University Children Hospital<br>Investigator based interview-Eating Disorder Examination (EDE)	85	8 – 14<br>Mean-13.4	Point (past month)	Diuretics: M & F=1.2%<br>Laxatives: M & F=5.9%	5

© 2024 Hall NY et al. *JAMA Network Open*.

58) Pernick et al (58)	United States	2006	Cross-sectional	Six schools in Sothern California<br><br>EDE-Q to suburban female high-school athletics	453(100% female)	13-18<br><br>15.7	Point (past month)	Laxative-1.5%<br><br>Diuretics-1.1%	5
59) Peveler et al (59)	United Kingdom	1992	Cross-sectional	IDDM patients from outpatient clinic<br><br>Non-diabetic subjects randomly from 2 general clinics<br><br>26-item EAT questionnaire was used	IDDM = 76(43.4% female)<br><br>Control= 76(43.4% female)	11-18<br><br>Mean:15.3	Point (past month)	Laxatives<br><br>IDDM = M & F:0%<br><br>Control = M & F:0%	6

© 2024 Hall NY et al. *JAMA Network Open*.

60) Phelps et al (60)	United States	1993	Cross-sectional	Middle and high school adolescent females surveyed in 1984,1989 & 1992<br>Self-administered questionnaire	1984<br>N=442<br>1989<br>N=395<br>1992<br>N=367	12-18	Point (lifetime use)	Diet pill<br>1984 = 88 (19.9%)<br>1989=41 (10.4%)<br>1992=37 (10.1%)<br>Laxatives<br>1984=19 (4.3%)<br>1989=12 (3.04%)<br>1992=10 (2.72%)	5
61) Pollina-Pocallet et al (61)	Spain	2021	Cross-sectional	High Schools and vocational schools of Lleida (Spain) between 2017 and 2019.<br>Survey	N=2496<br>F=51.2%	<19 years	Point (past year)	Laxatives – total: 0.92%<br>M: 0.99%<br>F: 0.86%<br>Diuretics – Total: 0.44%<br>M: 0.41%<br>F: 0.47%<br>Diet pills – total: 0.44%<br>M: 0.33%<br>F: 0.55%	6

© 2024 Hall NY et al. *JAMA Network Open*.

62) Rosen et al (62)	United States	1987	Cross-sectional	3 High schools in northern US<br>20-item self-report survey<br>Random sampling	1373(49.2% female)	Not mentioned	Point (past week)	Dieting pills (appetite suppressant includes both prescribed and non-prescribed)- M=1.8%, F=8.1%<br>Powder mix or food supp<br>M=4.5%<br>F=3.1%<br>Laxatives- M=0%<br>F=2.1%	6

© 2024 Hall NY et al. *JAMA Network Open*.

63) Rosenbaum et al (63)	United States	2009	Cross-sectional data	Youth Risk Behavior Survey (US,2000) 2 weeks test-retest reliability study<br>Convenience cluster sample of classes from 61 schools in urban, suburban and rural in 20	n=4619<br>F=53.4%	Age 13 to >18	Point (past month)	Diet pills<br>M & F:<br>Wave I- 7.8%<br>Wave 2 – 7.9%<br>Test-retest	6
64) Santos et al (64)	Brazil	2021	Cross sectional	Multilevel study in all public high schools in Olinda, Northeast Brazil.<br>Response rate of all invited students in the 27 schools was 37%	N=2424<br>F=55.9%	14-19 years	Point (past week)	Diet pills – 5.3%	6

© 2024 Hall NY et al. *JAMA Network Open*.

65) Serdula et al (65)	United States	1993	Secondary analysis of cross-sectional study	The Youth Risk Behavior Survey 1990 & Behavioral Risk Factor Surveillance System in 1989<br>Grade 9-12<br>Random sampling	11467	Less than 18	Point (past week)	Diet pill=<br>Male – 2%<br>Female- 4%	6
66) Senekal et al (66)	South Africa	2001	Cross-sectional	Female students registered for UNIN<br>Several self-administered questionnaires	180(100% female)<br>80 met the criteria	20±4.4<br>44.4%≤18	Point (past 6 months)	Diuretics- 2.3%<br>Diet pills- 10.4%<br>Laxatives- 15%<br>Results are for the whole sample	6
67) Silva et al (67)	Brazil	2018	Secondary analysis of cross-sectional study	Data from National Adolescent Student Health Survey 2015	10926 (49.7% female)	13-17	Point (last month)	Diet pills<br>M: 7.8%, F: 5.5%, total = 6.7%	6

© 2024 Hall NY et al. *JAMA Network Open*.

68) Stephen et al (68)	United States	2014	longitudinal study	DTrata files of survey Waves I, II, and III of the National Longitudinal Study of Adolescent Health (Add Health)<br>132 schools in US	Wave I- 20610<br>Wave II- 14618	Mean=15.66<br>Mean=16.22(1.64)	Point (past week)	Wave I<br>Laxative: M=0.08%<br>F=0.31%<br>Diet pill: M=0.17%<br>F=1.07%<br>Wave II<br>Laxative: M=0.04%<br>F=0.39%<br>Diet pill: M=0.21%<br>F=1.41%	7
69) Stigler et al (69)	India	2011	Cross-sectional	Grade 8 & 10 in 8 schools in Delhi	1818<br>(40% female)	Grade 8 mean=13.9<br>Grade 10 mean=15.8	Point (past year)	Diet pills<br>Overweight-15.1%<br>Underweight-10.7%<br>Food substitute<br>Overweight -11.6%<br>Underweight – 13.4%	5

© 2024 Hall NY et al. *JAMA Network Open*.

70) Story et al (70)	United States	1994	survey	Grade 7-12 American Indian-Alaska Native youth in 8 HIS service areas & 37 separate service units in 12 states<br><br>Revised version of Adolescent Health Survey	13454(50.3 % female)	12-18	Point (lifetime use and past month)	Diet pills – M=1.8%<br><br>F=5.1% (monthly)<br><br>Diuretics- M=0.8%<br><br>F=1.2% (ever used)<br><br>Laxatives – M=1.2%<br><br>F= 0.6% (ever used)	6
71) Sutter et al (71)	United States	2016	Secondary analysis of cross-sectional study	Data from 2013 Virginia Youth Survey	6903 (50.7% Female)	12-18	Point (past month)	Diet pill-<br><br>M & F=6.1%	7
72) Swanson et al (72)	United Kingdom	2014	Secondary analysis of cross-sectional study	Data from Avon Longitudinal Study of Parents and Children (ALSPAC)	7968(51.8% female)	14-16	Point (past year)	At Age 14<br><br>Laxatives- 0.43%<br><br>At Age 16<br><br>Laxatives- 1.88%	7
73) Stevens et al (73)	United States	1999	Cross-sectional	4th grade American Indian children in 8 schools<br><br>Convenience sampling	304	Not mentioned	Point (lifetime use)	Diet pill<br><br>M & F:0%<br><br>Laxatives<br><br>M & F:0%	6

© 2024 Hall NY et al. *JAMA Network Open*.

74) Stock et al (74)	Canada	2002	Secondary analysis of cross-sectional study	1999-2000 females met DSM-IV criteria for eating disorder<br>Self-administered questionnaire	77<br>Restrictors-60<br>Purgers-17	12-17<br>Mean=15.2	Point (past year)	Laxatives<br>Restrictors: M & F:14%<br>Purges: M & F: over 50% (exact percentage not reported)<br>Diuretics:<br>0% in both groups	7
75) Talamayan et al (75)	United States	2006	Secondary analysis of cross-sectional study	2003 Youth Risk Behavior Survey (YRBS)<br>Grade 9-12<br>3 stage cluster sampling	9714(51.5% female)	Not mentioned	Point (past month)	Diet pill=7.4%<br>M=4.6%<br>F=10%	7
76) Tăut et al (76)	Romania	2018	Cross-sectional study	2014 Romanian Health Behaviour in School-Aged Children study<br>150 schools in Romania – non random sample	5404 (50.6% female)	13- 15 years	Point (past-year)	Diet pills – 2%	6

© 2024 Hall NY et al. *JAMA Network Open*.

77) Thorlton et al (77)	United States	2012	Secondary analysis of cross-sectional study	Secondary analysis from 2007 National YRBS<br>195 schools' grade 9-12<br>Cluster sampling	14041<br>≥18=1866 (13.4%)<br>12175 met the criteria	<18	Point (past month)	Diet pills-<br>M & F=5.9%	6
78) Trigazis et al (78)	Canada	2004	Cross-sectional	Adolescence females in tertiary care pediatric eating disorder treatment center from May 1998-July 2000<br>Self-administered	46(100% female)	10-17<br>Mean = 15	Point (lifetime use)	Slimming tea- 13%	7
79) Tuffa et al (79)	Ethiopia	2020	Cross-sectional	20 Female high schools in 2017<br>Using self-administered questionnaire<br>Multistage random sampling	690 (100% female)	Mean=16.8	Point (past month)	Laxatives or diuretics=4 (0.6%)	4

© 2024 Hall NY et al. *JAMA Network Open*.

80) Tur-Sinai et al (80)	Israel	2020	Cross sectional	Data from the 2014 Israeli Health behavior in school-aged children study<br>Nationally representative sample of school aged children via random sampling of classrooms.	N=4390	11-17 years	Point (past year)	Diet pills or laxatives=3.5%	9
81) Utter et al (81)	New Zealand	2012	Secondary analysis of cross-sectional study	Data from Youth'07, a nationally representative survey<br>Secondary school students randomly selected through 2 stage cluster sampling	8690	13-18	Point (past year)	Diet pills<br>M=0.7%<br>F= 3.0%	7

© 2024 Hall NY et al. *JAMA Network Open*.

82) Ursoniu et al (82)	Romania	2011	Cross-sectional	May-June 2005, 48schools<br>Self-administered questionnaire<br>Random, stratified sampling	2152(50% female)	Grade 9-12	Point (past month)	Diet pills<br>M: 4.1%<br>F:8.9%<br>Total: 6.6%	7
83) Vervaet et al (83)	Belgium	2000	Cross-sectional	Girls of 5th year in 2 secondary schools, fashion models, Eating Disorder patients	School girls=333<br>Models=11<br>ED=32	School girls Mean=16.8<br>Model Mean=19<br>ED<br>Mean=24	Point (lifetime use)	Laxatives<br>School Girls = 15.9%<br>Diet pills<br>School Girls = 4.8%	5

© 2024 Hall NY et al. *JAMA Network Open*.

84) Vidot et al (84)	United States	2016	Cross-sectional	Data from YRBS 2013<br>Cluster sampling	5620 (67.4% female)	≥18 = 14.5%	Point (past month)	Diet pill<br>Marijuana: M & F: 35.5%<br>Alcohol: M & F: 58.5%<br>Cigarette: M & F: 27.7%<br>Laxatives or purging<br>Marijuana: 35.1%<br>Alcohol: 58.0%<br>Cigarette: 29.0%	7
85) Visser et al (85)	South Africa	2014	Cross-sectional	Grade 8-11 Traditional Jewish high school in Johannesburg<br>26-item Eating Attitudes Test (EAT-26) & modified section of USA Youth Risk Behavior Survey<br>Whole sample	220(100% female)	13-18<br>Mean=15.68	Point (past year)	Diet pills: 6.8%	5

© 2024 Hall NY et al. *JAMA Network Open*.

86) Wheaton et al (86)	United States	2013	Cross-sectional	National Youth Risk Behavior Survey 2007<br>Grade 9-12 High school students in 195 schools	N=12087 (50.6% female)	14-18	Point (past month)	Diet pill<br>M: 4.0%<br>F: 7.2%	6
87) Waaddegaard et al (87)	Denmark	2002	cross-sectional study	Grade 8-12 primary/secondary/high schools<br>Self-administered questionnaire<br>Random sampling	322 (50.6% female)	14-16	Point (lifetime use)	Laxatives<br>M- 0.6%, F- 0.9%<br>Dieting pills<br>M – 0.6%, F -4.8%	6

© 2024 Hall NY et al. *JAMA Network Open*.

88) Watson 2018 et al (88)	United States	2018	Secondary analysis of cross-sectional study	Data from 3 waves 1998,2004,2010 of Minnesota Student Survey (MSS)<br>Sexual partners	1998 = 18456<br>2004 = 17637<br>2010 = 19504<br>Total = 55,597	Mean-17(1.48)	Point (past year)	Diet pill<br>1998<br>Male= opposite-sex=3.3%<br>both-sex partner=12.2%<br>same-sex partner=9.5 %<br>Female=opposite-sex=16.8%<br>both-sex partner=27.8%<br>same-sex partner=8.7 %<br>2004<br>Male= opposite-sex=4.7%<br>both-sex partner=12.7%	7

© 2024 Hall NY et al. *JAMA Network Open*.

								same-sex partner=8.2 %<br>Female=opposite-sex=12.1%<br>both-sex partner=20.7%<br>same-sex partner=11.0%<br>2010<br>Male= opposite-sex=2.6%<br>both-sex partner=7.0 %<br>same-sex partner=7.5 %<br>Female=opposite-sex=6.5%<br>both-sex partner=13.9%	

© 2024 Hall NY et al. *JAMA Network Open*.

								same-sex partner=4.3 %	

© 2024 Hall NY et al. *JAMA Network Open*.

89) Watson et al (89)	United States	2017	Secondary analysis of cross-sectional data	Pooled data from the 1999 to 2013 Massachusetts Youth Risk Behavior Surveys (N5 26,002)<br>Massachusetts public schools,57 to 75 schools in grade 9-12<br>Probability proportional sampling	Sample ranged from 2721(2009)-4415(1999)<br>N=26002	12-18<br>Mean=16.04	Point (past month)	1999/2001<br>M-Heterosexual=4.5%<br>Bisexual=12.5%<br>Gay=17.0%<br>F-Heterosexual=10.0%<br>Bisexual=14.6%<br>Lesbian=10.7%<br>2003/2005<br>M-Heterosexual=4.5%<br>Bisexual=10.4%<br>Gay=11.8%<br>F-Heterosexual=6.7%<br>Bisexual=16.9%<br>Lesbian =19.2%	6

© 2024 Hall NY et al. *JAMA Network Open*.

								2007/2009<br>M-Heterosexual=4.4%<br>Bisexual=6.6%<br>Gay=22.8%<br>F-Heterosexual=5.1%<br>Bisexual=15.2%<br>Lesbian=13.0%%<br>2011/2013<br>M-Heterosexual=2.5%<br>Bisexual=5.4%<br>Gay=13.1%<br>F-Heterosexual=3.9%<br>Bisexual=7.7%<br>Lesbian=20.6%	

© 2024 Hall NY et al. *JAMA Network Open*.

90) Zhang et al (90)	China and United States	2011	Cross-sectional	Representative sample of students in Hong Kong, Macau, Taipei, New York & Los Angeles<br>Cities were randomly with probability proportional to size sampling except Macau<br>High schools from two stage cluster sampling<br>Grade 9-12	Hong Kong<br>N=1478(55.7% female)<br>Macau<br>N=2596(61.9% female)<br>Taipei<br>N=2398(45.3% female)<br>New York City<br>N=7346(51.1% female)<br>Los Angeles<br>N=1061(51.9% female)	Hong Kong<br>Mean=16.1<br>Macau<br>Mean=16.5<br>Taipei<br>Mean=16.1<br>New York City<br>Mean=15.9<br>Los Angeles<br>Mean=15.9	Point (past month)	Diet pills<br>Hong Kong<br>M:0.9%, F:3.3%<br>Macau<br>M:1.0%, F:5.0%<br>Taipei<br>M:1.0%, F:2.8%<br>New York City<br>M:3.2%, F:5.6%<br>Los Angeles<br>M:6.3%, F:9.4%	7

© 2024 Hall NY et al. *JAMA Network Open*.

Appendix 4

eFigure 1. Weight Loss Product Use Among Adolescents

© 2024 Hall NY et al. *JAMA Network Open*.

Weight loss product use prevalence among adolescents

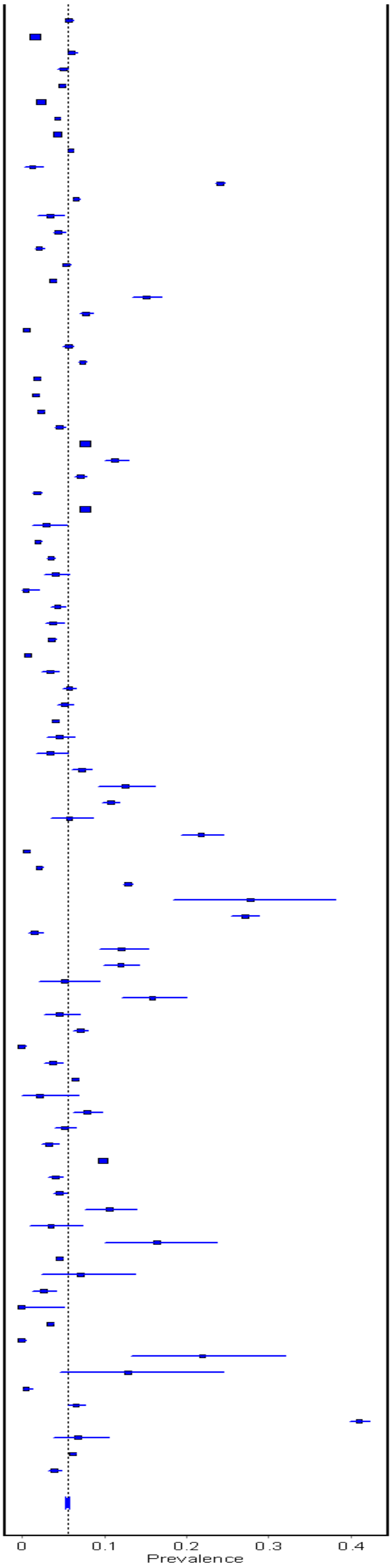

Study	Prev (95% CI)	% Weight
Hadland 2014	0.057 (0.053, 0.061)	1.6
Kim 2018	0.017 (0.016, 0.018)	9.3
Sutter 2016	0.061 (0.055, 0.067)	1.0
Johnson 2016	0.050 (0.044, 0.057)	0.6
Amrock 2014	0.049 (0.047, 0.052)	3.3
Lee 2019	0.024 (0.022, 0.026)	3.9
Jounghee 2016	0.044 (0.041, 0.047)	2.9
Clayton 2017	0.043 (0.041, 0.045)	4.1
Thorlton 2012	0.059 (0.055, 0.063)	1.7
Neumark-Sztainer 2000	0.013 (0.004, 0.026)	0.1
Bae 2023	0.241 (0.235, 0.247)	2.6
Silva 2018	0.067 (0.062, 0.071)	1.6
Harrison 2019	0.035 (0.020, 0.052)	0.1
Fournier 2010	0.045 (0.038, 0.052)	0.5
Guarino 2005	0.021 (0.015, 0.028)	0.3
Wheaton 2013	0.055 (0.051, 0.059)	1.7
Zhang 2011	0.039 (0.036, 0.042)	2.1
Killen 1986	0.152 (0.135, 0.169)	0.2
Rosenbaum 2009	0.078 (0.070, 0.086)	0.6
Field 1999	0.007 (0.006, 0.009)	2.3
Gordon 2020	0.056 (0.051, 0.063)	0.8
Talamayan 2006	0.074 (0.069, 0.079)	1.4
Swanson 2014	0.019 (0.015, 0.023)	0.7
Utter 2012	0.018 (0.015, 0.021)	1.2
Lim 2014	0.024 (0.020, 0.028)	1.0
López-Guimerà 2013	0.046 (0.040, 0.053)	0.6
Croll 2002	0.077 (0.075, 0.079)	11.4
Stigler 2011	0.114 (0.100, 0.129)	0.3
Neumark-Sztainer 2002	0.072 (0.065, 0.079)	0.7
Pollina-pocallet 2021	0.018 (0.013, 0.024)	0.4
Ackard 2003	0.077 (0.075, 0.079)	11.2
Howe 2007	0.031 (0.014, 0.054)	0.0
T□ut 2018	0.020 (0.016, 0.024)	0.8
Tur-sinai 2020	0.035 (0.030, 0.041)	0.6
Neumark-Sztainer 2011	0.041 (0.028, 0.058)	0.1
Balluck 2016	0.005 (0.000, 0.021)	0.0
Hooper 2020	0.044 (0.036, 0.053)	0.3
Leal 2020	0.039 (0.028, 0.051)	0.2
Boutelle 2002	0.038 (0.034, 0.042)	1.2
Stephen 2014	0.008 (0.007, 0.009)	2.9
Rosen 1987	0.034 (0.025, 0.045)	0.2
Serdula 1993	0.058 (0.051, 0.067)	0.5
Santos 2021	0.053 (0.044, 0.062)	0.3
Neumark-Sztainer 1999	0.041 (0.037, 0.045)	1.3
Paxton 1991	0.046 (0.030, 0.065)	0.1
Waaddegaard 2002	0.034 (0.017, 0.057)	0.0
Neumark-Sztainer 2006-older cohort 1999	0.072 (0.060, 0.085)	0.2
Phelps 1993-data 1992	0.125 (0.093, 0.161)	0.1
Fan 2010	0.108 (0.098, 0.119)	0.5
Neumark-Sztainer 1995	0.059 (0.036, 0.086)	0.0
Nishijima 2019	0.219 (0.194, 0.244)	0.1
Park 2003	0.006 (0.004, 0.009)	0.5
Hibbard 1990	0.021 (0.017, 0.026)	0.6
McGuire 2002	0.129 (0.124, 0.135)	2.2
Senekal 2001	0.277 (0.184, 0.381)	0.0
Johnson 1992	0.271 (0.254, 0.288)	0.4
Carter 2001	0.016 (0.008, 0.026)	0.1
Hoseini-Esfidarjani 2020	0.122 (0.095, 0.153)	0.1
Grigg 1996	0.120 (0.099, 0.142)	0.1
Almuhlafi 2018	0.053 (0.021, 0.095)	0.0
Vervaet 2000	0.159 (0.122, 0.200)	0.0
Crowther 1985	0.047 (0.027, 0.071)	0.1
Nobakht 2000	0.072 (0.063, 0.081)	0.4
Colton 2004	0.000 (0.000, 0.006)	0.0
Cook 2007	0.038 (0.028, 0.050)	0.2
Demissie 2015	0.065 (0.061, 0.068)	2.3
French 1998	0.023 (0.000, 0.068)	0.0
French1995	0.080 (0.064, 0.097)	0.1
Ganesan 2018	0.053 (0.041, 0.066)	0.2
Guss 2016	0.034 (0.024, 0.045)	0.2
Hazzard 2017	0.099 (0.096, 0.103)	4.5
Jones 2001	0.041 (0.032, 0.051)	0.2
Lawrence 2008	0.046 (0.038, 0.055)	0.3
Martinsen 2010	0.107 (0.077, 0.140)	0.1
Neumark-Sztainer 2002a	0.036 (0.010, 0.073)	0.0
Nunes 2003	0.164 (0.102, 0.237)	0.0
Papas 2016	0.046 (0.042, 0.050)	1.4
Peducci 2019	0.071 (0.024, 0.137)	0.0
Pernick 2006	0.026 (0.013, 0.043)	0.1
Peveler 1992	0.000 (0.000, 0.052)	0.0
Story 1994	0.035 (0.032, 0.038)	1.9
Stevens 1999	0.000 (0.000, 0.006)	0.0
Stock 2002	0.219 (0.133, 0.320)	0.0
Trigazis 2004	0.130 (0.046, 0.245)	0.0
Tuffa 2020	0.006 (0.001, 0.013)	0.1
Ursoniu 2011	0.066 (0.056, 0.077)	0.3
Vidot 2016	0.410 (0.397, 0.423)	0.8
Visser 2014	0.068 (0.038, 0.106)	0.0
Watson 2018	0.062 (0.059, 0.066)	2.8
Watson 2017	0.039 (0.032, 0.047)	0.3
Overall	0.055 (0.055, 0.056)	100.0

Q=24914.40, p=0.00, I2=100%

© 2024 Hall NY et al. *JAMA Network Open*.

Appendix 5

eFigure 2. Doi Plot and LFK Index for Publication Bias for All 90 Studies

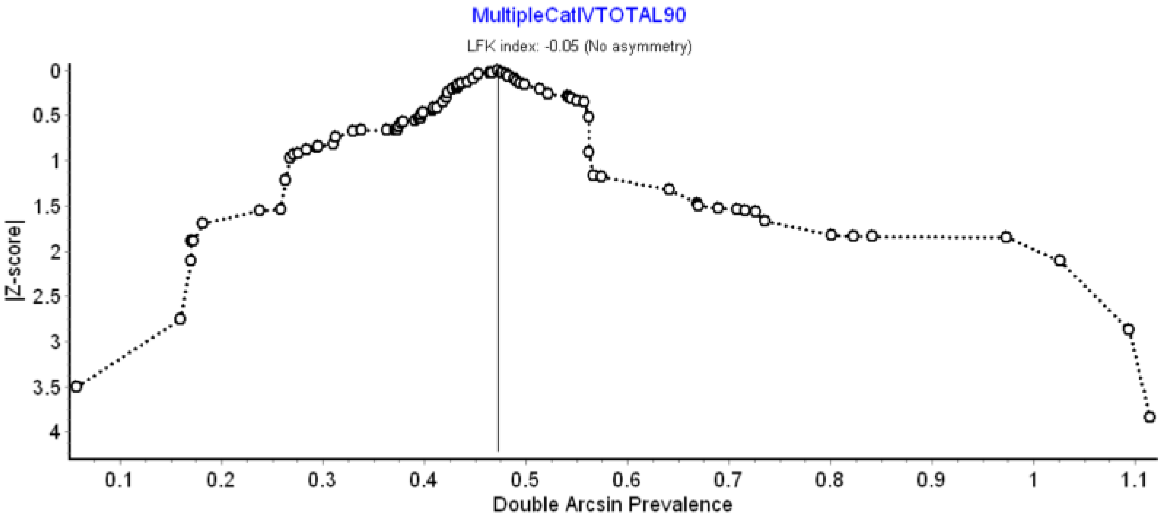

Appendix 6

eFigure 3. Doi Plot and LFK Index for Publication Bias for Weight Loss Product Use in the Past Week

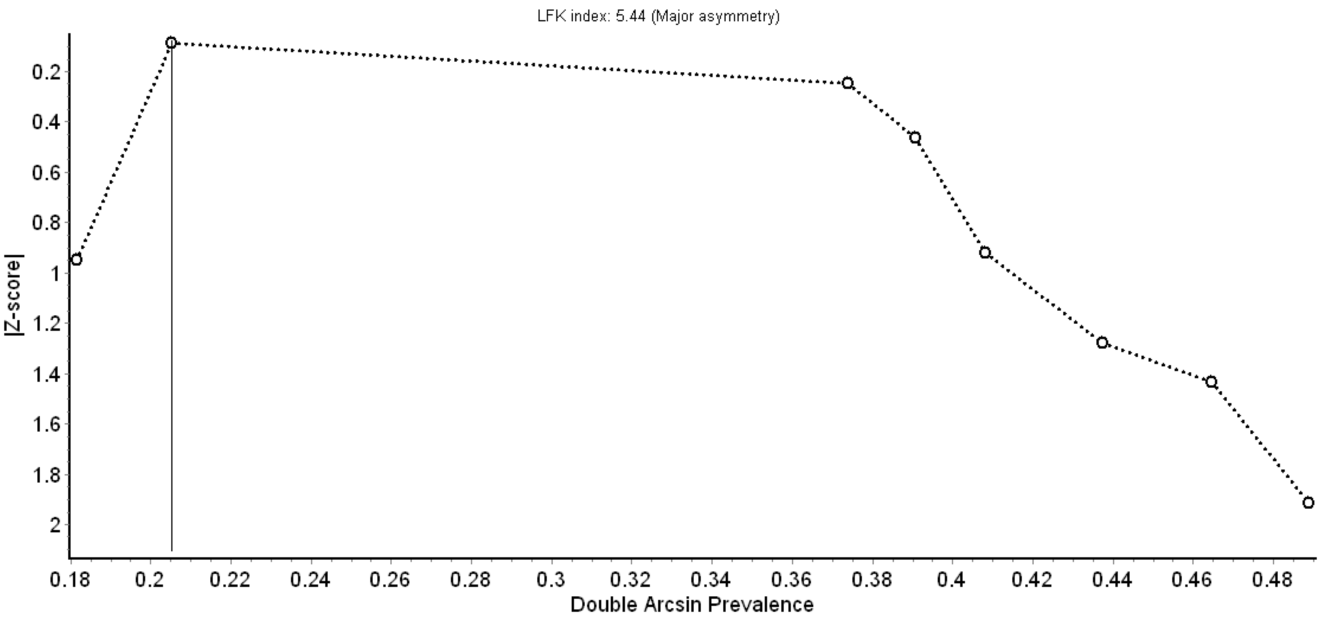

Appendix 7

eFigure 4. Doi Plot and LFK Index for Publication Bias for Weight Loss Product Use in the Past Month

© 2024 Hall NY et al. *JAMA Network Open*.

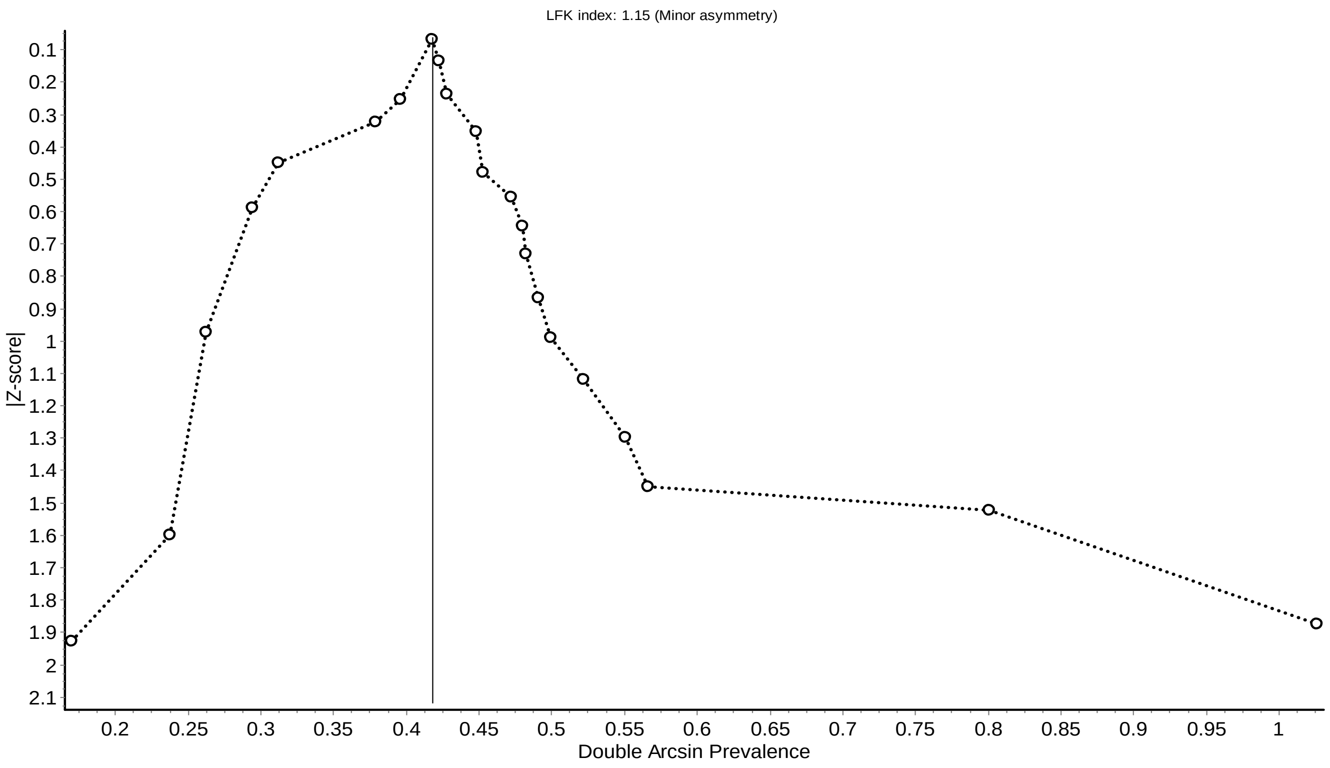

Appendix 8

eFigure 5. Doi Plot and LFK Index for Publication Bias for Weight Loss Product Use in the Past Year

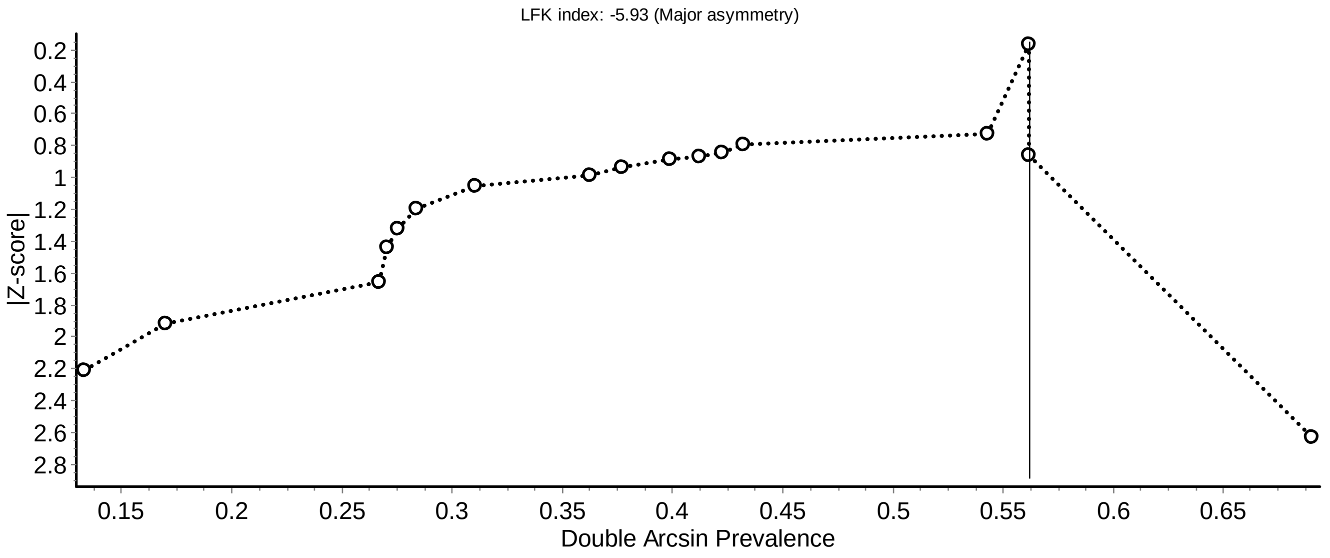

Appendix 9

eFigure 6. Doi Plot and LFK Index for Publication Bias for Weight Loss Product Use in the Lifetime

© 2024 Hall NY et al. *JAMA Network Open*.

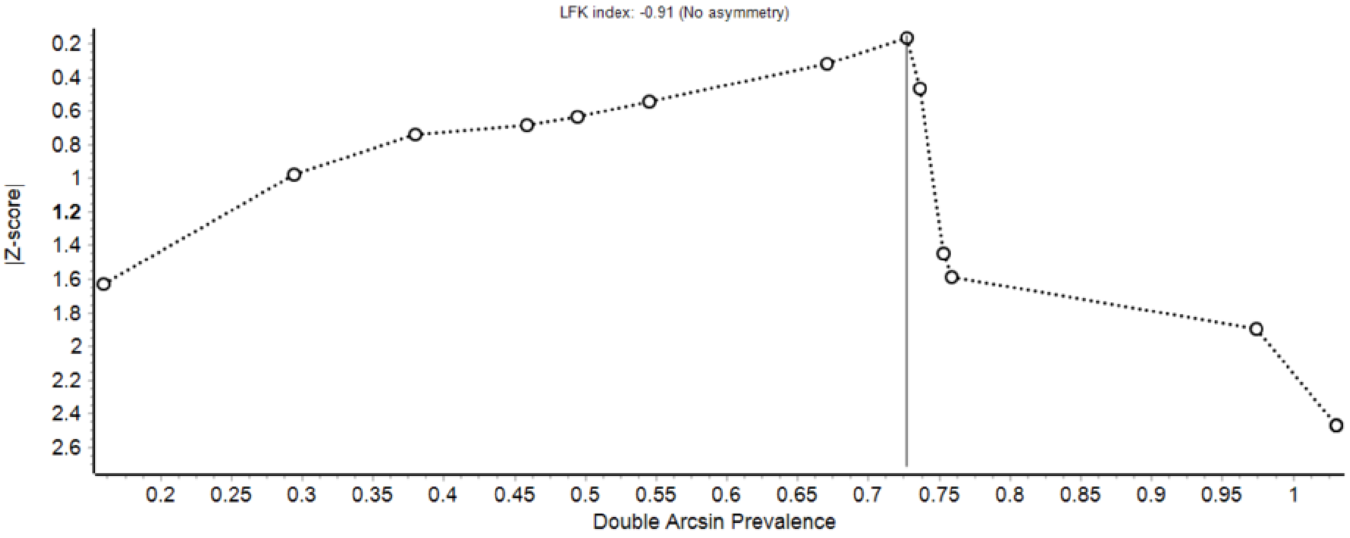

Appendix 10

eFigure 7. Meta-Analysis Results for Gender Differences in Weight Loss Product Use

Females – past week all products

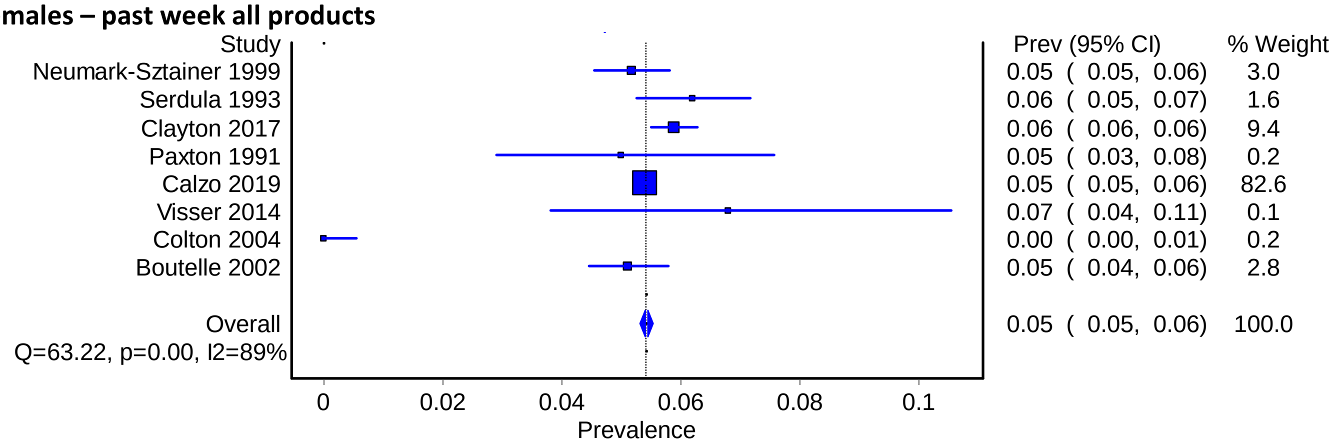

Females – past 30 days all products

© 2024 Hall NY et al. *JAMA Network Open*.

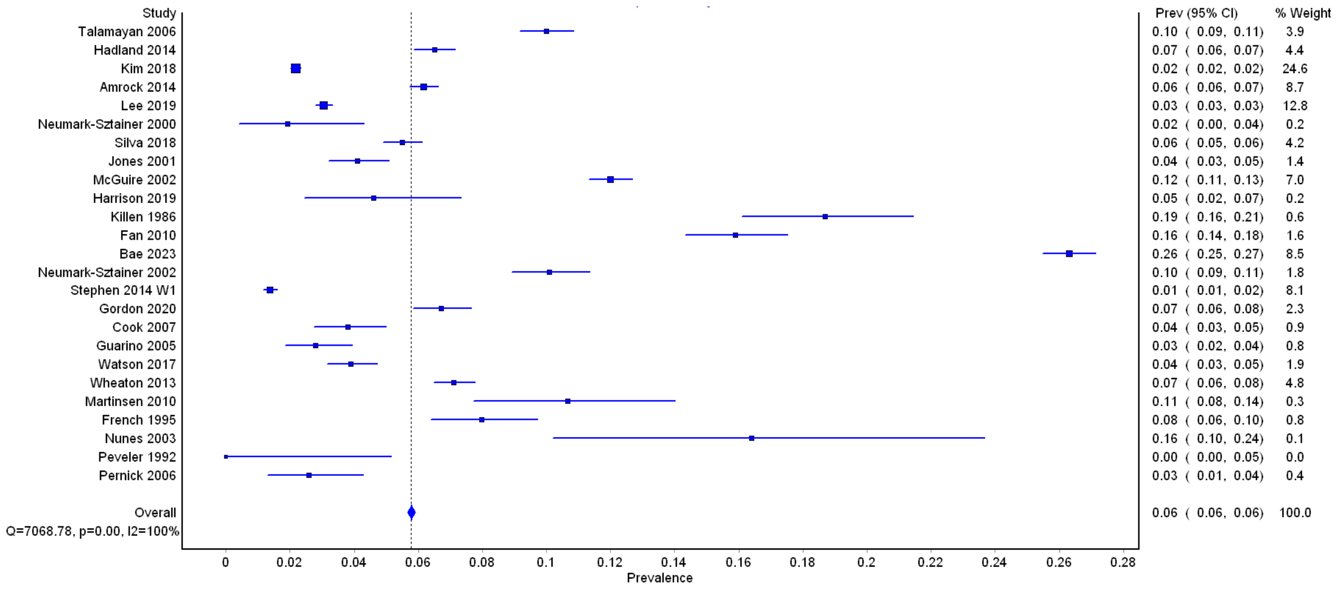

Females – past year all products

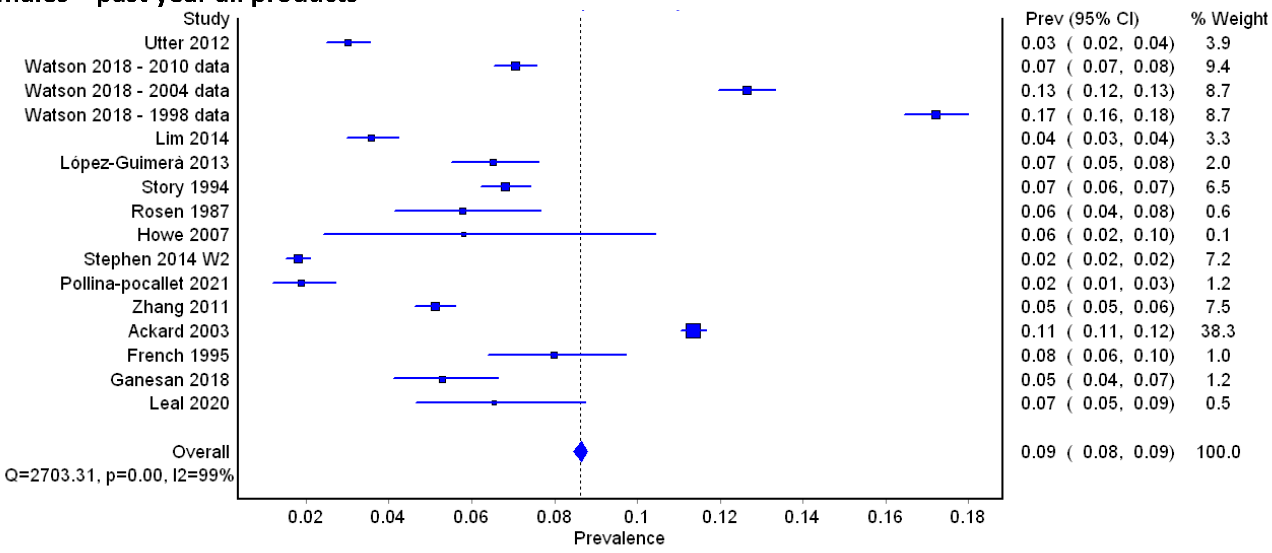

Females – lifetime use all products

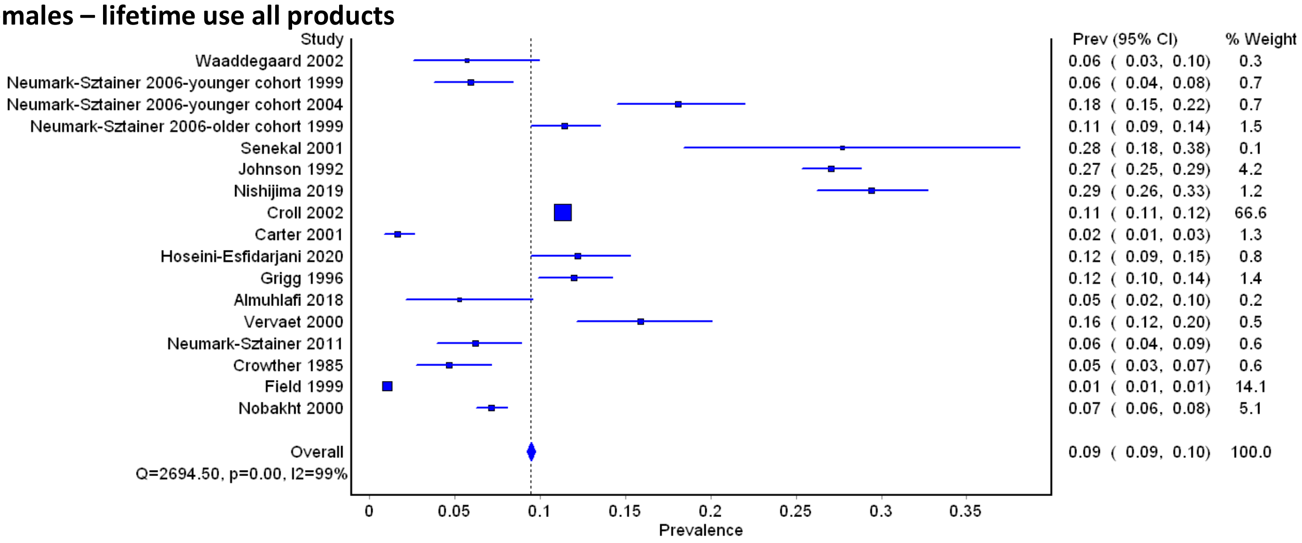

© 2024 Hall NY et al. *JAMA Network Open*.

Females – past week diet pills

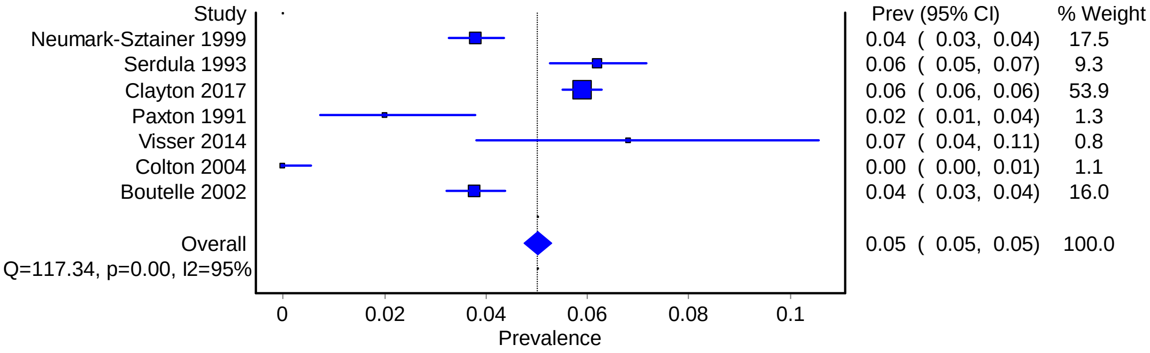

Females – past 30 days diet pills

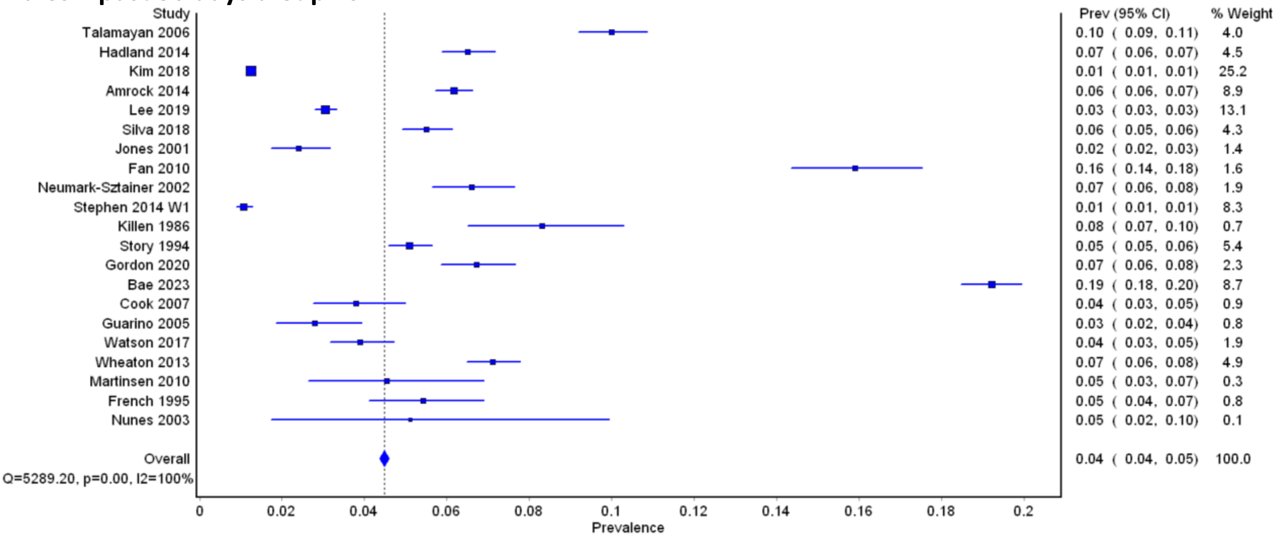

Females – past year diet pills

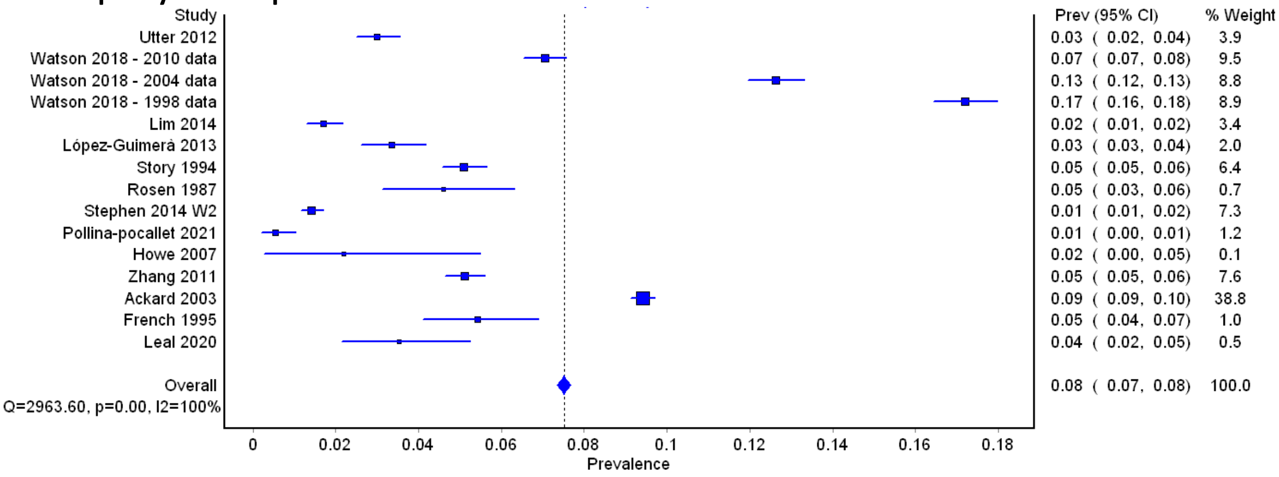

Females – lifetime diet pills

© 2024 Hall NY et al. *JAMA Network Open*.

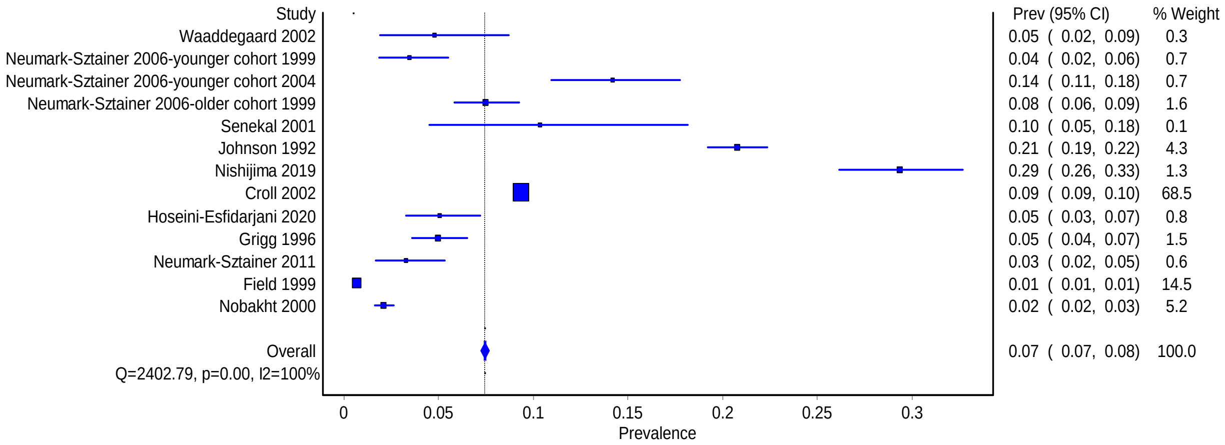

Females – past week diuretic use

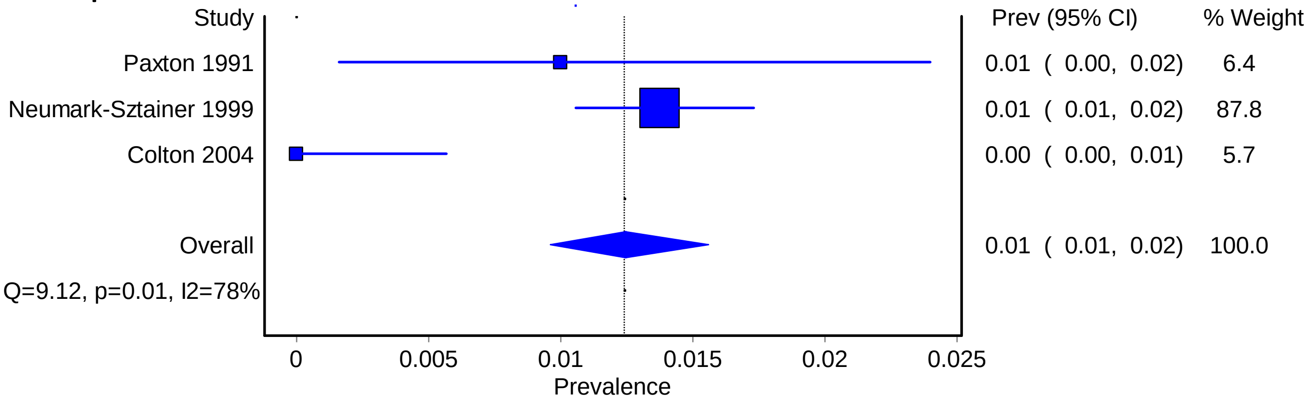

Females – past 30 days diuretic use

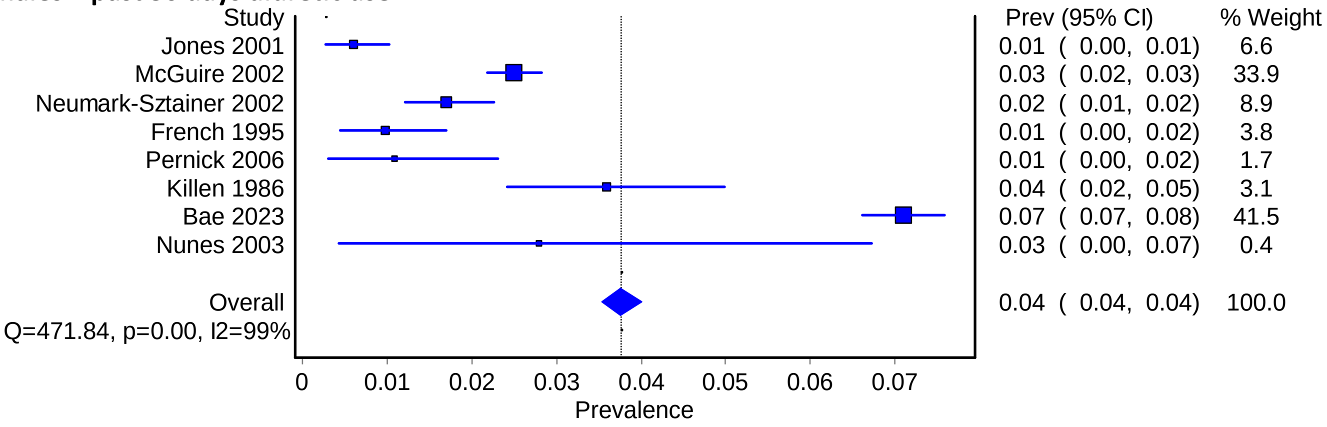

Females – past year diuretic use

© 2024 Hall NY et al. *JAMA Network Open*.

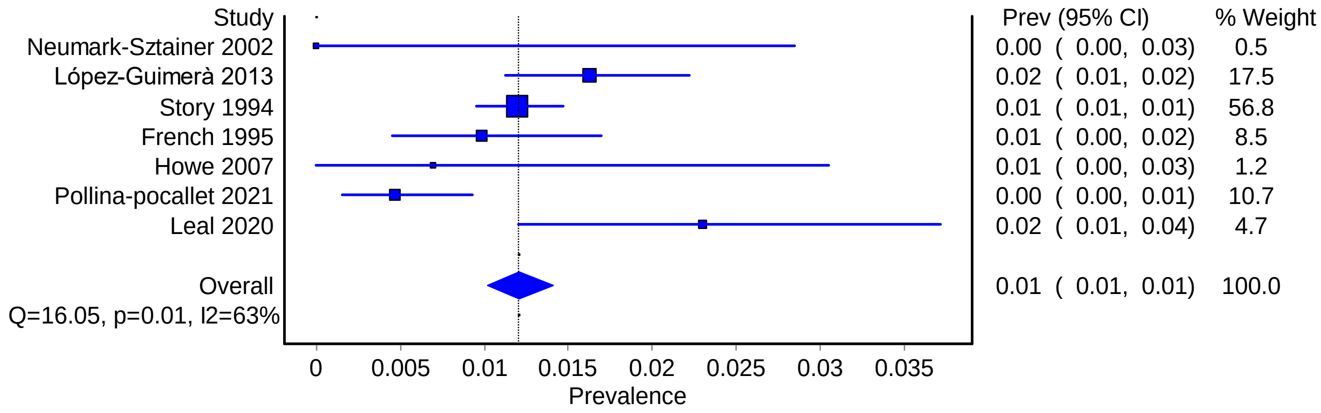

Females – lifetime use diuretic use

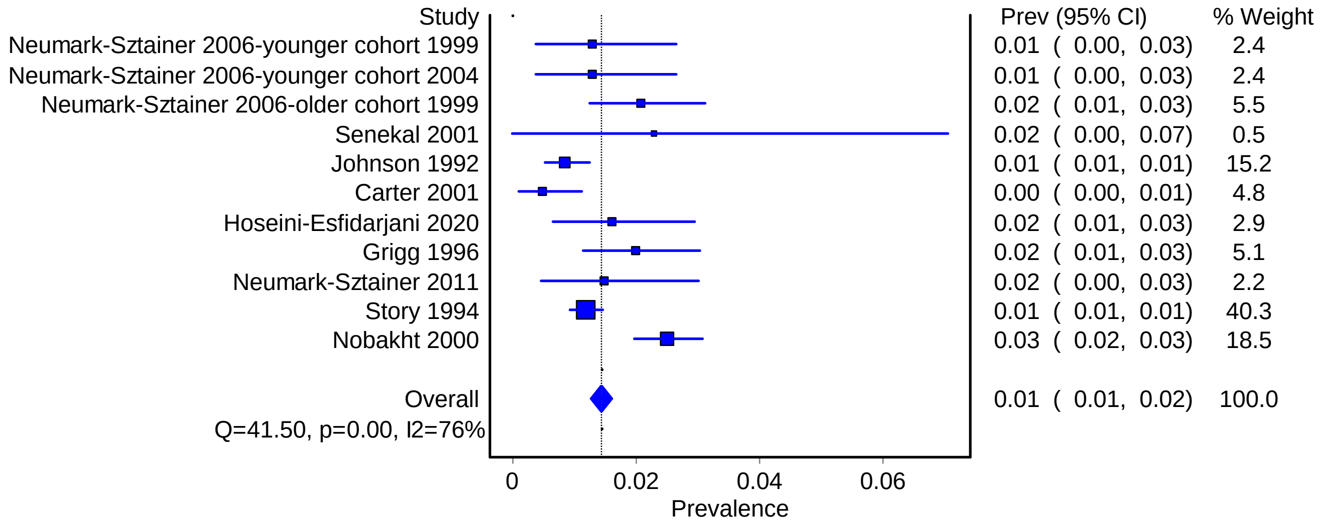

Females – past week laxative use

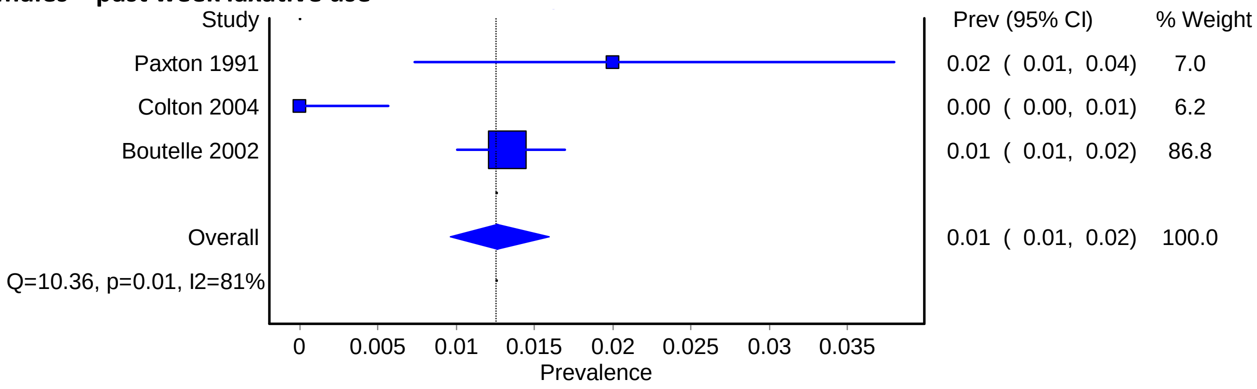

Females – past 30 days laxative use

© 2024 Hall NY et al. *JAMA Network Open*.

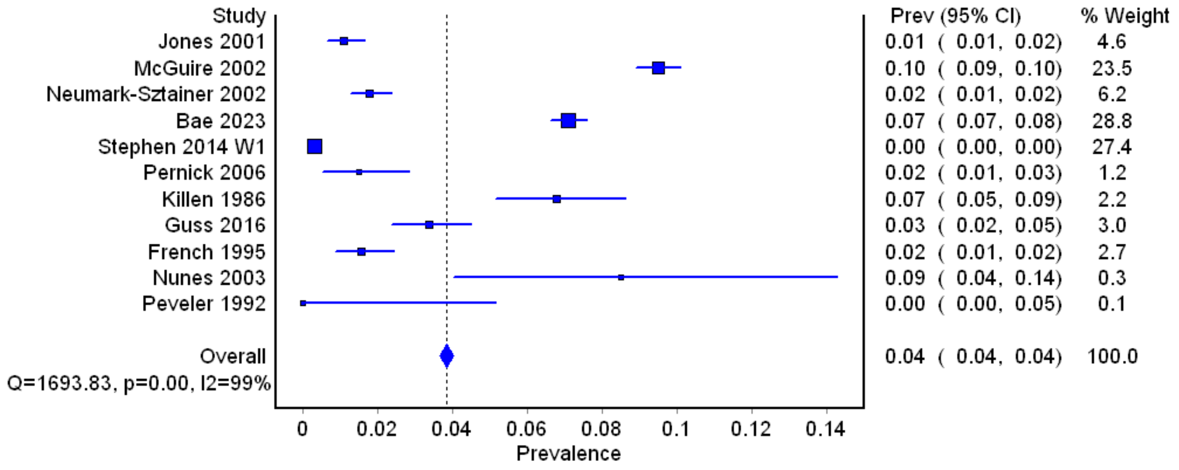

Females – past year laxative use

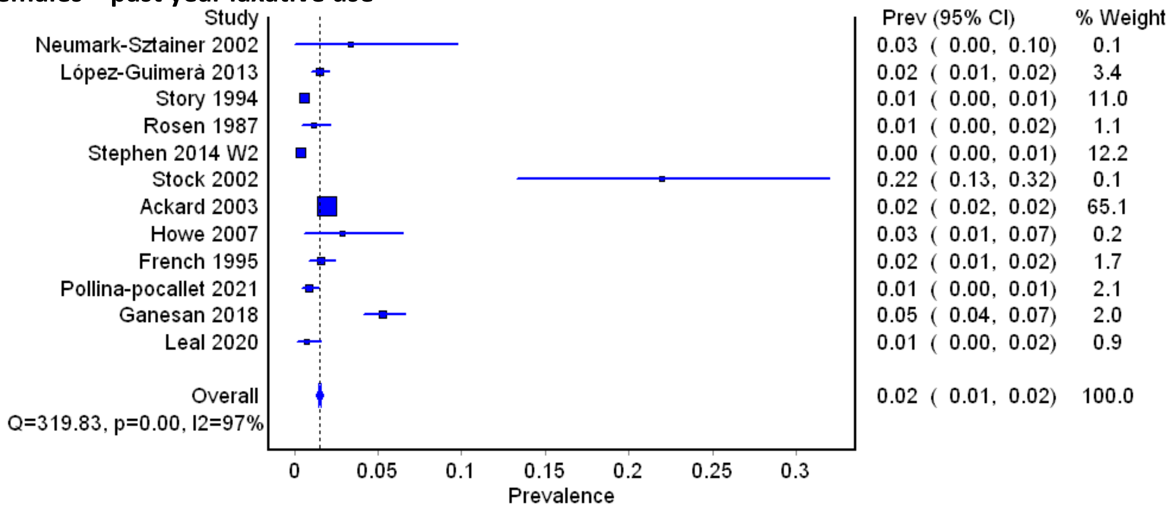

Females – lifetime use laxative use

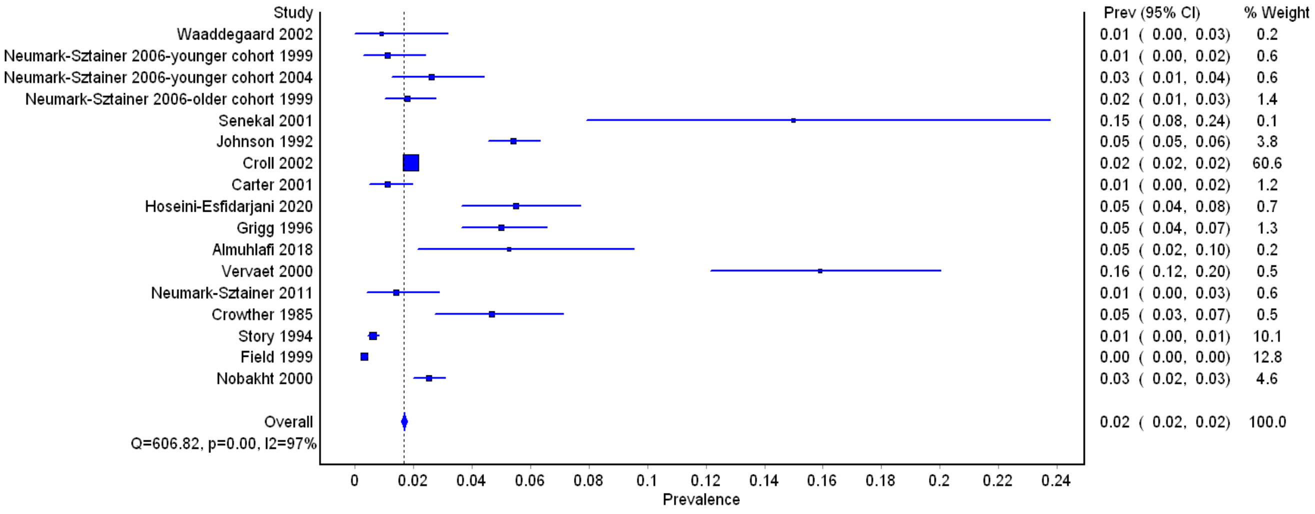

Males – past week use all products

© 2024 Hall NY et al. *JAMA Network Open*.

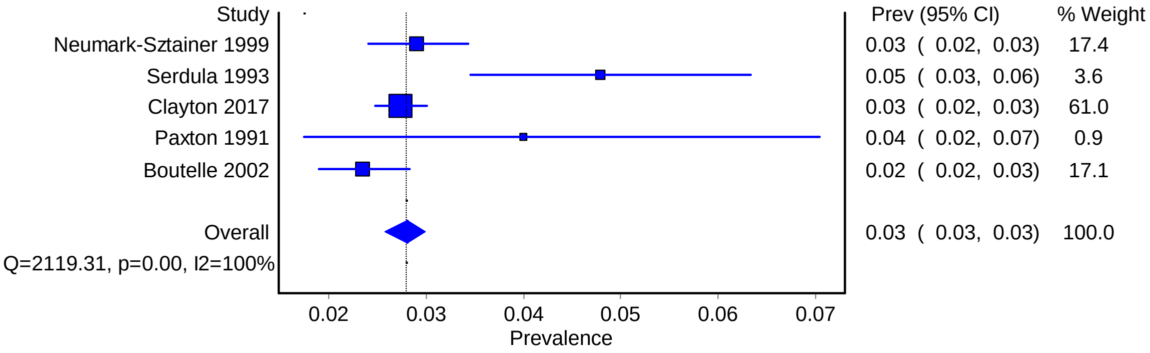

Males – past 30 day use all products

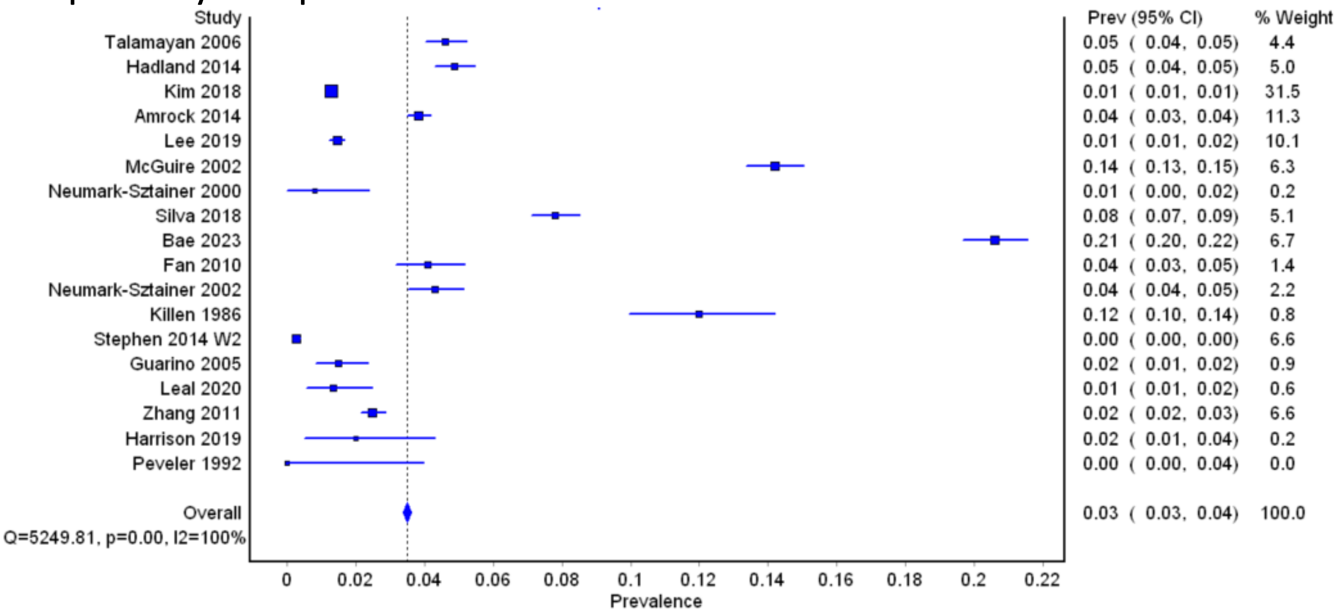

Males – past year use all products

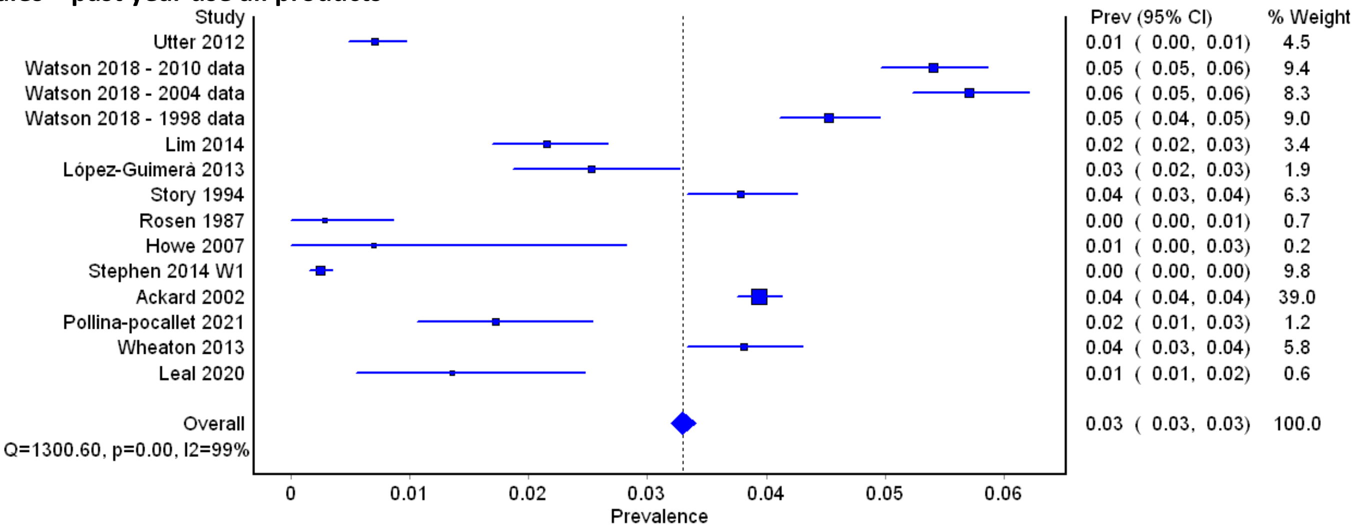

Males – lifetime use all products

© 2024 Hall NY et al. *JAMA Network Open*.

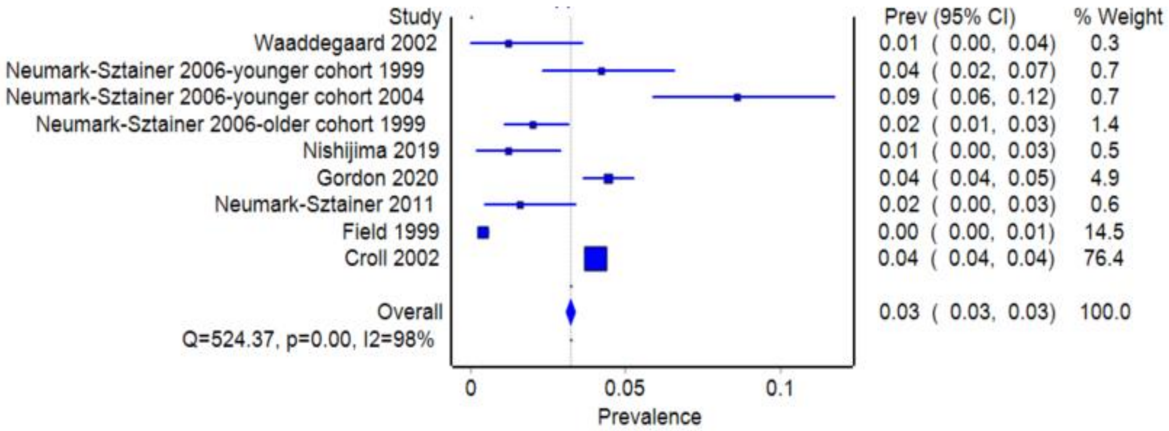

Males – past week diet pills

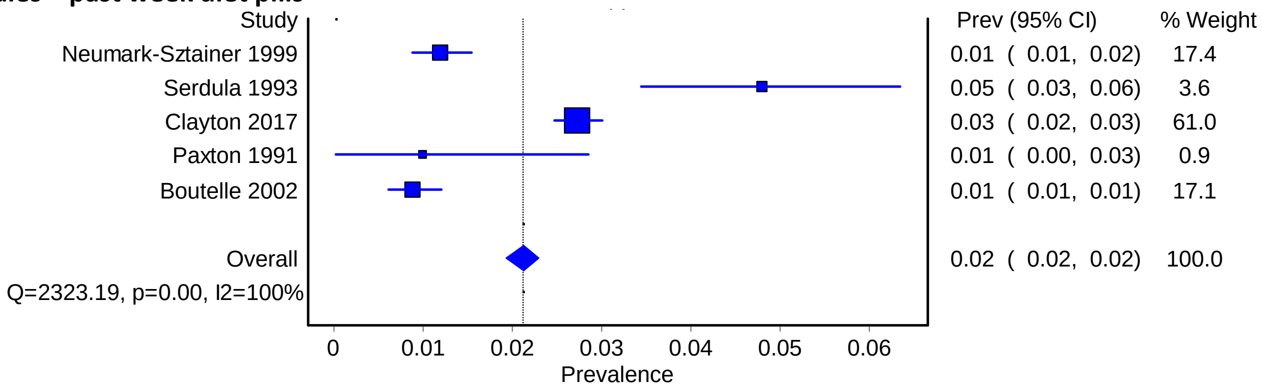

Males – past month diet pills

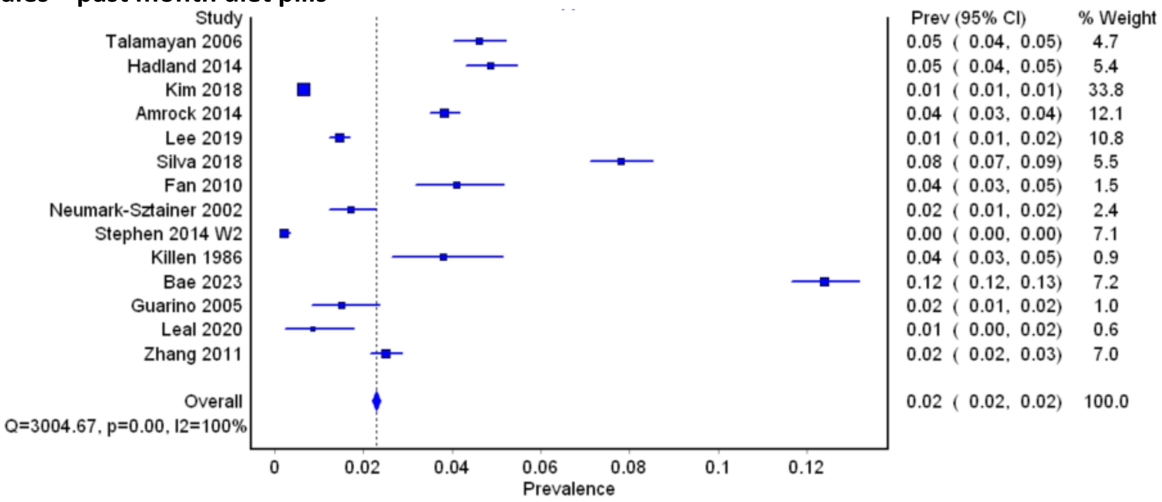

Males – past year use diet pills

© 2024 Hall NY et al. *JAMA Network Open*.

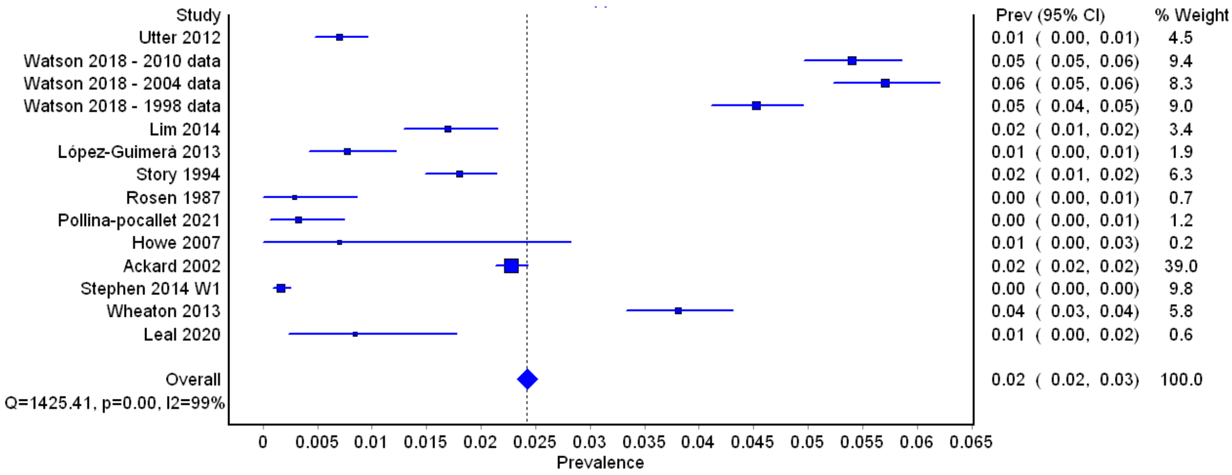

Males – lifetime diet pills

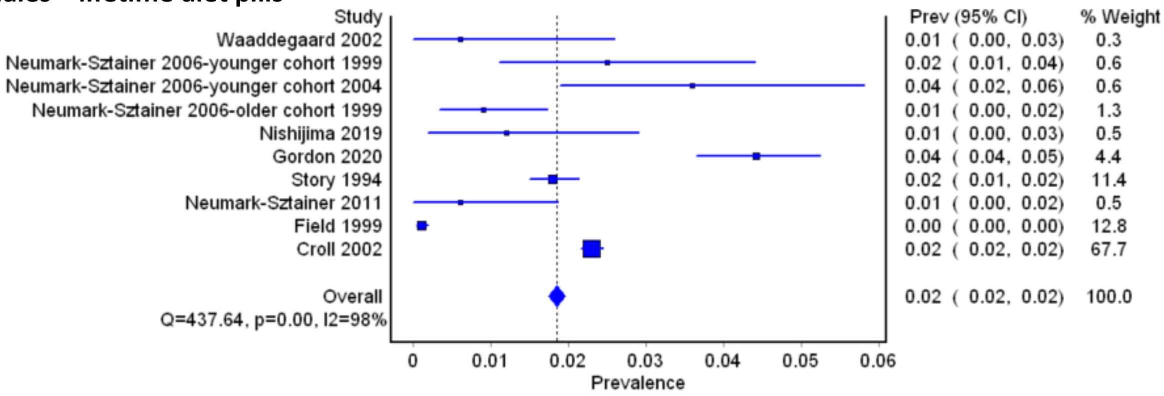

Males – past week laxative

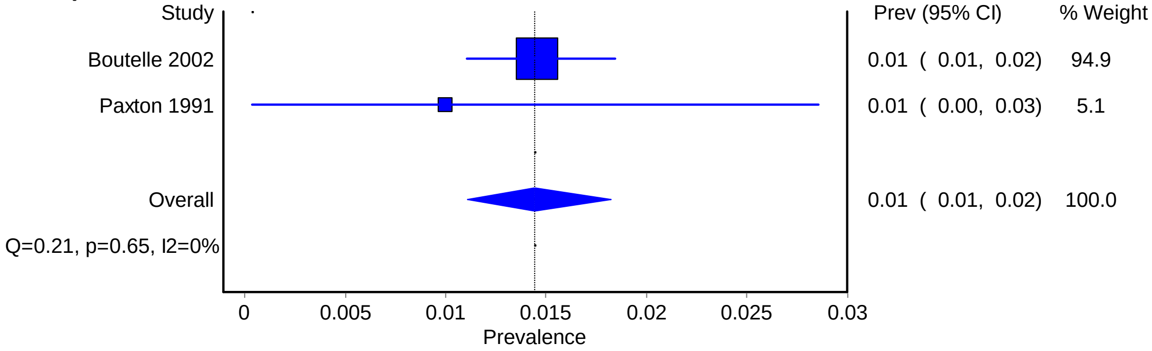

Males – past 30 day use laxative

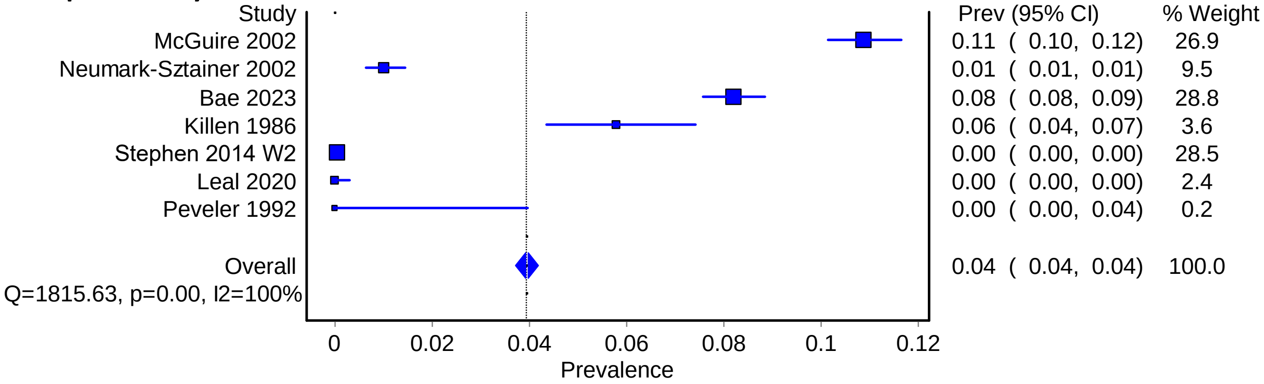

© 2024 Hall NY et al. *JAMA Network Open*.

Males – past year use laxative

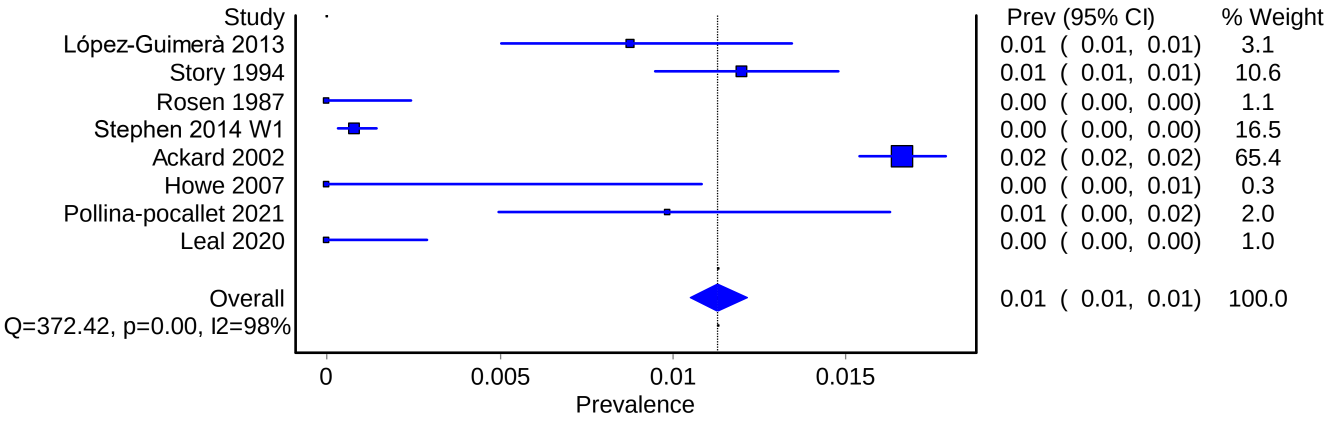

Males – lifetime use laxative

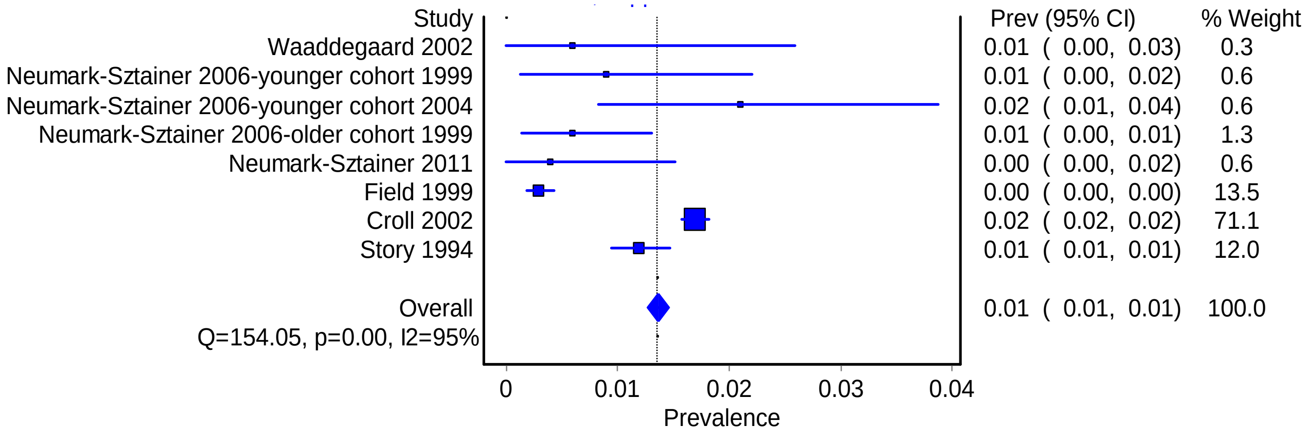

Males – past week diuretic

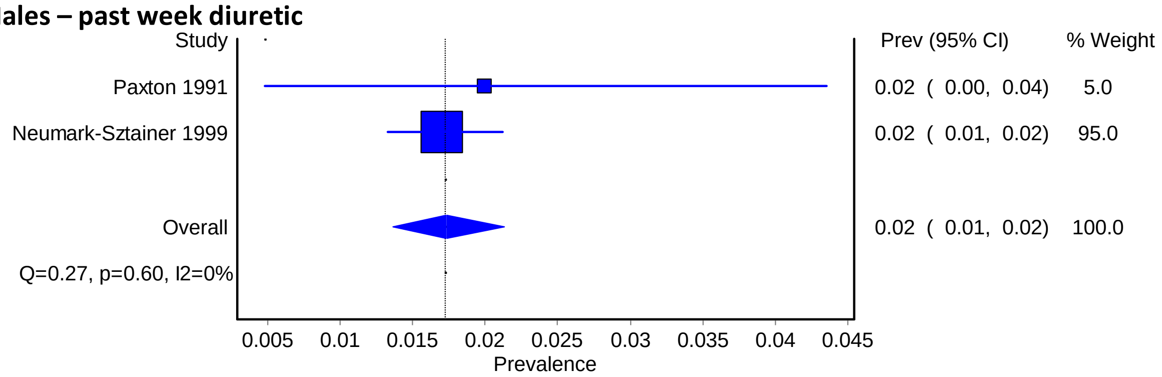

Males – past 30 day use diuretic

© 2024 Hall NY et al. *JAMA Network Open*.

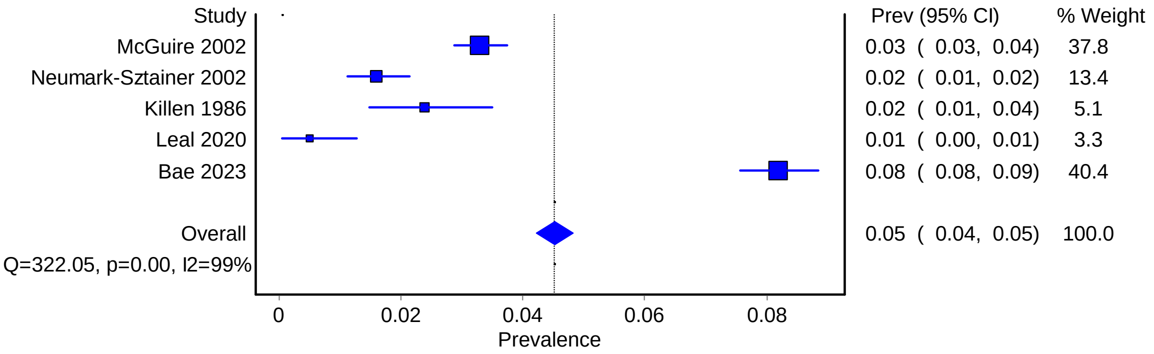

Males – past year use diuretic

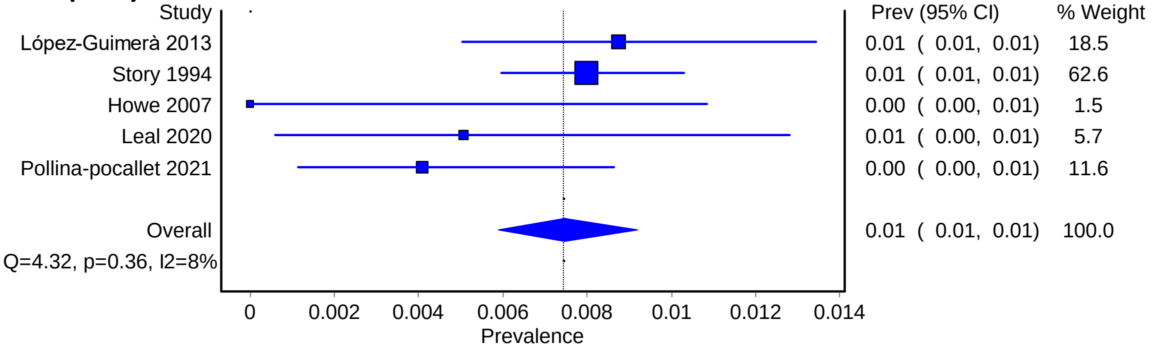

Males – lifetime use diuretic

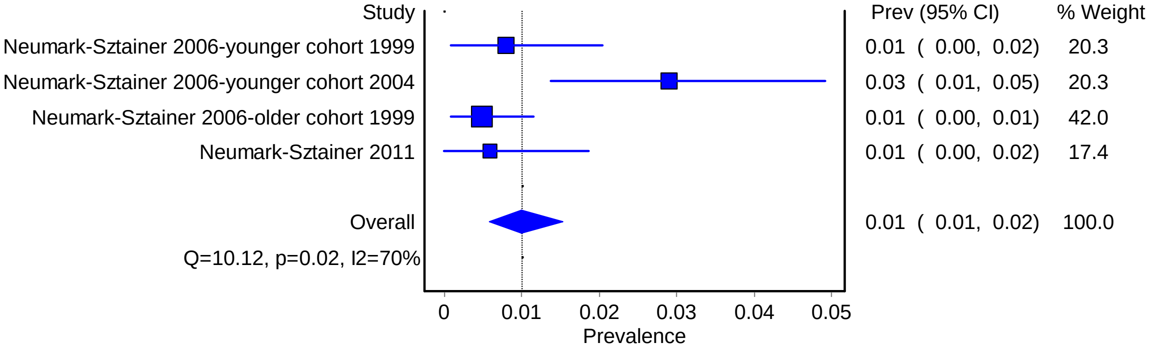

Table of prevalence results for non-prescription weight loss product use for males and females separately

Non-prescription weight loss product use				
	**Past week prevalence (95% CI, n)**	**Past month prevalence (95% CI, n)**	**Past year prevalence (95% CI, n)**	**Lifetime use prevalence (95% CI, n)**
**Males**	2.8% (2.6%, 3.0%, 5)	3.4% (3.4%, 3.6%, 18)	3.3% (3.2%, 3.4%, 14)	3.2% (3.1%, 3.4%, 9)
**Females**	5.4% (5.3%, 5.5%, 8)	5.8% (5.7%, 5.9%, 25)	8.6% (8.5%, 8.8%, 16)	9.5% (9.2%, 9.7%, 17)

Appendix 11

eFigure 8. Meta-Analysis Results for Differences in Type of Weight Loss Product Use

Diet pills – past week use

© 2024 Hall NY et al. *JAMA Network Open*.

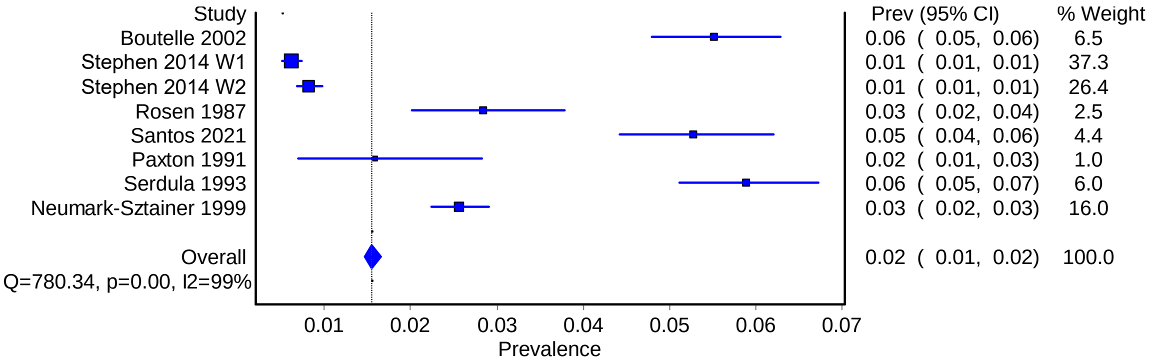

Diet pills – past month use

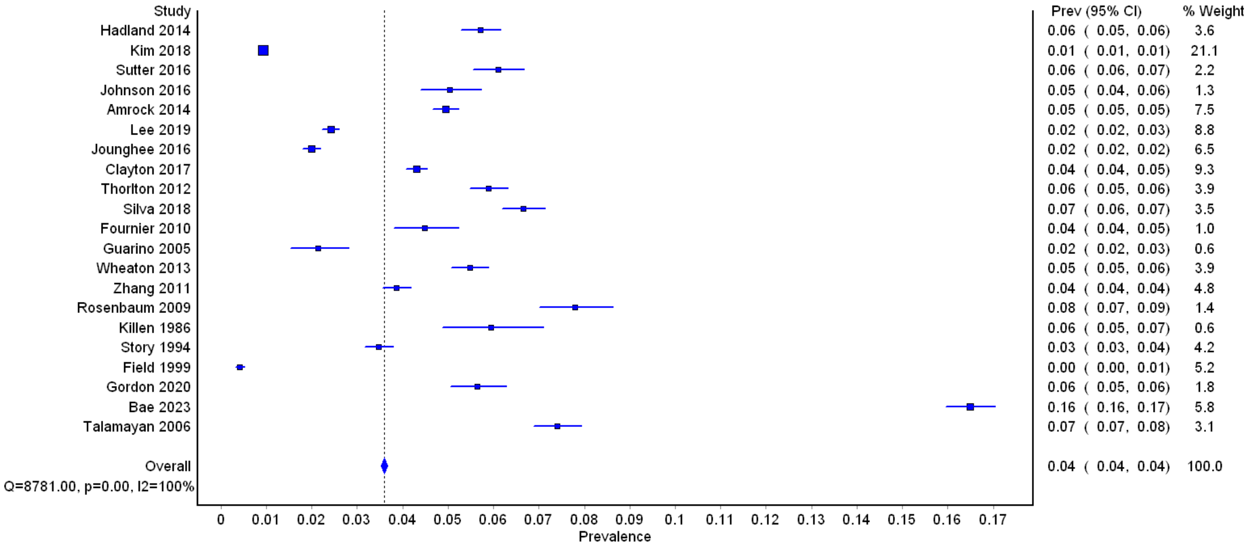

Diet pills – past year use

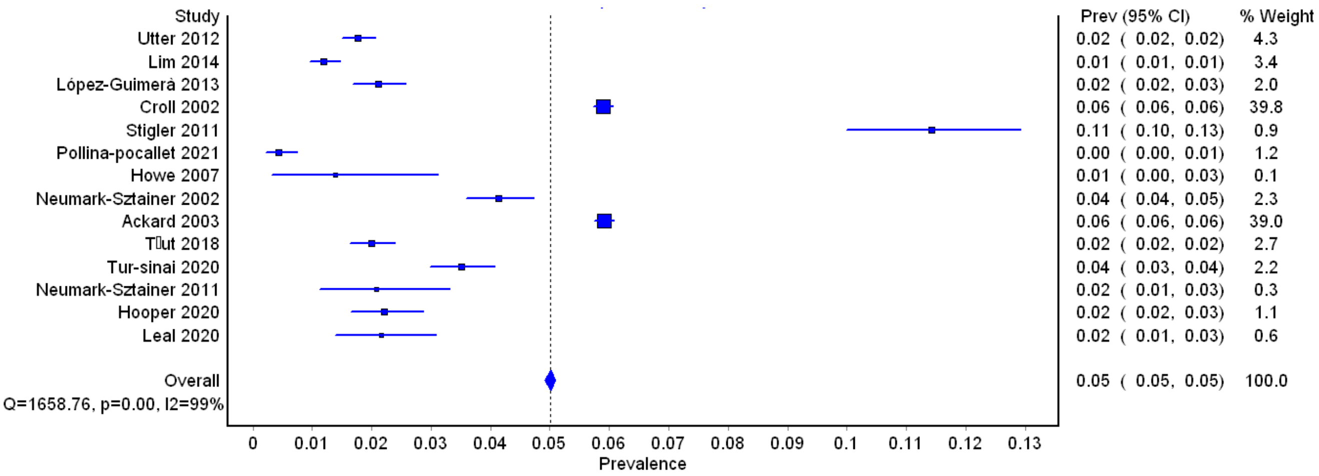

Diet pills – lifetime use

© 2024 Hall NY et al. *JAMA Network Open*.

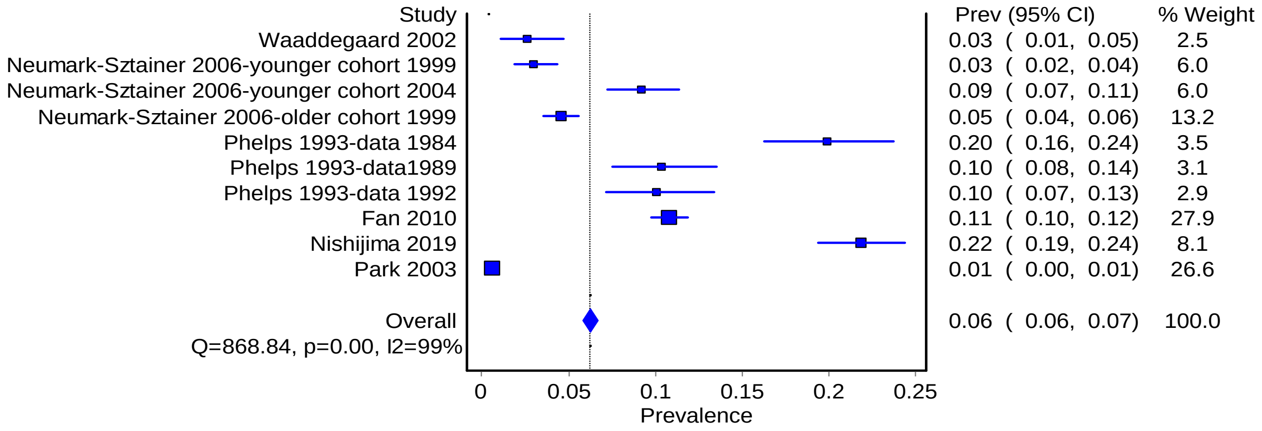

Diuretics – past year use

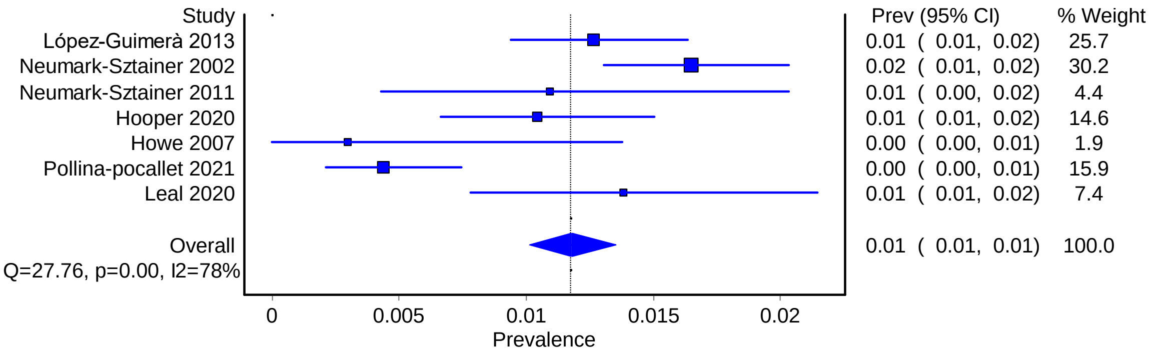

Diuretics – lifetime use

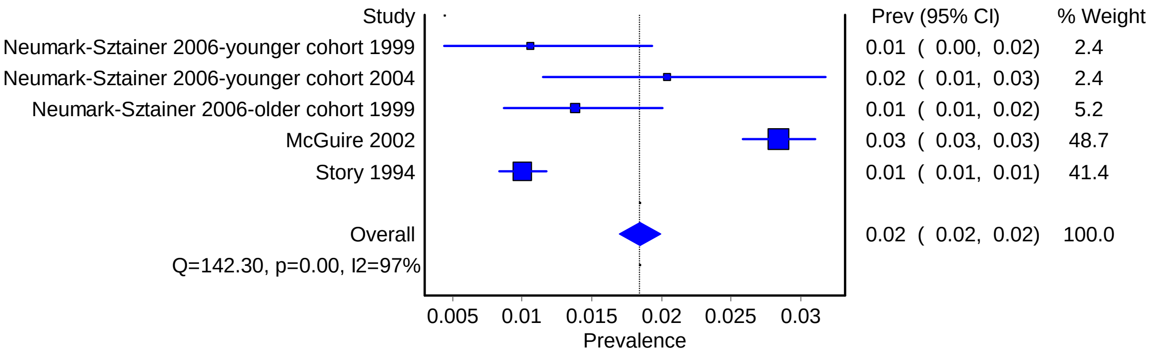

Laxatives – past week use

© 2024 Hall NY et al. *JAMA Network Open*.

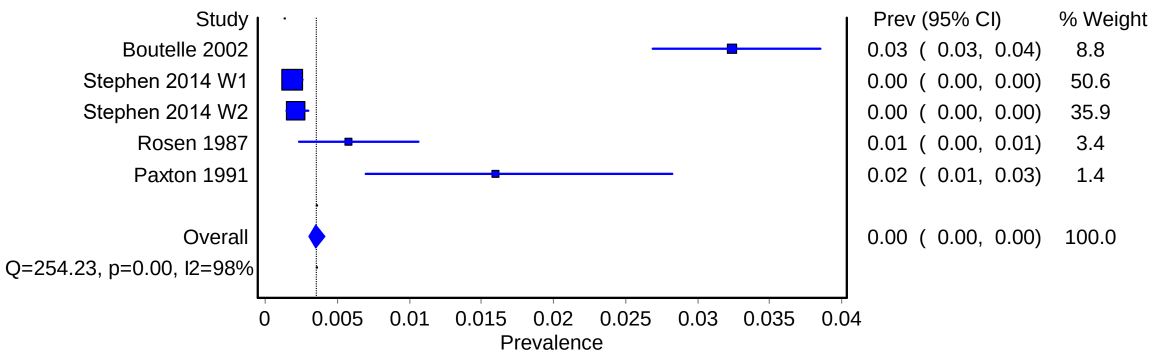

Laxatives – past month use

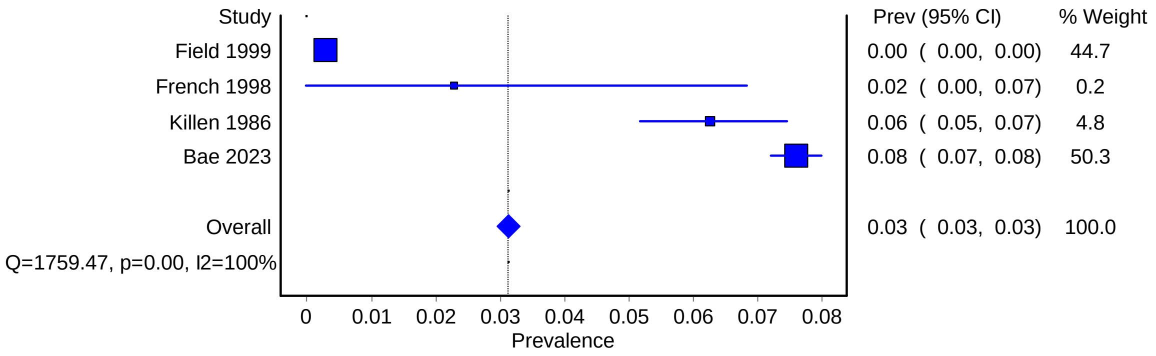

Laxatives – past year use

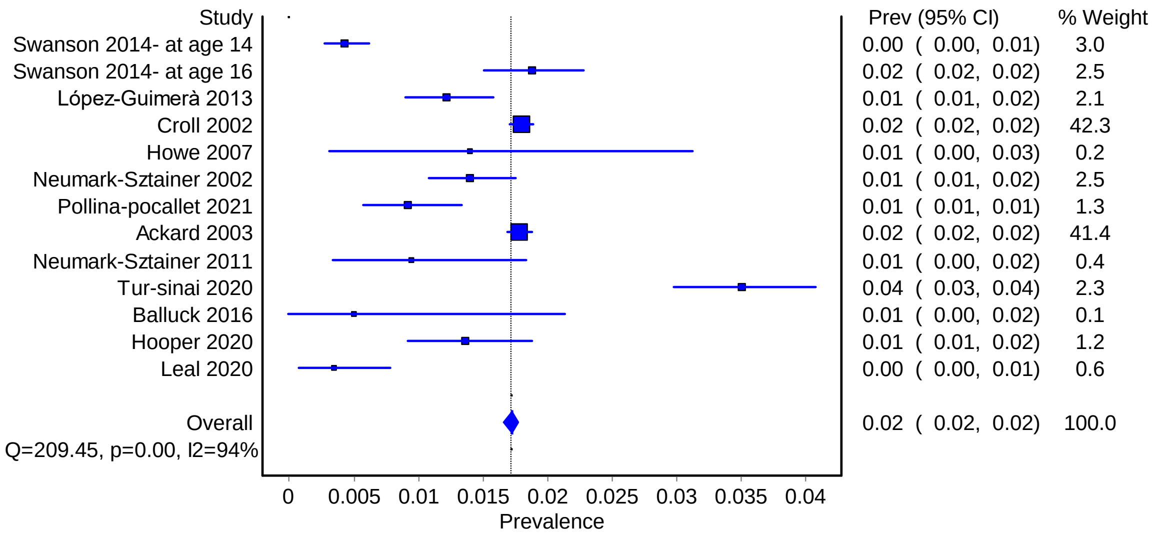

Laxatives – lifetime use

© 2024 Hall NY et al. *JAMA Network Open*.

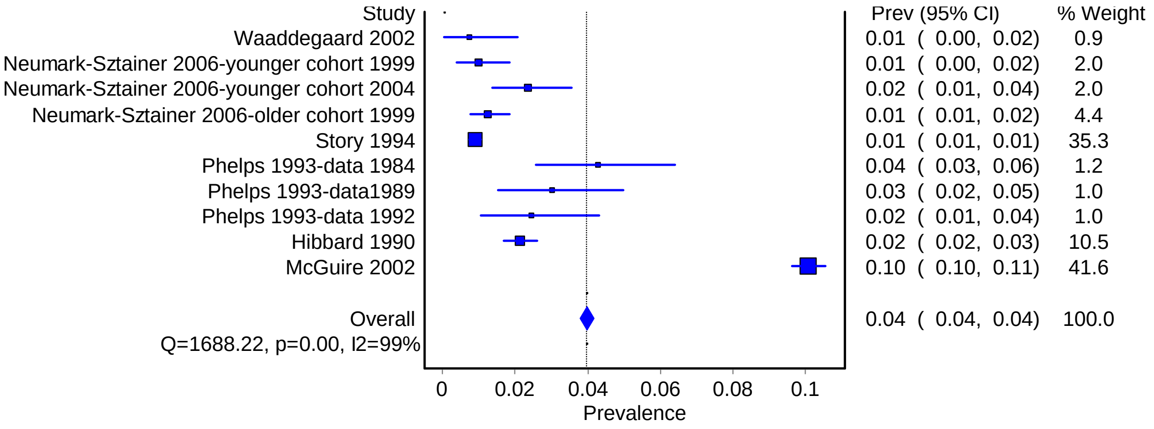

Table of prevalence results for different types of non-prescription weight loss products

Non-prescription weight loss product use				
	**Past week prevalence (95% CI, n)**	**Past month prevalence (95% CI, n)**	**Past year prevalence (95% CI, n)**	**Lifetime use prevalence (95% CI, n)**
**Diet pills**	8	21	14	10
**Laxatives**	0.4% (0.3%, 0.4%, 5)	3.1% (3.0%, 3.3%, 4)	1.7% (1.6%, 1.8%, 13)	4.0% (3.8%, 4.2%, 10)
**Diuretics**	N/A[1]	N/A	1.2% (1.0%, 1.4%, 7)	1.8% (1.7%, 2.0%, 5)

1 – N/A is reported when a meta-analysis could not be completed. For example, when one or no studies were found in the specified sub-group.

Appendix 12

eFigure 9. Meta-Analysis Results for Continent Differences in Weight Loss Product Use

Past week use

North America (USA)

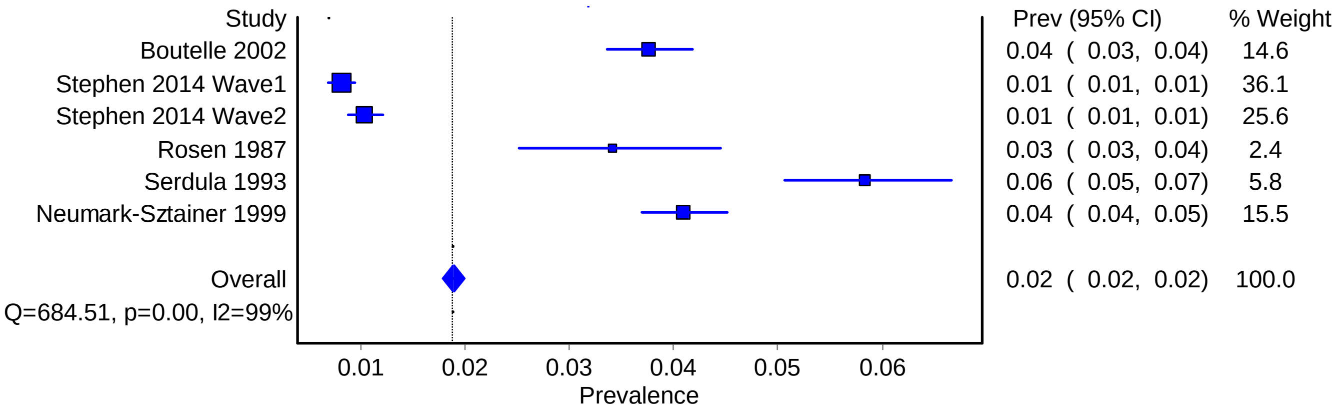

Past month use

North America (USA and Jamaica)

© 2024 Hall NY et al. *JAMA Network Open*.

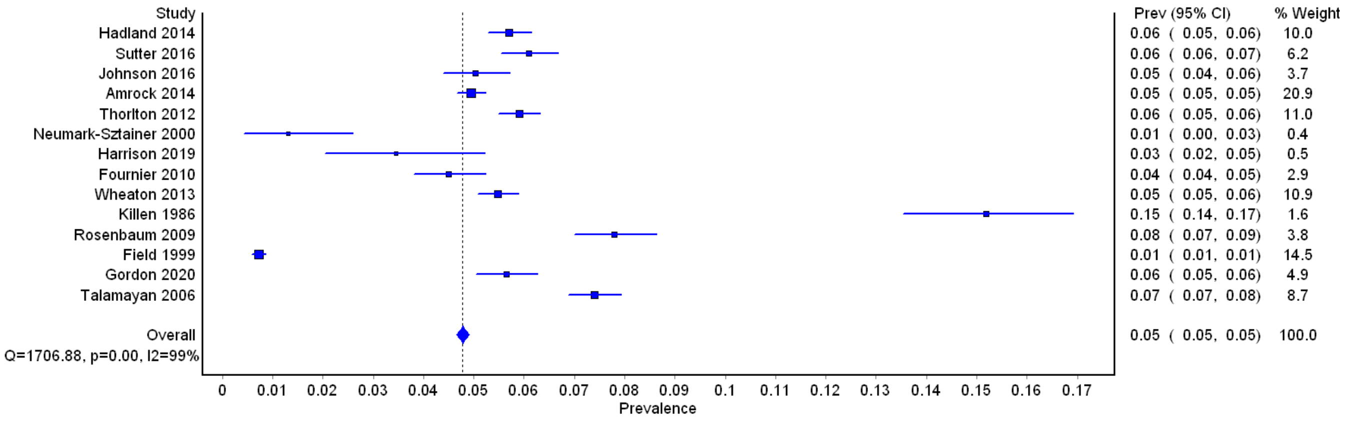

Asia (Korea and China)

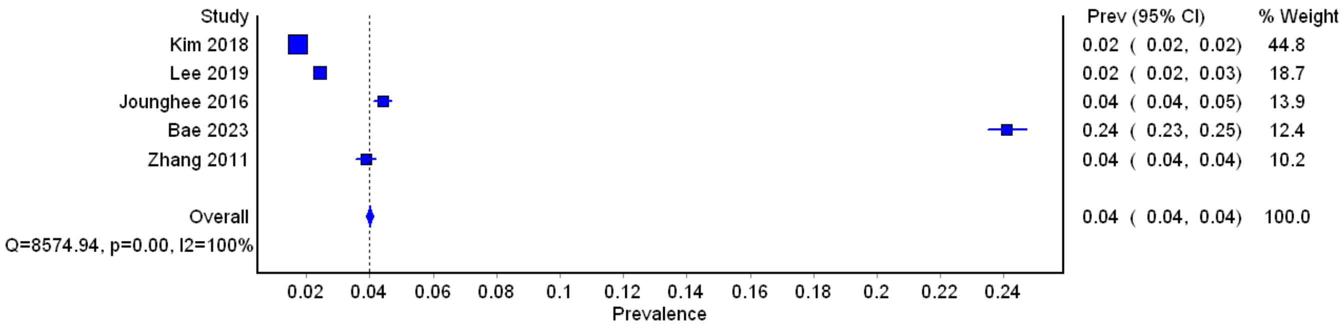

South America (Colombia and Brazil)

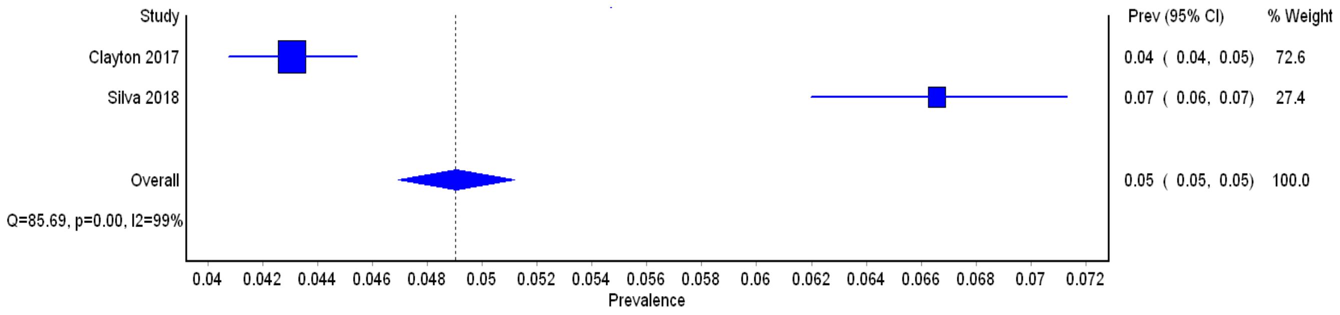

Past year use

Europe (UK, Romania and Spain)

© 2024 Hall NY et al. *JAMA Network Open*.

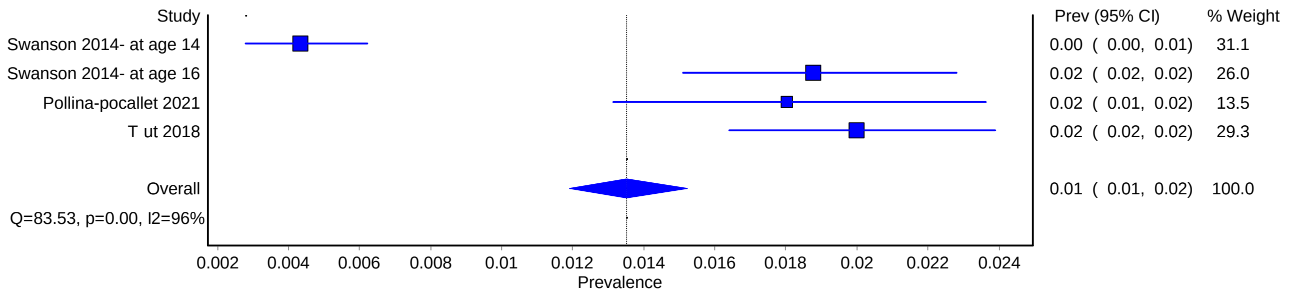

North America (USA)

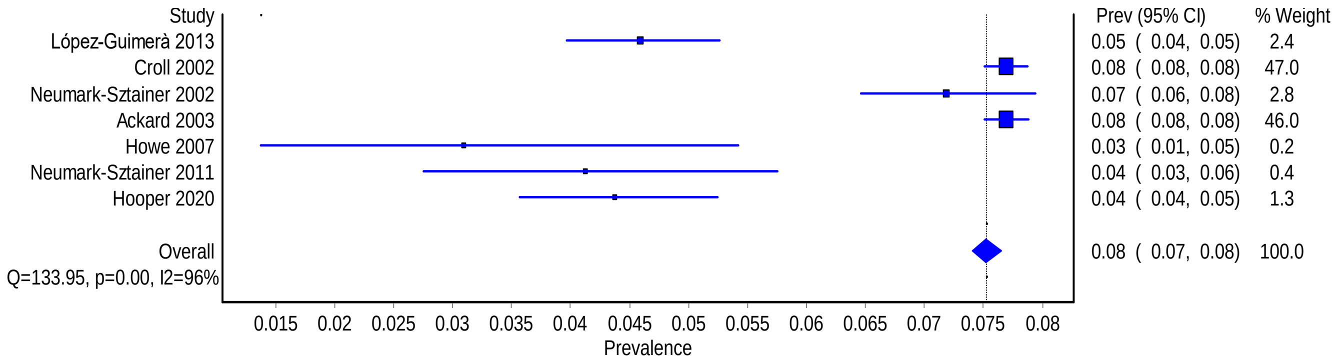

Asia (Korea, India and Israel)

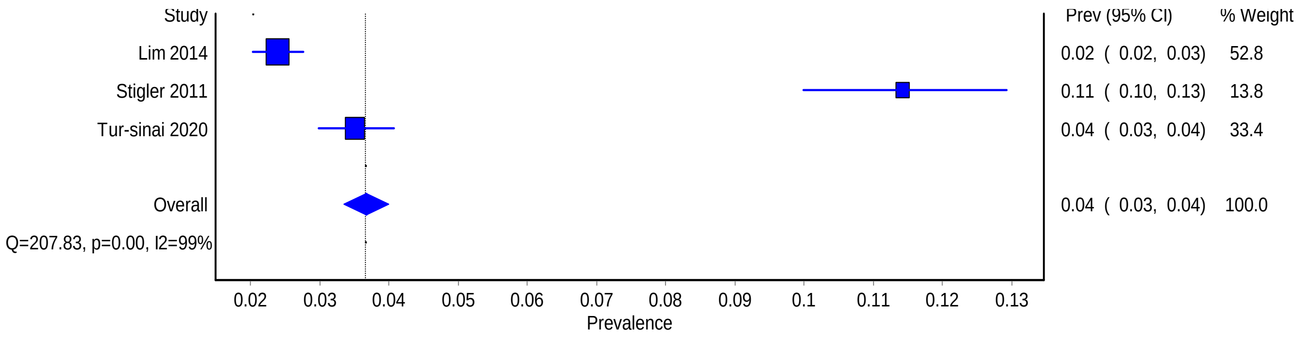

Lifetime use

North America (USA and the Caribbean)

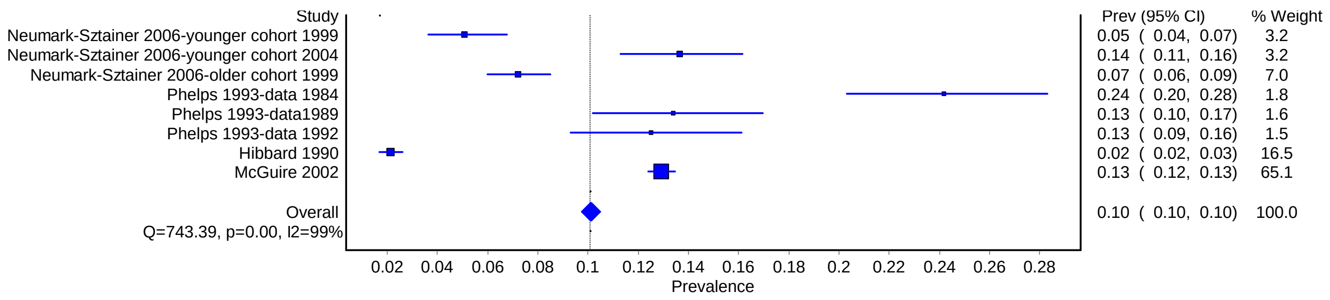

Asia (China, Israel, Japan, Korea)

© 2024 Hall NY et al. *JAMA Network Open*.

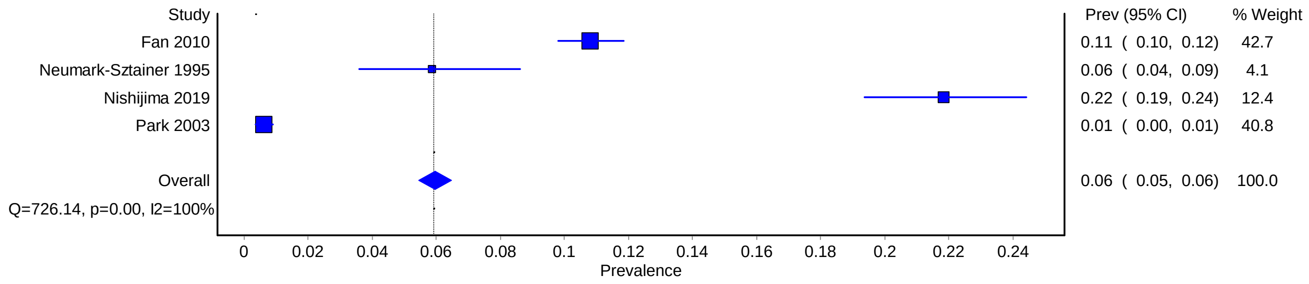

Table of prevalence results for non-prescription weight loss product use per continent

Non-prescription weight loss product use				
	**Past week prevalence (95% CI, n)**	**Past month prevalence (95% CI, n)**	**Past year prevalence (95% CI, n)**	**Lifetime use prevalence (95% CI, n)**
**North America**	1.9% (1.8%, 2.0%, 8)	4.8% (4.7%, 4.9%, 14)	7.5% (7.4%, 7.7%, 7)	10.1% (9.7%, 10.5%, 8)
**Asia**	N/A[1]	4.0% (3.9%, 4.1%, 5)	3.7% (3.4%, 4.0%, 3)	5.9% (5.4%, 6.5%, 4)
**South America**	N/A	4.9% (4.7%, 5.1%, 2)	N/A	N/A
**Europe**	N/A	N/A	1.4% (1.2%, 1.5%, 4)	N/A

1 – N/A is reported when a meta-analysis could not be completed. For example, when one or no studies were found in the specified sub-group.

Appendix 13

eFigure 10. Meta-Analysis Results for Weight Loss Product Use With Respect to Different Study Publication Years

Studies published prior to 2000

Past week

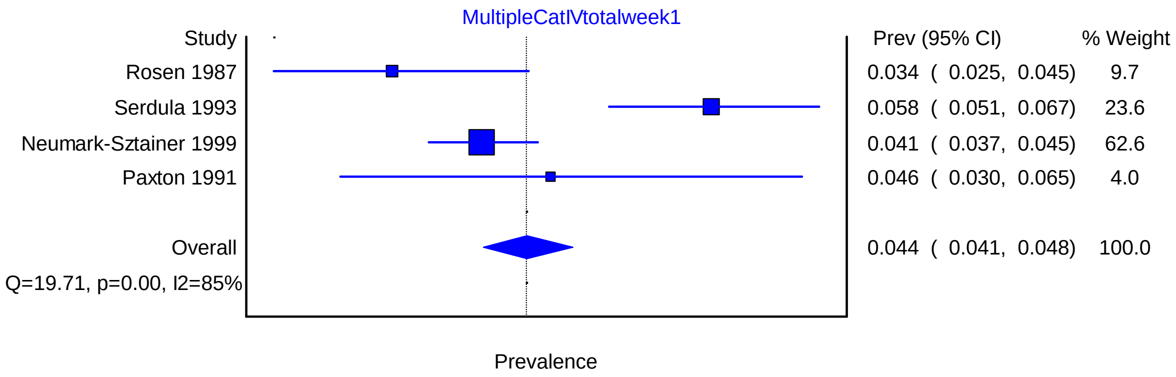

Past month

© 2024 Hall NY et al. *JAMA Network Open*.

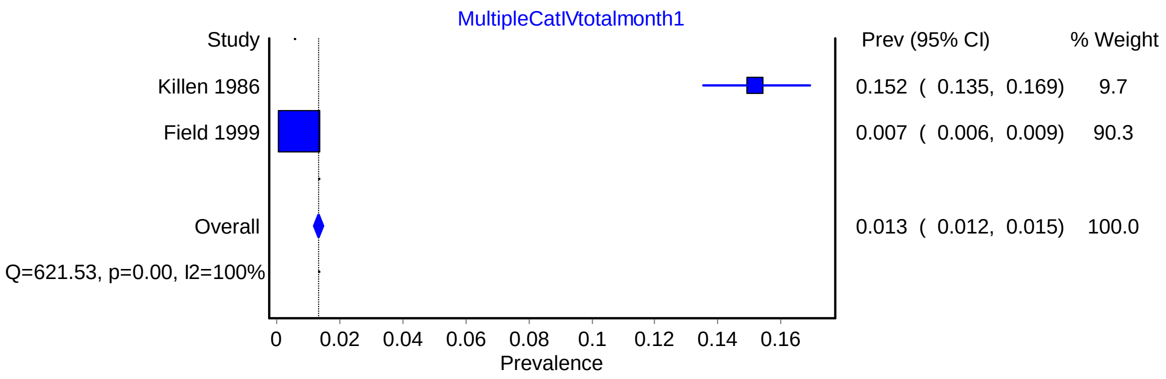

Past year

Not applicable – all studies were published after the year 2000

Lifetime

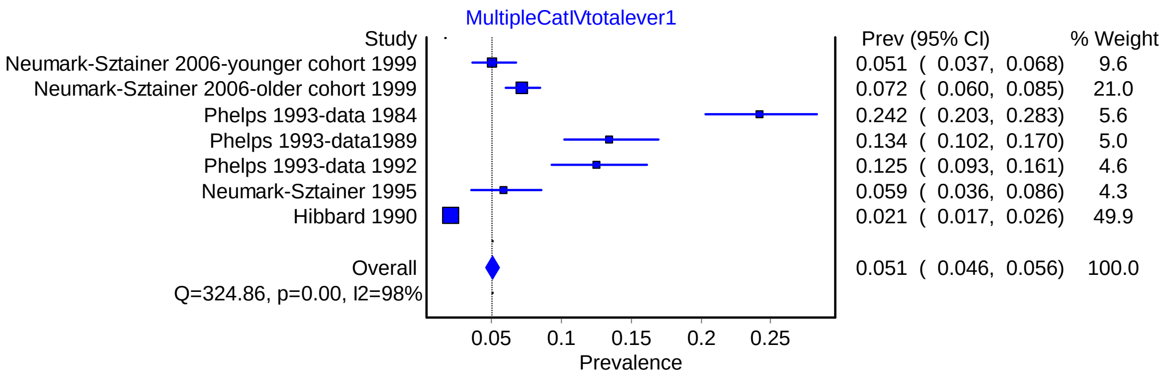

Studies published after the year 2000

Past week

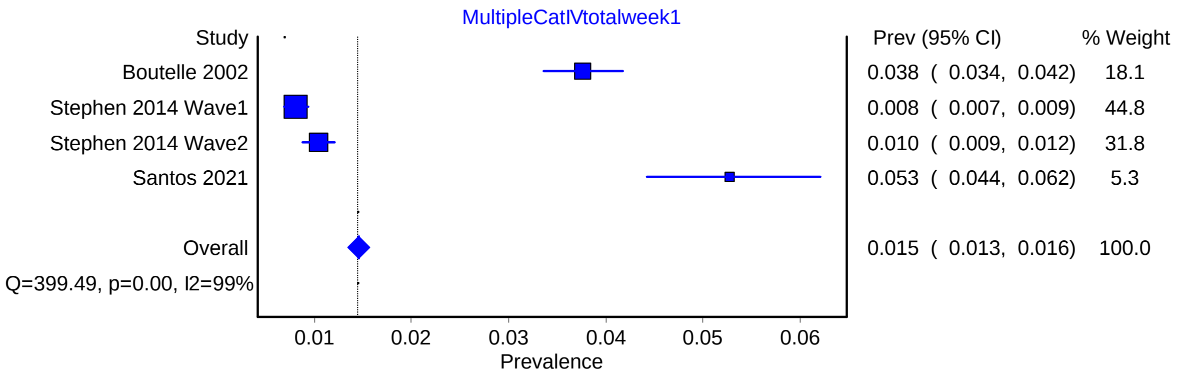

Past month

© 2024 Hall NY et al. *JAMA Network Open*.

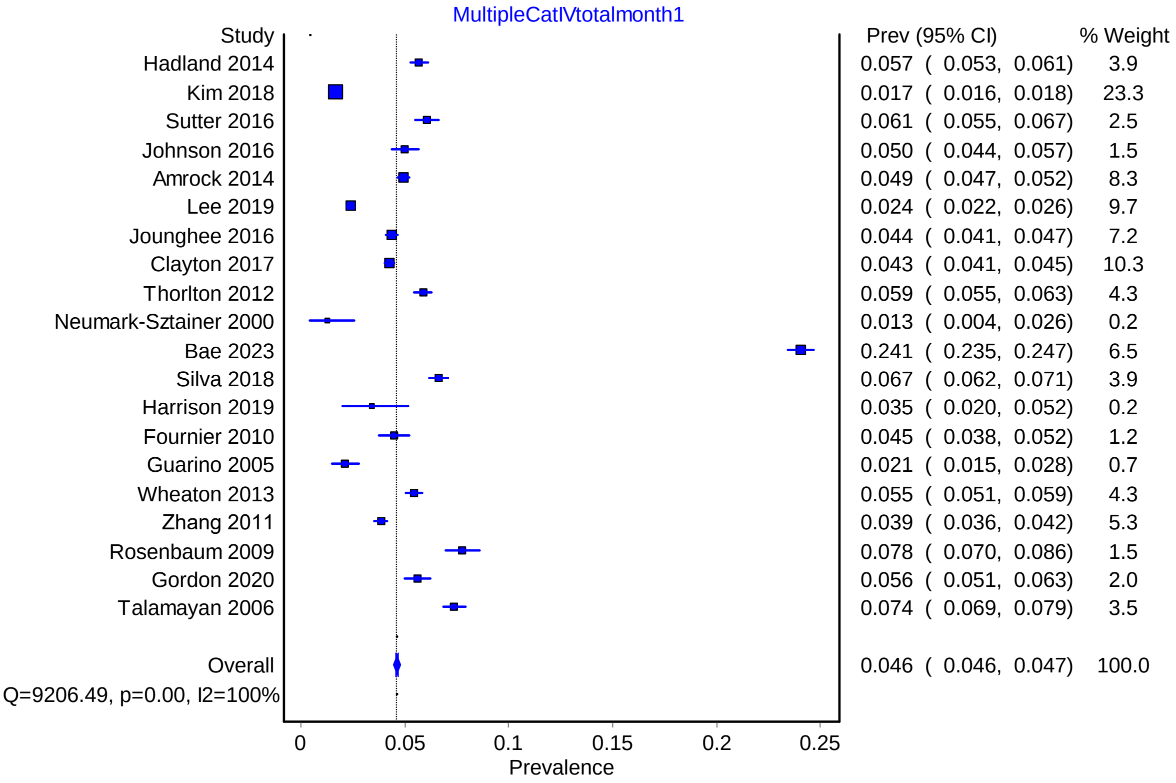

Past year

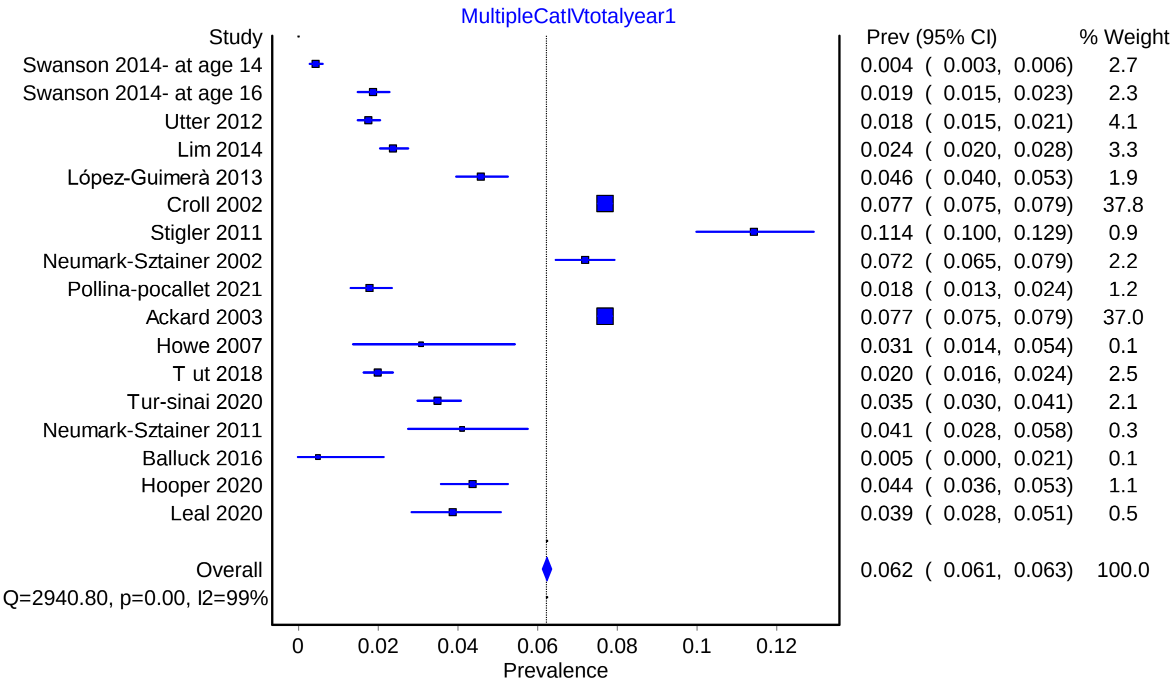

Lifetime

© 2024 Hall NY et al. *JAMA Network Open*.

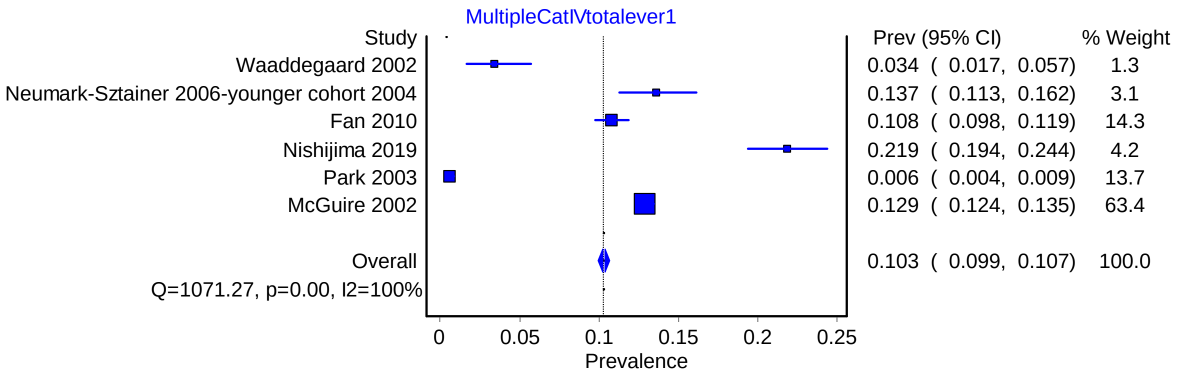

Studies published after the year 2010

Past week

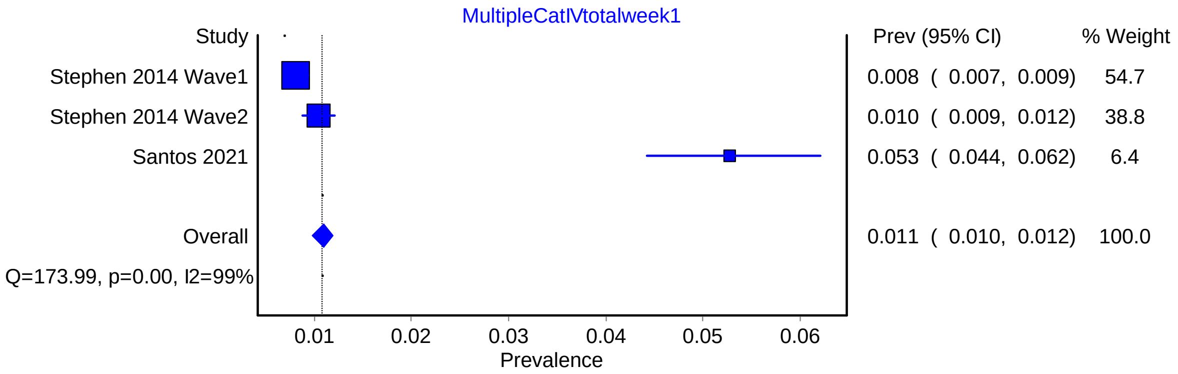

Past month

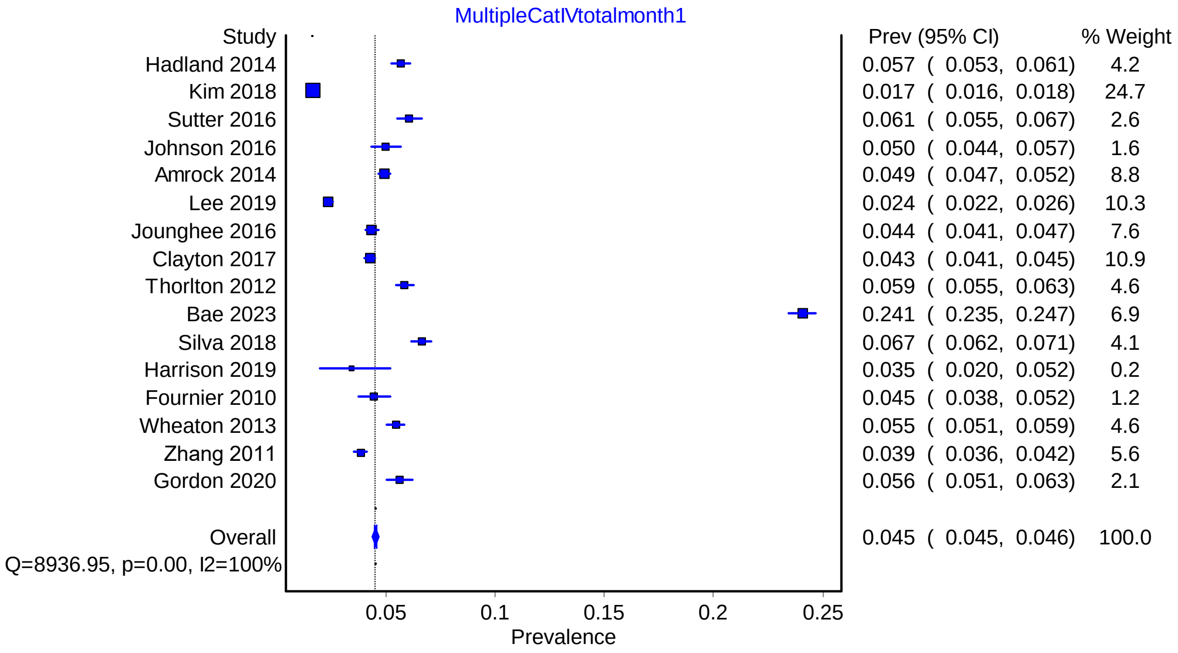

Past year

© 2024 Hall NY et al. *JAMA Network Open*.

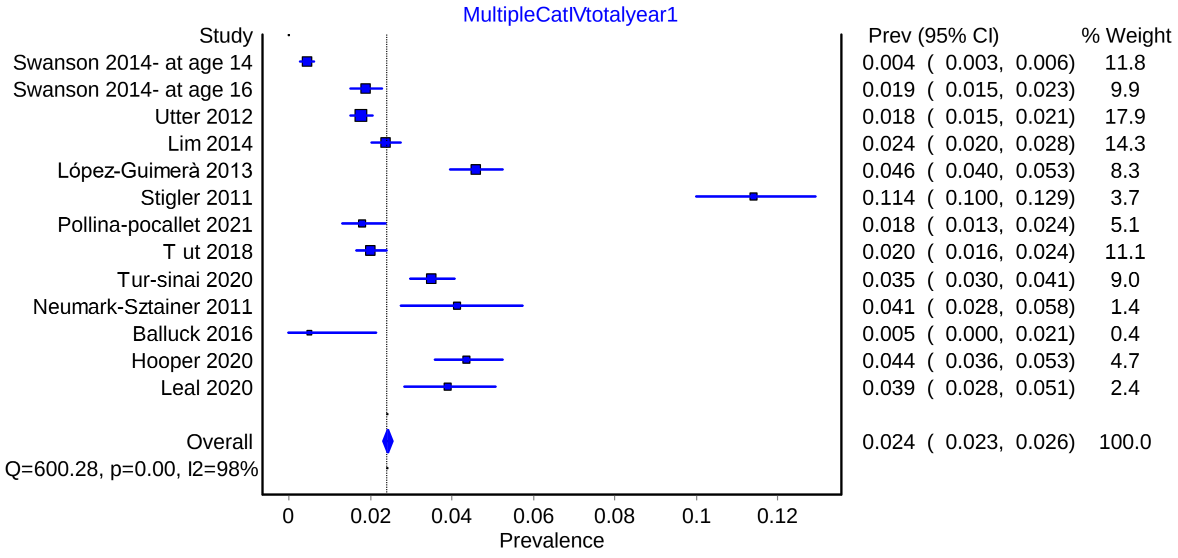

Lifetime

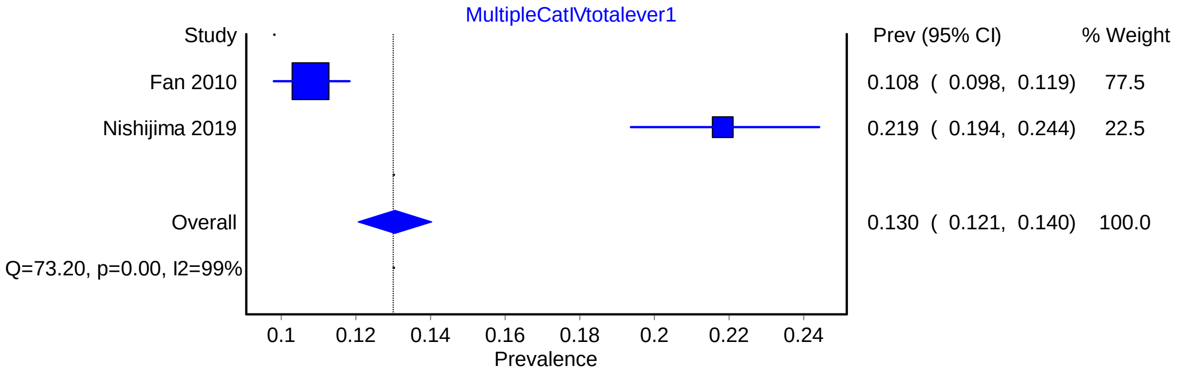

Table of prevalence results for non-prescription weight loss product use for studies published prior to the year 2000, studies published after the year 2000 and studies published after the year 2010

Non-prescription weight loss product use				
	**Past week prevalence (95% CI, n)**	**Past month prevalence (95% CI, n)**	**Past year prevalence (95% CI, n)**	**Lifetime use prevalence (95% CI, n)**
**Prior to year 2000**	4.4% (4.1%, 4.8%, 4)	1.3% (1.2%, 1.5%, 2)	N/A[1]	5.1% (4.6%, 5.6%, 7)
**Post year 2000**	1.5% (1.3%, 1.6%, 4)	4.6% (4.6%, 4.7%, 20)	6.2% (6.1%, 6.3%17)	10.3% (9.9%, 10.7%, 6)
**Post year 2010**	1.1% (1.0%, 1.2%, 3)	4.5% (4.5%, 4.6%, 16)	2.4% (2.3%, 2.6%, 13)	13.0% (12.1%, 14.0%, 2)

1 – N/A is reported when a meta-analysis could not be completed. For example, when one or no studies were found in the specified sub-group.

Appendix 14

eFigure 11. Meta-Analysis Results With the Removal of the 3 Poor Quality Studies (32, 55, and 79)

© 2024 Hall NY et al. *JAMA Network Open*.

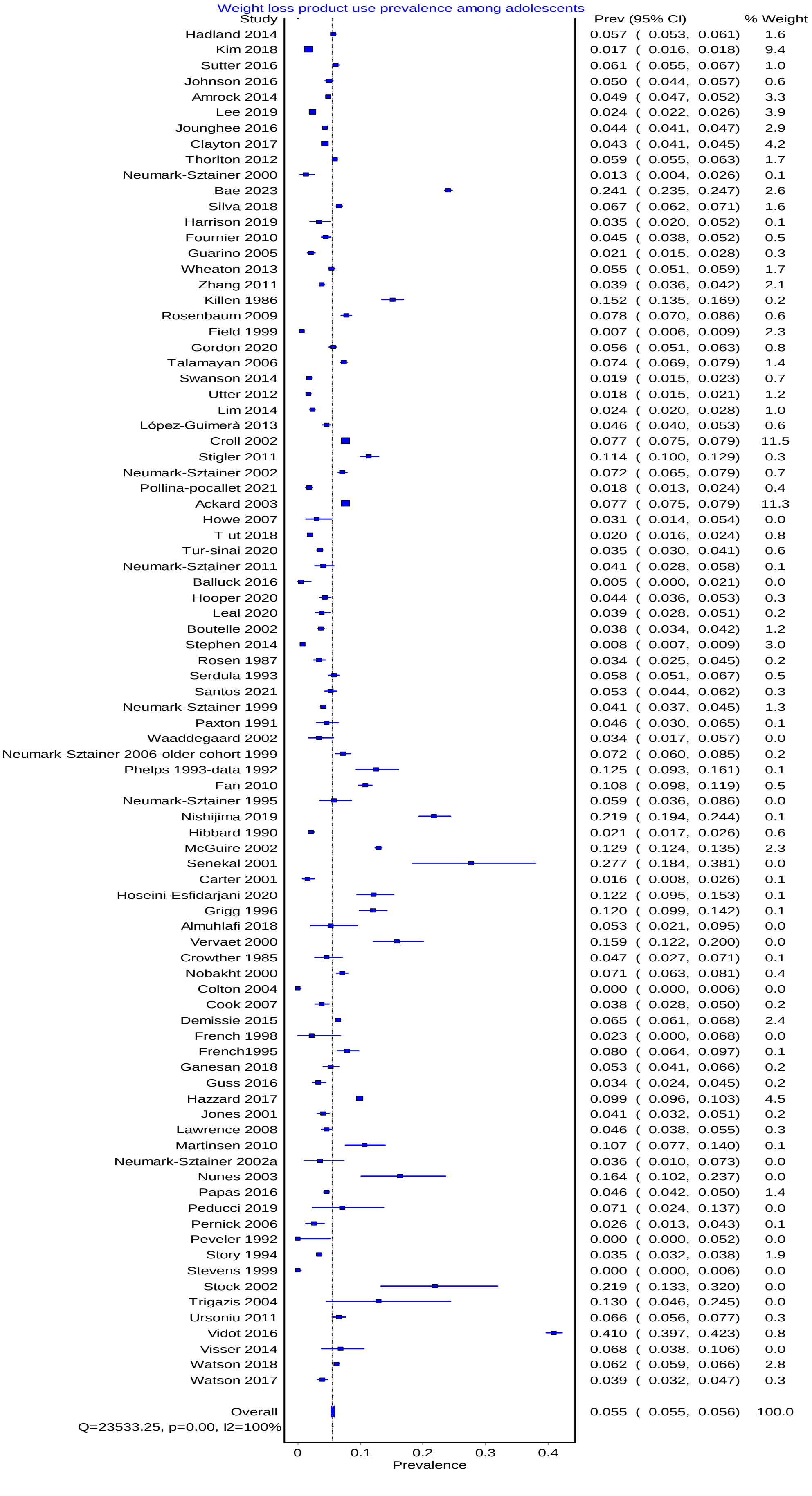
Weight loss product use prevalence among adolescents
Study
Prev (95% CI)
% Weight
Hadland 2014 0.057 (0.053, 0.061) 1.6
Kim 2018 0.017 (0.016, 0.018) 9.4
Sutter 2016 0.061 (0.055, 0.067) 1.0
Johnson 2016 0.050 (0.044, 0.057) 0.6
Amrock 2014 0.049 (0.047, 0.052) 3.3
Lee 2019 0.024 (0.022, 0.026) 3.9
Jounghee 2016 0.044 (0.041, 0.047) 2.9
Clayton 2017 0.043 (0.041, 0.045) 4.2
Thorlton 2012 0.059 (0.055, 0.063) 1.7
Neumark-Sztainer 2000 0.013 (0.004, 0.026) 0.1
Bae 2023 0.241 (0.235, 0.247) 2.6
Silva 2018 0.067 (0.062, 0.071) 1.6
Harrison 2019 0.035 (0.020, 0.052) 0.1
Fournier 2010 0.045 (0.038, 0.052) 0.5
Guarino 2005 0.021 (0.015, 0.028) 0.3
Wheaton 2013 0.055 (0.051, 0.059) 1.7
Zhang 2011 0.039 (0.036, 0.042) 2.1
Killen 1986 0.152 (0.135, 0.169) 0.2
Rosenbaum 2009 0.078 (0.070, 0.086) 0.6
Field 1999 0.007 (0.006, 0.009) 2.3
Gordon 2020 0.056 (0.051, 0.063) 0.8
Talamayan 2006 0.074 (0.069, 0.079) 1.4
Swanson 2014 0.019 (0.015, 0.023) 0.7
Utter 2012 0.018 (0.015, 0.021) 1.2
Lim 2014 0.024 (0.020, 0.028) 1.0
López-Guimerà 2013 0.046 (0.040, 0.053) 0.6
Croll 2002 0.077 (0.075, 0.079) 11.5
Stigler 2011 0.114 (0.100, 0.129) 0.3
Neumark-Sztainer 2002 0.072 (0.065, 0.079) 0.7
Pollina-pocallet 2021 0.018 (0.013, 0.024) 0.4
Ackard 2003 0.077 (0.075, 0.079) 11.3
Howe 2007 0.031 (0.014, 0.054) 0.0
T ut 2018 0.020 (0.016, 0.024) 0.8
Tur-sinai 2020 0.035 (0.030, 0.041) 0.6
Neumark-Sztainer 2011 0.041 (0.028, 0.058) 0.1
Balluck 2016 0.005 (0.000, 0.021) 0.0
Hooper 2020 0.044 (0.036, 0.053) 0.3
Leal 2020 0.039 (0.028, 0.051) 0.2
Boutelle 2002 0.038 (0.034, 0.042) 1.2
Stephen 2014 0.008 (0.007, 0.009) 3.0
Rosen 1987 0.034 (0.025, 0.045) 0.2
Serdula 1993 0.058 (0.051, 0.067) 0.5
Santos 2021 0.053 (0.044, 0.062) 0.3
Neumark-Sztainer 1999 0.041 (0.037, 0.045) 1.3
Paxton 1991 0.046 (0.030, 0.065) 0.1
Waaddegaard 2002 0.034 (0.017, 0.057) 0.0
Neumark-Sztainer 2006-older cohort 1999 0.072 (0.060, 0.085) 0.2
Phelps 1993-data 1992 0.125 (0.093, 0.161) 0.1
Fan 2010 0.108 (0.098, 0.119) 0.5
Neumark-Sztainer 1995 0.059 (0.036, 0.086) 0.0
Nishijima 2019 0.219 (0.194, 0.244) 0.1
Hibbard 1990 0.021 (0.017, 0.026) 0.6
McGuire 2002 0.129 (0.124, 0.135) 2.3
Senekal 2001 0.277 (0.184, 0.381) 0.0
Carter 2001 0.016 (0.008, 0.026) 0.1
Hoseini-Esfidarjani 2020 0.122 (0.095, 0.153) 0.1
Grigg 1996 0.120 (0.099, 0.142) 0.1
Almuhlafi 2018 0.053 (0.021, 0.095) 0.0
Vervaet 2000 0.159 (0.122, 0.200) 0.0
Crowther 1985 0.047 (0.027, 0.071) 0.1
Nobakht 2000 0.071 (0.063, 0.081) 0.4
Colton 2004 0.000 (0.000, 0.006) 0.0
Cook 2007 0.038 (0.028, 0.050) 0.2
Demissie 2015 0.065 (0.061, 0.068) 2.4
French 1998 0.023 (0.000, 0.068) 0.0
French1995 0.080 (0.064, 0.097) 0.1
Ganesan 2018 0.053 (0.041, 0.066) 0.2
Guss 2016 0.034 (0.024, 0.045) 0.2
Hazzard 2017 0.099 (0.096, 0.103) 4.5
Jones 2001 0.041 (0.032, 0.051) 0.2
Lawrence 2008 0.046 (0.038, 0.055) 0.3
Martinsen 2010 0.107 (0.077, 0.140) 0.1
Neumark-Sztainer 2002a 0.036 (0.010, 0.073) 0.0
Nunes 2003 0.164 (0.102, 0.237) 0.0
Papas 2016 0.046 (0.042, 0.050) 1.4
Peducci 2019 0.071 (0.024, 0.137) 0.0
Pernick 2006 0.026 (0.013, 0.043) 0.1
Peveler 1992 0.000 (0.000, 0.052) 0.0
Story 1994 0.035 (0.032, 0.038) 1.9
Stevens 1999 0.000 (0.000, 0.006) 0.0
Stock 2002 0.219 (0.133, 0.320) 0.0
Trigazis 2004 0.130 (0.046, 0.245) 0.0
Ursoniu 2011 0.066 (0.056, 0.077) 0.3
Vidot 2016 0.410 (0.397, 0.423) 0.8
Visser 2014 0.068 (0.038, 0.106) 0.0
Watson 2018 0.062 (0.059, 0.066) 2.8
Watson 2017 0.039 (0.032, 0.047) 0.3
Overall 0.055 (0.055, 0.056) 100.0
Q=23533.25, p=0.00, I2=100%
0
0.1
0.2
0.3
0.4
Prevalence

eReferences

1. Ackard DM, Neumark-Sztainer D. Overeating among adolescents: prevalence and associations with weight-related characteristics and psychological health. Pediatrics. 2003;111(1):67.
2. Almuhlafi M, Jamilah KA, Almutairi AF, Salam M. Relationship between early menarche, obesity, and disordered eating behaviors: a school-based cross-sectional survey in Northern Saudi Arabia. Diabetes Metab Syndr Obes. 2018;11:743-51.
3. Amrock SM, Weitzman M. Adolescent indoor tanning use and unhealthy weight control behaviors. Journal of developmental and behavioral pediatrics : JDBP. 2014;35(3):165-71.
4. Bae EJ, Yoon JY. Unhealthy weight control behaviors and related factors by gender and weight status: Results from a nationally representative sample of Korean adolescents. Archives of Psychiatric Nursing. 2023;42:75-83.
5. Balluck G, Toorabally BZ, Hosenally M. Association Between Body Image Dissatisfaction and Body Mass Index, Eating Habits and Weight Control Practices among Mauritian Adolescents. Malaysian Journal of Nutrition. 2016;22(3):389-401.
6. Boutelle K, Neumark-Sztainer D, Story M, Resnick M. Weight control behaviors among obese, overweight, and nonoverweight adolescents. Journal of Pediatric Psychology. 2002;27(6):531-40.
7. Carter JC, Stewart DA, Fairburn CG. Eating disorder examination questionnaire: norms for young adolescent girls. Behav Res Ther. 2001;39(5):625-32.
8. Clayton HB, Demissie Z, Lowry R, Lundeen EA, Sharma AJ, Bohm MK. Unhealthy weight management practices and non-medical use of prescription drugs. American Journal of Preventive Medicine. 2017;52(2):215-9.
9. Colton P, Olmsted M, Daneman D, Rydall A, Rodin G. Disturbed Eating Behavior and Eating Disorders in Preteen and Early Teenage Girls With Type 1 Diabetes : A case-controlled study. Diabetes Care. 2004;27(7):1654-9.
10. Sarah Jane C, Kathleen M, Donald BL. Far from ideal. Canadian Family Physician. 2007;53(4):678.
11. Croll J, Neumark-Sztainer D, Story M, Ireland M. Prevalence and risk and protective factors related to disordered eating behaviors among adolescents: Relationship to gender and ethnicity. Journal of Adolescent Health. 2002;31(2):166-75.
12. Crowther JH, Post G, Zaynor L. The prevalence of bulimia and binge eating in adolescent girls. International Journal of Eating Disorders. 1985;4(1):29-42.
13. Demissie Z, Lowry R, Eaton DK, Nihiser AJ. Trends in Weight Management Goals and Behaviors Among 9th–12th Grade Students: United States, 1999–2009. Maternal and Child Health Journal. 2015;19(1):74-83.
14. Fan Y, Li Y, Liu A, Hu X, Ma G, Xu G. Associations between body mass index, weight control concerns and behaviors, and eating disorder symptoms among non-clinical Chinese adolescents. BMC public health. 2010;10:314.
15. Field AE, Camargo CA, Jr., Taylor CB, Berkey CS, Colditz GA. Relation of Peer and Media Influences to the Development of Purging Behaviors Among Preadolescent and Adolescent Girls. Archives of Pediatrics & Adolescent Medicine. 1999;153(11):1184-9.
16. Fournier ME, Austin SB, Samples CL, Goodenow CS, Wylie SA, Corliss HL. A comparison of weight-related behaviors among high school students who are homeless and non-homeless. The Journal of school health. 2009;79(10):466-73.
17. French SA, Peterson CB, Story M, Anderson N, Mussell MP, Mitchell JE. Agreement between survey and interview measures of weight control practices in adolescents. Int J Eat Disord. 1998;23(1):45-56.
18. French SA, Story M, Downes B, Resnick MD, Blum RW. Frequent dieting among adolescents: psychosocial and health behavior correlates. American journal of public health. 1995;85(5):695-701.

© 2024 Hall NY et al. *JAMA Network Open*.

19. Ganesan S, Ravishankar SL, Ramalingam S. Are Body Image Issues Affecting Our Adolescents? A Cross-sectional Study among College Going Adolescent Girls. Indian J Community Med. 2018;43(Suppl 1):S42-s6.
20. Gordon AR, Austin SB, Schultz J, Guss CE, Calzo JP, Wang ML. Gender Expression, Peer Victimization, and Disordered Weight-Control Behaviors Among U.S. High School Students. Journal of Adolescent Health. 2020.
21. Grigg M, Bowman J, Redman S. Disordered Eating and Unhealthy Weight Reduction Practices among Adolescent Females. Preventive Medicine. 1996;25(6):748-56.
22. Guarino R, Pellai A, Bassoli L, Cozzi M, Di Sanzo MA, Campra D, et al. Overweight, thinness, body self-image and eating strategies of 2,121 Italian teenagers. TheScientificWorldJournal. 2005;5:812-9.
23. Guss CE, Williams DN, Reisner SL, Austin SB, Katz-Wise SL. Disordered Weight Management Behaviors, Nonprescription Steroid Use, and Weight Perception in Transgender Youth. Journal of Adolescent Health. 2017;60(1):17-22.
24. Hadland SE, Austin SB, Goodenow CS, Calzo JP. Weight misperception and unhealthy weight control behaviors among sexual minorities in the general adolescent population. Journal of Adolescent Health. 2014;54(3):296-303.
25. Harrison AN, James Bateman CCB, Younger-Coleman NOM, Williams MC, Rocke KD, Clato-Day Scarlett SC, et al. Disordered eating behaviours and attitudes among adolescents in a middle-income country. Eating and Weight Disorders. 2019.
26. Hazzard VM, Simone M, Austin SB, Larson N, Neumark-Sztainer D. Diet pill and laxative use for weight control predicts first-time receipt of an eating disorder diagnosis within the next 5 years among female adolescents and young adults. International Journal of Eating Disorders. 2021;54(7):1289-94.
27. Hibbard RA, Ingersoll GM, Orr DP. Behavioral risk, emotional risk, and child abuse among adolescents in a nonclinical setting. Pediatrics. 1990;86(6):896-901.
28. Hooper L, Telke S, Larson N, Mason SM, Neumark-Sztainer D. Household food insecurity: associations with disordered eating behaviours and overweight in a population-based sample of adolescents. Public health nutrition. 2020;23(17):3126-35.
29. Hoseini-Esfidarjani SS, Negarandeh R, Janani L. The prevalence of weight-control behaviors and associated factors among adolescent girls in Tehran. International journal of adolescent medicine and health. 2020;-1.
30. Howe CJ, Jawad AF, Kelly SD, Lipman TH. Weight-related concerns and behaviors in children and adolescents with type 1 diabetes. J Am Psychiatr Nurses Assoc. 2008;13(6):376-85.
31. Johnson ER, Weiler RM, Barnett TE, Pealer LN. Extreme Weight-Control Behaviors and Suicide Risk Among High School Students. The Journal of school health. 2016;86(4):281-7.
32. Johnson J, Whitaker AH. Adolescent smoking, weight changes, and binge-purge behavior: associations with secondary amenorrhea. American Journal of Public Health. 1992;82(1):47-54.
33. Jones JM, Bennett S, Olmsted MP, Lawson ML, Rodin G. Disordered eating attitudes and behaviours in teenaged girls: a school-based study. Cmaj. 2001;165(5):547-52.
34. Jounghee L, Youngmin L, Lee J, Lee Y. The association of body image distortion with weight control behaviors, diet behaviors, physical activity, sadness, and suicidal ideation among Korean high school students: a cross-sectional study. BMC Public Health. 2016;16(1):1-10.
35. Killen JD, Taylor CB, Teich MJ, Saylor KE, Maron DJ, Robinson TN. Self-induced Vomiting and Laxative and Diuretic Use Among Teenagers: Precursors of the Binge-Purge Syndrome? JAMA. 1986;255(11):1447-9.
36. Kim Y, Austin SB, Subramanian SV, Thomas JJ, Eddy KT, Franko DL, et al. Risk factors for disordered weight control behaviors among Korean adolescents: Multilevel analysis of the Korea Youth Risk Behavior Survey. International Journal of Eating Disorders. 2018;51(2):124-38.

© 2024 Hall NY et al. *JAMA Network Open*.

37. Lawrence JM, Liese AD, Liu L, Dabelea D, Anderson A, Imperatore G, et al. Weight-Loss Practices and Weight-Related Issues Among Youth With Type 1 or Type 2 Diabetes. Diabetes Care. 2008;31(12):2251-7.
38. Leal GVdS, Philippi ST, Alvarenga MDS. Unhealthy weight control behaviors, disordered eating, and body image dissatisfaction in adolescents from São Paulo, Brazil. Revista brasileira de psiquiatria (Sao Paulo, Brazil : 1999). 2020;42(3):264-70.
39. Lee Y, Lee K-S. Relationship between unhealthy weight control behaviors and substance use patterns among Korean adolescents: results from the 2017 national youth risk behavior survey. Public health. 2019;174:56-64.
40. Lim H, Lee H-J, Park S, Kim C-I, Joh H-K, Oh SW. Weight misperception and its association with dieting methods and eating behaviors in South Korean adolescents. Nutrition research and practice. 2014;8(2):213-9.
41. López-Guimerà G, Neumark-Sztainer D, Hannan P, Fauquet J, Loth K, Sánchez-Carracedo D. Unhealthy weight-control behaviours, dieting and weight status: a cross-cultural comparison between North American and Spanish adolescents. European eating disorders review : the journal of the Eating Disorders Association. 2013;21(4):276-83.
42. Martinsen M, Bratland-Sanda S, Eriksson AK, Sundgot-Borgen J. Dieting to win or to be thin? A study of dieting and disordered eating among adolescent elite athletes and non-athlete controls. Br J Sports Med. 2010;44(1):70-6.
43. McGuire MT, Story M, Neumark-Sztainer D, Halcon L, Campbell-Forrester S, Blum RW. Prevalence and correlates of weight-control behaviors among Caribbean adolescent students. The Journal of adolescent health : official publication of the Society for Adolescent Medicine. 2002;31(2):208-11.
44. Neumark-Sztainer D, Palti H, Butler R. Weight concerns and dieting behaviors among high school girls in Israel. Journal of Adolescent Health. 1995;16(1):53-9.
45. Neumark-Sztainer D, Story M, Falkner NH, Beuhring T, Resnick MD. Sociodemographic and personal characteristics of adolescents engaged in weight loss and weight/muscle gain behaviors: Who is doing what? Preventive Medicine. 1999;28(1):40-50.
46. Neumark-Sztainer D, Rock CL, Thornquist MD, Cheskin LJ, Neuhouser ML, Barnett MJ. Weight-control behaviors among adults and adolescents: Associations with dietary intake. Preventive Medicine: An International Journal Devoted to Practice and Theory. 2000;30(5):381-91.
47. Neumark-Sztainer D, Story M, Hannan PJ, Perry CL, Irving LM. Weight-related concerns and behaviors among overweight and nonoverweight adolescents: Implications for preventing weight-related disorders. Archives of Pediatrics and Adolescent Medicine. 2002;156(2):171-8.
48. Neumark-Sztainer D, Wall M, Eisenberg ME, Story M, Hannan PJ. Overweight status and weight control behaviors in adolescents: longitudinal and secular trends from 1999 to 2004. Preventive medicine. 2006;43(1):52-9.
49. Neumark-Sztainer D, Patterson J, Mellin A, Ackard DM, Utter J, Story M, et al. Weight control practices and disordered eating behaviors among adolescent females and males with type 1 diabetes: associations with sociodemographics, weight concerns, familial factors, and metabolic outcomes. Diabetes care. 2002;25(8):1289-96.
50. Neumark-Sztainer D, Wall M, Larson NI, Eisenberg ME, Loth K. Dieting and disordered eating behaviors from adolescence to young adulthood: findings from a 10-year longitudinal study. Journal of the American Dietetic Association. 2011;111(7):1004-11.
51. Nishijima C, Kobayashi E, Sato Y, Chiba T. A nationwide survey of the attitudes toward the use of dietary supplements among Japanese high-school students. Nutrients. 2019;11(7).
52. Nobakht M, Dezhkam M. An epidemiological study of eating disorders in Iran. Int J Eat Disord. 2000;28(3):265-71.
53. Nunes MA, Barros FC, Anselmo Olinto MT, Camey S, Mari JDJ. Prevalence of abnormal eating behaviours and inappropriate methods of weight control in young women from Brazil: a population-based study. Eat Weight Disord. 2003;8(2):100-6.

© 2024 Hall NY et al. *JAMA Network Open*.

54. Papas MA, Trabulsi JC, Axe M, Rimmer JH. Predictors of Obesity in a US Sample of High School Adolescents With and Without Disabilities. Journal of School Health. 2016;86(11):803-12.
55. Park MJ, Kang YJ, Kim DH. Dissatisfactioin with height and weight, and attempts at height gain and weight control in Korean school-children. Journal of pediatric endocrinology & metabolism : JPEM. 2003;16(4):545-54.
56. Paxton SJ, Wertheim EH, Gibbons K, Szmukler GI, Hillier L, Petrovich JL. Body image satisfaction, dieting beliefs, and weight loss behaviors in adolescent girls and boys. Journal of Youth and Adolescence. 1991;20(3):361-79.
57. Eleonora PP, Carla MM, Simona FF, Angelica SS, Umberto FF, Brunella II, et al. Disturbed eating behavior in pre-teen and teenage girls and boys with type 1 diabetes. Acta bio-medica de l'Ateneo Parmense. 2019;89(4):490-7.
58. Pernick Y, Nichols JF, Rauh MJ, Kern M, Ji M, Lawson MJ, et al. Disordered eating among a multi-racial/ethnic sample of female high-school athletes. J Adolesc Health. 2006;38(6):689-95.
59. Peveler RC, Fairburn CG, Boller, Dunger D. Eating disorders in adolescents with IDDM: a controlled study. Diabetes Care. 1992;15(10):1356-60.
60. Phelps L, Andrea R, Rizzo FG, Johnston L, Main CM. Prevalence of self-induced vomiting and laxative/medication abuse among female adolescents: A longitudinal study. International Journal of Eating Disorders. 1993;14(3):375-8.
61. Pollina-Pocallet M, Artigues-Barberà E, Tort-Nasarre G, Sol J, Azlor L, Foguet-Boreu Q, et al. Self-Perception and Self-Acceptance Are Related to Unhealthy Weight Control Behaviors in Catalan Adolescents: A Cross-Sectional Study. Int J Environ Res Public Health. 2021;18(9).
62. Rosen JC, Gross J. Prevalence of weight reducing and weight gaining in adolescent girls and boys. Health psychology : official journal of the Division of Health Psychology, American Psychological Association. 1987;6(2):131-47.
63. Rosenbaum JE. Truth or consequences: the intertemporal consistency of adolescent self-report on the Youth Risk Behavior Survey. American journal of epidemiology. 2009;169(11):1388-97.
64. Santos CdFBF, Godoy F, de Menezes VA, Colares V, de Araújo Zarzar PMP, Ferreira RC, et al. LGB prevalence in schools is associated with unhealthy weight-control behaviors in lesbian, gay, and bisexual youth: a multilevel analysis. BMC Public Health. 2021;21(1):1256.
65. Serdula MK, Collins ME, Williamson DF, Anda RF, Pamuk E, Byers TE. Weight control practices of U.S. adolescents and adults. Annals of Internal Medicine. 1993;119(7 II):667-71.
66. Mashego T-AB, Senekal M, Steyn NP, Nel JH. Evaluation of body shape, eating disorders and weight management related parameters in black female students of rural and urban origins. South African journal of psychology. 2001;31(1):45-53.
67. Silva SUd, Barufaldi LA, Andrade SSCdA, Santos MAS, Claro RM. Nutritional status, body image, and their association with extreme weight control behaviors among Brazilian adolescents, National Adolescent Student Health Survey 2015. Revista brasileira de epidemiologia = Brazilian journal of epidemiology. 2018;21(suppl 1):e180011.
68. Stephen EM, Rose JS, Kenney L, Rosselli-Navarra F, Weissman RS. Prevalence and correlates of unhealthy weight control behaviors: findings from the national longitudinal study of adolescent health. Journal of eating disorders. 2014;2:16.
69. Stigler MH, Arora M, Dhavan P, Shrivastav R, Reddy KS, Perry CL. Weight-related concerns and weight-control behaviors among overweight adolescents in Delhi, India: a cross-sectional study. The international journal of behavioral nutrition and physical activity. 2011;8:9.
70. Story M, French SA, Resnick MD, Blum RW. Ethnic/racial and socioeconomic differences in dieting behaviors and body image perceptions in adolescents. The International journal of eating disorders. 1995;18(2):173-9.
71. Sutter ME, Nasim A, Veldheer S, Cobb CO. Associations between unhealthy dieting behaviors and tobacco use among adolescents. Journal of eating disorders. 2016;4:39.

© 2024 Hall NY et al. *JAMA Network Open*.

72. Swanson SA, Aloisio KM, Horton NJ, Sonneville KR, Crosby RD, Eddy KT, et al. Assessing eating disorder symptoms in adolescence: Is there a role for multiple informants? International Journal of Eating Disorders. 2014;47(5):475-82.
73. Stevens J, Cornell CE, Story M, French SA, Levin S, Becenti A, et al. Development of a questionnaire to assess knowledge, attitudes, and behaviors in American Indian children. The American journal of clinical nutrition. 1999;69(4):773S-81S.
74. Stock SL, Goldberg E, Corbett S, Katzman DK. Substance use in female adolescents with eating disorders. J Adolesc Health. 2002;31(2):176-82.
75. Talamayan KS, Springer AE, Kelder SH, Gorospe EC, Joye KA. Prevalence of overweight misperception and weight control behaviors among normal weight adolescents in the United States. TheScientificWorldJOURNAL. 2006;6:928018.
76. Tăut D, Chițu C, Băban A. Self-reported weight and body weight dissatisfaction: Their conjoint role in dieting and health complaints of adolescents. Psihologijske Teme. 2018;27(1):125-39.
77. Thorlton JR, McElmurry B, Park C, Hughes T. Adolescent performance enhancing substance use: regional differences across the US. Journal of addictions nursing. 2012;23(2):97-111.
78. Trigazis L, Tennankore D, Vohra S, Katzman DK. The use of herbal remedies by adolescents with eating disorders. International Journal of Eating Disorders. 2004;35(2):223-8.
79. Tuffa TA, Gebreyesus SH, Endris BS, Getnet Y, Abebe DS. Unhealthy weight control behaviors among Ethiopian female adolescents. Int J Eat Disord. 2020;53(4):525-32.
80. Tur-Sinai A, Kolobov T, Tesler R, Baron-Epel O, Harel-Fisch Y. Associations Between Socioeconomic and Family Determinants and Weight-Control Behaviours among Adolescents. International Journal of Public Health. 2020.
81. Utter J, Denny S, Percival T, Crengle S, Ameratunga S, Dixon R, et al. Prevalence of weight-related concerns and behaviours among New Zealand young people. Journal of Paediatrics and Child Health. 2012;48(11):1021-8.
82. Ursoniu S, Putnoky S, Vlaicu B. Body weight perception among high school students and its influence on weight management behaviors in normal weight students: a cross-sectional study. Wien Klin Wochenschr. 2011;123(11-12):327-33.
83. Vervaet M, Heeringen CV. Eating Style and Weight Concerns in Young Females. Eating disorders. 2000;8(3):233-40.
84. Vidot DC, Messiah SE, Prado G, Hlaing WM. Relationship Between Current Substance Use and Unhealthy Weight Loss Practices Among Adolescents. Matern Child Health J. 2016;20(4):870-7.
85. Visser J, Notelovitz T, Szabo CP, Fredericks N. Abnormal eating attitudes and weight-loss behaviour of adolescent girls attending a “traditional” Jewish high school in Johannesburg, South Africa. South African Journal of Clinical Nutrition. 2014;27(4):208-16.
86. Wheaton AG, Perry GS, Chapman DP, Croft JB. Self-reported sleep duration and weight-control strategies among U.S. high school students. Sleep. 2013;36(8):1139-45.
87. Waaddegaard M, Petersen T. Dieting and desire for weight loss among adolescents in Denmark: A questionnaire survey. European Eating Disorders Review. 2002;10(5):329-46.
88. Watson RJ, VanKim NA, Rose HA, Porta CM, Gahagan J, Eisenberg ME. Unhealthy weight control behaviors among youth: Sex of sexual partner is linked to important differences. Eat Disord. 2018;26(5):448-63.
89. Watson RJ, Adjei J, Saewyc E, Homma Y, Goodenow C. Trends and disparities in disordered eating among heterosexual and sexual minority adolescents. Int J Eat Disord. 2017;50(1):22-31.
90. Zhang J, Seo D-C, Kolbe L, Lee A, Middlestadt S, Zhao W, et al. Comparison of overweight, weight perception, and weight-related practices among high school students in three large Chinese cities and two large U.S. cities. The Journal of adolescent health : official publication of the Society for Adolescent Medicine. 2011;48(4):366-72.

© 2024 Hall NY et al. *JAMA Network Open*.
