# Supplementary material for: Global Prevalence of Adolescent Use of Nonprescription Weight-Loss Products: A Systematic Review and Meta-Analysis
Source: JAMA Netw Open. 2024 Jan 10;7(1):e2350940. doi: 10.1001/jamanetworkopen.2023.50940 (PMC10782242; doi:10.1001/jamanetworkopen.2023.50940)
Supplement: Supplement 2. — Data Sharing Statement [file jamanetwopen-e2350940-s002.pdf]

Data Sharing Statement

Hall. Global Prevalence of Adolescent Use of Nonprescription Weight-Loss Products. *JAMA Netw Open*. Published January 10, 2024. doi:10.1001/jamanetworkopen.2023.50940

Data
Data available: Yes
Data types: Other (please specify)
Additional Information: All studies that are part of the meta-analysis are provided
How to access data: Appendix 1
When available: With publication

Supporting Documents
Document types: None

Additional Information
Who can access the data: anyone requesting the data - The data used for the meta-analysis is all publicly available data
Types of analyses: The data used for the meta-analysis is all publicly available data
Mechanisms of data availability: with investigator support
